# Assessing reproducibility of inherited variants detected with short-read whole genome sequencing

**DOI:** 10.1186/s13059-021-02569-8

**Published:** 2022-01-03

**Authors:** Bohu Pan, Luyao Ren, Vitor Onuchic, Meijian Guan, Rebecca Kusko, Steve Bruinsma, Len Trigg, Andreas Scherer, Baitang Ning, Chaoyang Zhang, Christine Glidewell-Kenney, Chunlin Xiao, Eric Donaldson, Fritz J. Sedlazeck, Gary Schroth, Gokhan Yavas, Haiying Grunenwald, Haodong Chen, Heather Meinholz, Joe Meehan, Jing Wang, Jingcheng Yang, Jonathan Foox, Jun Shang, Kelci Miclaus, Lianhua Dong, Leming Shi, Marghoob Mohiyuddin, Mehdi Pirooznia, Ping Gong, Rooz Golshani, Russ Wolfinger, Samir Lababidi, Sayed Mohammad Ebrahim Sahraeian, Steve Sherry, Tao Han, Tao Chen, Tieliu Shi, Wanwan Hou, Weigong Ge, Wen Zou, Wenjing Guo, Wenjun Bao, Wenzhong Xiao, Xiaohui Fan, Yoichi Gondo, Ying Yu, Yongmei Zhao, Zhenqiang Su, Zhichao Liu, Weida Tong, Wenming Xiao, Justin M. Zook, Yuanting Zheng, Huixiao Hong

**Affiliations:** 1 Division of Bioinformatics and Biostatistics, National Center for Toxicological Research, US Food and Drug Administration, Jefferson, AR 72079 USA; 2 State Key Laboratory of Genetic Engineering, School of Life Sciences and Shanghai Cancer Center, Fudan University, Shanghai, 200438 China; 3 Human Phenome Institute, Fudan University, Shanghai, 200438 China; 4 Illumina Inc., San Diego, CA 92122 USA; 5 SAS Institute Inc., Cary, NC 27513 USA; 6 Immuneering Corporation, Cambridge, MA 02142 USA; 7 Real Time Genomics, Hamilton, New Zealand; 8 Institute for Molecular Medicine Finland (FIMM), University of Helsinki, Helsinki, Finland; 9 EATRIS ERIC- European Infrastructure for Translational Medicine, Amsterdam, the Netherlands; 10 School of Computing Sciences and Computer Engineering, University of Southern Mississippi, Hattiesburg, MS 39406 USA; 11 National Center for Biotechnology Information, National Library of Medicine, National Institutes of Health, Bethesda, MD 20894 USA; 12 Center for Drug Evaluation and Research, Food and Drug Administration, Silver Spring, MD 20993 USA; 13 Human Genome Sequencing Center, Baylor College of Medicine, Houston, TX 77030 USA; 14 Sentieon Inc., San Jose, CA 95134 USA; 15 Center for Advanced Measurement Science, National Institute of Metrology, Beijing, 100013 China; 16 Department of Physiology and Biophysics, Weill Cornell Medicine, New York, NY 10021 USA; 17 Roche Sequencing Solutions, Santa Clara, CA 95050 USA; 18 Bioinformatics and Computational Biology Laboratory, National Heart Lung and Blood Institute, National Institutes of Health, Bethesda, MD 20892 USA; 19 Environmental Laboratory, U.S. Army Engineer Research and Development Center, Vicksburg, MS 39180 USA; 20 Office of Health Informatics, Office of the Commissioner, US Food and Drug Administration, Silver Spring, MD 20993 USA; 21 The Center for Bioinformatics and Computational Biology, Shanghai Key Laboratory of Regulatory Biology, Institute of Biomedical Sciences and School of Life Sciences, East China Normal University, Shanghai, 200241 China; 22 Stanford Genome Technology Center, Stanford University School of Medicine, Palo Alto, CA 94305 USA; 23 Pharmaceutical Informatics Institute, College of Pharmaceutical Sciences, Zhejiang University, Hangzhou, 310058 China; 24 Department of Molecular Life Sciences, Tokai University School of Medicine, 143 Shimokasuya, Isehara, 259-1193 Japan; 25 CCR-SF Bioinformatics Group, Advanced Biomedical and Computational Sciences, Biomedical Informatics and Data Science, Frederick National Laboratory for Cancer Research, Frederick, MD 21701 USA; 26 Takeda Pharmaceuticals, Cambridge, MA 02139 USA; 27 Division of Molecular Genetics and Pathology, Center for Device and Radiological Health, US Food and Drug Administration, Silver Spring, MD 20993 USA; 28 Material Measurement Laboratory, National Institute of Standards and Technology, Gaithersburg, MD 20899 USA

## Abstract

**Background:**

Reproducible detection of inherited variants with whole genome sequencing (WGS) is vital for the implementation of precision medicine and is a complicated process in which each step affects variant call quality. Systematically assessing reproducibility of inherited variants with WGS and impact of each step in the process is needed for understanding and improving quality of inherited variants from WGS.

**Results:**

To dissect the impact of factors involved in detection of inherited variants with WGS, we sequence triplicates of eight DNA samples representing two populations on three short-read sequencing platforms using three library kits in six labs and call variants with 56 combinations of aligners and callers. We find that bioinformatics pipelines (callers and aligners) have a larger impact on variant reproducibility than WGS platform or library preparation. Single-nucleotide variants (SNVs), particularly outside difficult-to-map regions, are more reproducible than small insertions and deletions (indels), which are least reproducible when > 5 bp. Increasing sequencing coverage improves indel reproducibility but has limited impact on SNVs above 30×.

**Conclusions:**

Our findings highlight sources of variability in variant detection and the need for improvement of bioinformatics pipelines in the era of precision medicine with WGS.

**Supplementary Information:**

The online version contains supplementary material available at 10.1186/s13059-021-02569-8.

## Background

Inherited variants drive susceptibility to diseases spanning oncology [1], central nervous system [2], inflammatory [3], autoimmune [4], and rare diseases [5] plus many more. Reproducible detection of inherited variants enables a better translation of findings from genetic studies into clinical practice via disease diagnosis [1], disease risk assessment [6], and drug development [7]. Whole genome sequencing (WGS) is increasingly used for inherited variant detection due to decreasing cost, single-nucleotide level resolution of nearly the entire human genome, and decreased error rates [8]. However, accurate WGS inherited variant calling is confronted by many challenges. The human genome contains regions of varying complexity, meaning that robust calling in some regions is more difficult than others [9]. Adding to this challenge, sequencing coverage is often uneven across the genome, particularly for targeted sequencing [10, 11]. Regions with more coverage by correctly mapped reads result in more confident calling [10, 12]. Library preparation and sequencing chemistry itself can produce errors, and if these errors accumulate, they lead to false variant calls [13]. Although aligners and variant callers have undergone great improvements in recent years, this process remains error prone, especially in highly repetitive genome regions. Understanding inherited variant reproducibility issues will lead to improved quality for future WGS studies.

To date, efforts such as the Genome in a Bottle Consortium (GIAB) [14], Platinum Genomes Project (PG) [15], and Syndip [16] have produced benchmark or “truth” variant calls and regions against which bioinformatics pipelines can be compared and tested, using publicly available cell lines for GIAB and PG. The Global Alliance for Genomics and Health (GA4GH) recently published a framework for benchmarking variant calling, including standardization of performance metrics [17]. The precisionFDA held two public challenges in 2016 (https://precision.fda.gov/challenges/) for comparing performance of various inherited variant calling pipelines (“consistency” challenge and “truth” challenge). Moreover, several previous studies focused on investigating the impact of potential factors including platform [18–20] or pipeline [21, 22] on genomic variants calling. However, a systematic examination of these factors, together with sequencing platforms, labs, replicates, and DNA samples of different populations is lacking. Here, we seek to further characterize the role of bioinformatics pipelines and interaction with upstream wet lab performance on inherited variant calling. We sequenced triplicates of genomic DNA samples from a Caucasian HapMap trio [23], a well-characterized Chinese quartet from The Quartet Project for Quality Control and Data Integration of Multi-omics Profiling (http://chinese-quartet.org/), and NA12878 used in GIAB [24] using various library preparation kits and sequencing instruments in multiple labs. Inherited variants were called with combinations of multiple aligners and callers.

Via combinations of wet lab experimental and bioinformatics approaches, we assessed the impact of factors involved in variant detection with WGS and their interactions on variant reproducibility for both small variant and structural variant [25]. We found that current sequencing wet lab components including sample preparation, library generation, and sequencing (platform and labs) are much more reproducible than bioinformatics components such as alignment and variant calling, demonstrating the key need of improving bioinformatics analysis in WGS for precision medicine. Our findings highlight the importance of harmonization and could enhance inherited variant calling research across diseases, therapeutic areas, and institutions.

## Results

### Study design and data generation

**Fig. 1:**
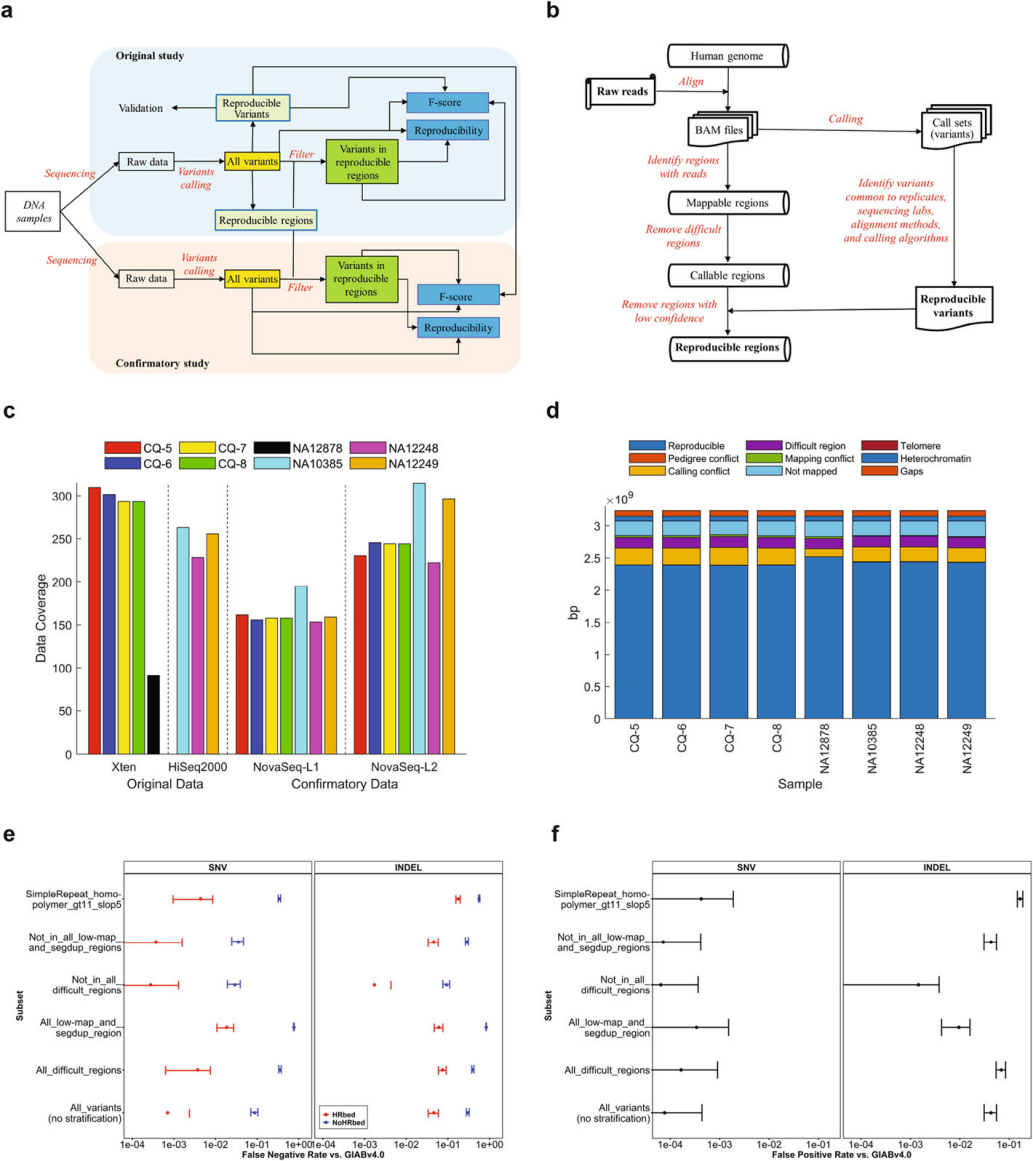
Study design and highly reproducible regions (HRR). **a** Study design. The DNA samples are from the Chinese quartet, the HapMap trio, and NA12878. WGS was conducted on the samples using different platforms and library preparation kits in multiple labs in the original study (light blue background) and confirmatory study (light brown background). Various variant calling pipelines were employed to generate variants (yellow boxes) from the raw sequence data. The variants were leveraged to define the HRR and pinpoint HRVs (light green boxes). Reproducibility (blue boxes) was analyzed for both all variants and the variants only in HRR (green boxes) in both original and confirmatory studies. The variants with and without HRR-filtering were compared with the HRVs to calculate F-scores (blue boxes), which were used to evaluate reproducibility from a different angle. **b** Process for defining HRR. All alignment results for the same sample were first examined to find the genomic regions that have sequence reads mapped. Difficult regions such as repeats were then removed to form the callable regions. At last, the HRVs obtained from comparative analysis on all call sets were used to remove the low confidence calling regions from the callable regions, resulting the HRR. **c** Data generated. Sequencing data coverage is on the *y*-axis for DNA samples. Original and confirmatory data sets are separated with the vertical solid line and depicted with the *x*-axis label. The four Illumina sequencing platforms are separated with the vertical dashed lines and marked on the *x*-axis ticks where L1 indicates the Nextera DNA Flex library preparation kit and L2 is the TruSeq DNA PCR-Free Library Prep Kit. The color legend indicates samples. **d** Sizes (*y*-axis) of HRR (dark blue bars) for the 8 samples (*x*-axis). The color legend shows the excluded genomic regions, including gap region (dark brown) not in GRCh38, heterochromatin (blue) for condensed DNA labeled as *N* in the reference, telomere (dark purple) for repeat sequence at the end of the chromosome, not mapped region (light blue), mapping conflict region (green), difficult region (purple) for repeat regions (“SimpleRepeat_imperfecthomopolgt10_slop5.BED” and “remapped_superdupsmerged_all_sort.BED”) defined by GA4GH and GIAB, calling conflict region (yellow) for the flanking region of discordant variants, and pedigree conflict region (brown). **e** False negative rates (FN/(TP + FN)) of HRVs for NA12878 against the GIAB v4.0 benchmark set and stratified by genome context for SNVs (the left panel) and indels (the right panel) in the entire v4.0 benchmark regions (blue) and confined to the HRR (red). Error bars indicate 95% confidence intervals. **f** False positive rates (FP/(TP + FP)) of HRVs stratified by genome context in the entire v4.0 benchmark regions. Error bars indicate 95% confidence intervals

We designed a study (Fig. 1 a) to systematically evaluate the reproducibility of inherited variants detected with short-read WGS. More than 109 billion short reads at various coverages were generated from eight DNA samples including a Chinese quartet (CQ-5, CQ-6, CQ-7, and CQ-8), a HapMap trio (NA10385, NA12248, and NA12249), and NA12878 used in GIAB [24] using multiple sequencing platforms and library preparations at different sequencing labs (Fig. 1 c, Additional file 1, Table S1). Fifty-six combinations of different aligners and callers (Additional file 2, Table S2) were used to call SNVs and small indels (Additional files 3-5, Tables S3-S5). The variants were used to assess reproducibility, spanning factors such as sequencing platform/lab/library preparations, alignment, and calling. The variants without filtering (all called variants in Fig. 1 a) were used for evaluation of the reproducibility of variants in all genomic regions without any filtering, hereafter termed as the lower bound of reproducibility.

To estimate the upper bound, highly reproducible variants (HRVs) (Additional file 6, Table S6) and corresponding highly reproducible regions (HRR) (Additional file 7, Table S7) were defined for the eight samples (Fig. 1 d) using our workflow (Fig. 1 b, Additional file 8: Fig. S1). Of the highly reproducible SNVs, 1.52 to 1.57% were in coding regions (Additional file 8: Fig. S2), indicating SNVs from the coding and non-coding regions do not have substantial differences as the size of coding regions accounts for a similar fraction (approximately 1.5%) of whole human genome [26]. The G/C frequencies of the highly reproducible SNVs are markedly higher than those of human genome, consistent with the findings from the international HapMap Consortium and the 1000 Genomes Project [27]. Moreover, the G/C content of the highly reproducible SNVs in coding regions is higher than non-coding regions, which is supported by the fact that coding regions contain a higher G/C content than non-coding regions [28]. In contrast, a higher fraction of insertions (Additional file 8: Fig. S3) and deletions (Additional file 8: Fig. S4) are in non-coding regions compared with SNVs, perhaps due to fewer homopolymer and tandem repeats as well as selection against truncating indels in coding regions [29]. Intriguingly, insertions and deletions are comparatively G/C-poor, especially for non-coding regions. This may reflect the increased indel rate in homopolymers, since A/T homopolymers are more common in the human genome than G/C homopolymers.

All called variants were filtered using the HRR and the resulting variants in the reproducible regions were used to assess the upper bound of reproducibility (Fig. 1 a). In addition, all called variants and the variants in reproducible regions were compared with the HRVs to calculate F-scores. These F-scores then were used to evaluate reproducibility.

To confirm the observed trends in reproducibility from our original study, the same DNA samples were whole genome sequenced using a different Illumina sequencing platform (Illumina NovaSeq) and different library preparations (Illumina Nextera DNA Flex, “Nextera” hereafter, and Illumina TruSeq, “TruSeq” hereafter). Inherited variants were called from the confirmatory sequencing data using the same bioinformatics pipelines. Both lower and upper bounds of reproducibility from the confirmatory study were evaluated in the same way (Fig. 1 a).

### Impact of variant class and genome context on reproducibility

To understand the characteristics of the HRVs, we used GA4GH Benchmarking tools [17] to compare the HRVs to a new v4.0 draft GIAB benchmark for NA12878 [30], which uses long reads and linked reads to make calls in more difficult regions. The recall (or sensitivity) of the HRVs clearly varies by variant size and genome context (Fig. 1 e). The HRVs matched 97.3% of benchmark SNVs after (vs. 91% before) excluding all GA4GH-defined difficult regions and complex variants [31], and the false positive (FP) rate is very low at 0.007% (Fig. 1 f). Most of the SNVs that were not highly reproducible were in regions difficult to map with short reads and in segmental duplications, since 174,721 of the 281,232 false negative (FN) SNVs fall in these regions. FNs also were highly enriched in L1H regions > 500 bp and > 75% G/C content. For indels, the FN rate was higher (~ 30%), because, in addition to difficult to map regions, there are several categories of variants that were not highly reproducible, including homopolymers, tandem repeats, indels > 6 bp in size, and complex variants. In total, 119,882 of 144,151 FN indels were in homopolymers or tandem repeats, including 77,083 in homopolymers longer than 6 bp or imperfect homopolymer longer than 10 bp, 35,266 in tandem repeats shorter than 51 bp, 15,992 in tandem repeats 51 bp to 200 bp long, and 4014 in tandem repeats longer than 200 bp (Additional file 9, Table S8). More FNs likely exist outside the v4.0 benchmark regions, since it still excludes many long homopolymers, short tandem repeats, and variable number tandem repeats. For indels, the FP rate was higher (~ 4.3%), with most FPs occurring in complex variants, particularly in homopolymers and tandem repeats. The indel FP rate was < 0.2% after excluding difficult regions and complex variants. Within the HRR for NA12878, the SNV FN rate was 0.07%, SNV FP rate was 0.008%, indel FN rate was 4.6%, and indel FP rate was 4.3%, though if genotype errors were excluded then the indel FN was 0.3% and the indel FP rate was 0.04%.

### Factors impacting reproducibility

**Fig. 2:**
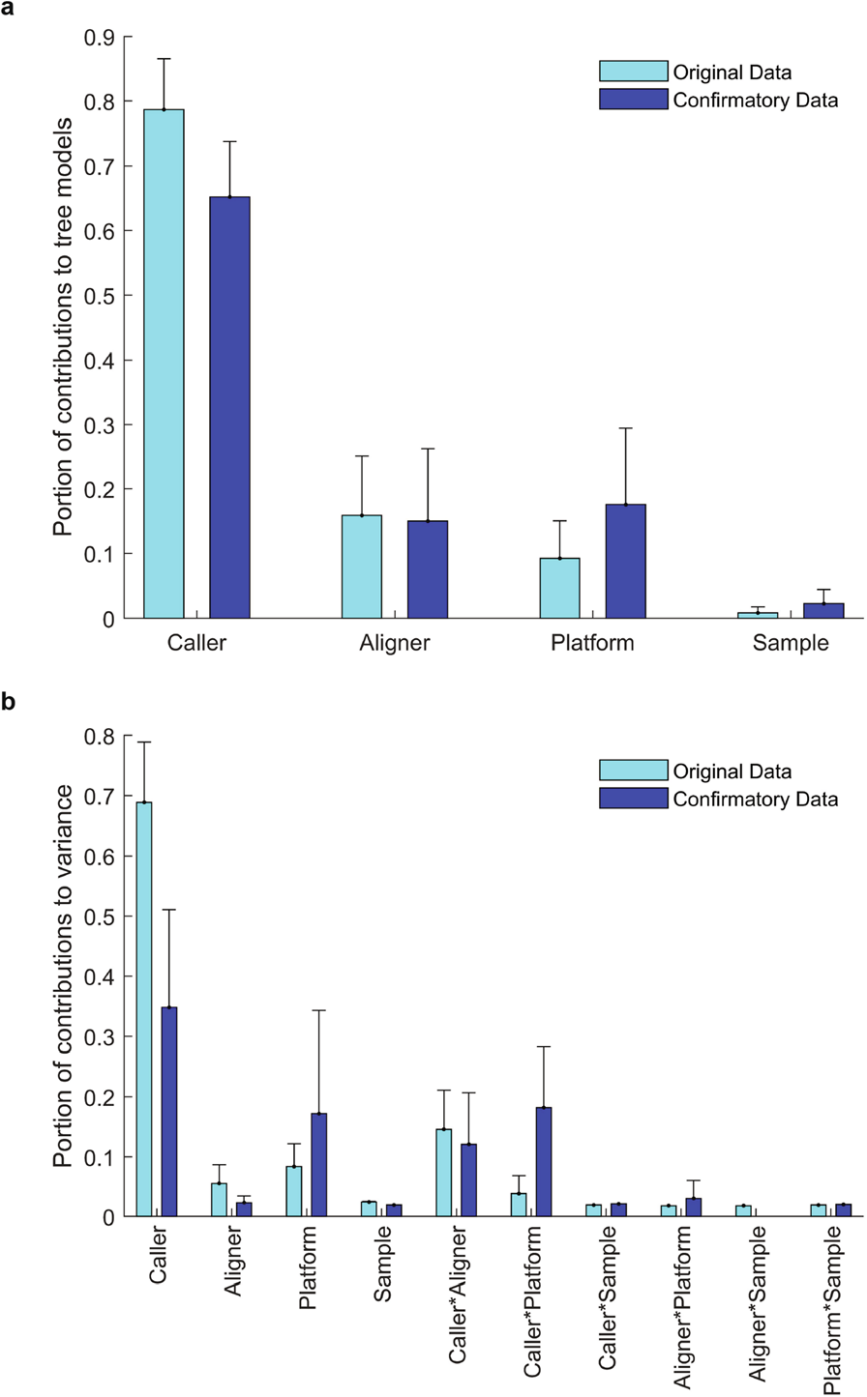
Impacts of factors on variant reproducibility. **a** Contributions to gradient boosted trees. The four factors are depicted at the *x*-axis and portions of their contributions to the non-linear gradient boosted tree models are the light blue bars for the original study and are the dark blue bars for the confirmatory study. The error bars are the standard deviations of the portions from different data sets (Additional file 14, Table S13). **b** Contributions to reproducibility in variance. The contributions of the four factors as well as their 2-way interactions (depicted at *x*-axis) from variance components analysis are plotted as light blue bars for the original study and dark blue bars for the confirmatory study. The error bars are standard deviations of the portions from different data sets (Additional file 15, Table S14)

Multiple factors including caller [21, 22], aligner [32, 33], sequencing platform/lab/library [18, 20, 34] preparation (combined in analysis and simply termed as “platform” hereafter), and sample could affect reproducibility of inherited variants. To assess the impact of these factors, average Jaccard index values among the inherited variants from triplicate DNA samples were first calculated and then analyzed using gradient boosted classification tree to evaluate the contributions of these factors to the trees (Additional file 10, Table S9). For variants with and without HRR filtering, more than 60% of contributions came from callers. Aligners were the second largest contributor (Fig. 2 a). Sequencing platform was the third largest contributor, contributing to more variability for insertions and deletions than SNVs (Additional file 10, Table S9). DNA samples had limited impact on reproducibility, suggesting that any of these eight DNA samples could be used for assessing reproducibility. The observed impacts from the original study were replicated in the confirmatory study (Fig. 2 a). Interestingly, the contributions of caller, platform, and caller × platform in the confirmatory study were much larger than in the original study. This might be caused by the difference in library preparation kits included in the combined factor platform. All original data were generated using the same library kit TruSeq, while the confirmatory data were generated using two library kits TruSeq and Nextera. Thus, the impacts of platform and caller × platform on the confirmatory data are larger than on the original data. As all variances for original data or confirmatory data were summed up to 100%, the relative caller impact on the confirmatory data was decreased due to the increase in impacts of platform and caller × platform. This observation indicates that the contribution of platform to variability may depend on the spectrum of sequencing instruments and library preparations tested.

The gradient boosted classification tree analysis did not separate the impact of interactions between these factors. To further ascertain sources of variance in reproducibility, joint effects of these factors were examined using variance component analysis (Additional file 11, Table S10). The impacts of these factors were in the same order as obtained from the gradient boosted tree analysis: caller > aligner > platform > DNA sample (Fig. 2 b). Furthermore, caller had a large joint effect with aligner. Intriguingly, library preparation (for the confirmatory study) and caller had a considerable joint effect, especially for indels (Additional file 11, Table S10). Again, DNA samples not only had limited contributions to the variance, but also had small joint effects with other factors.

### Technical reproducibility

**Fig. 3:**
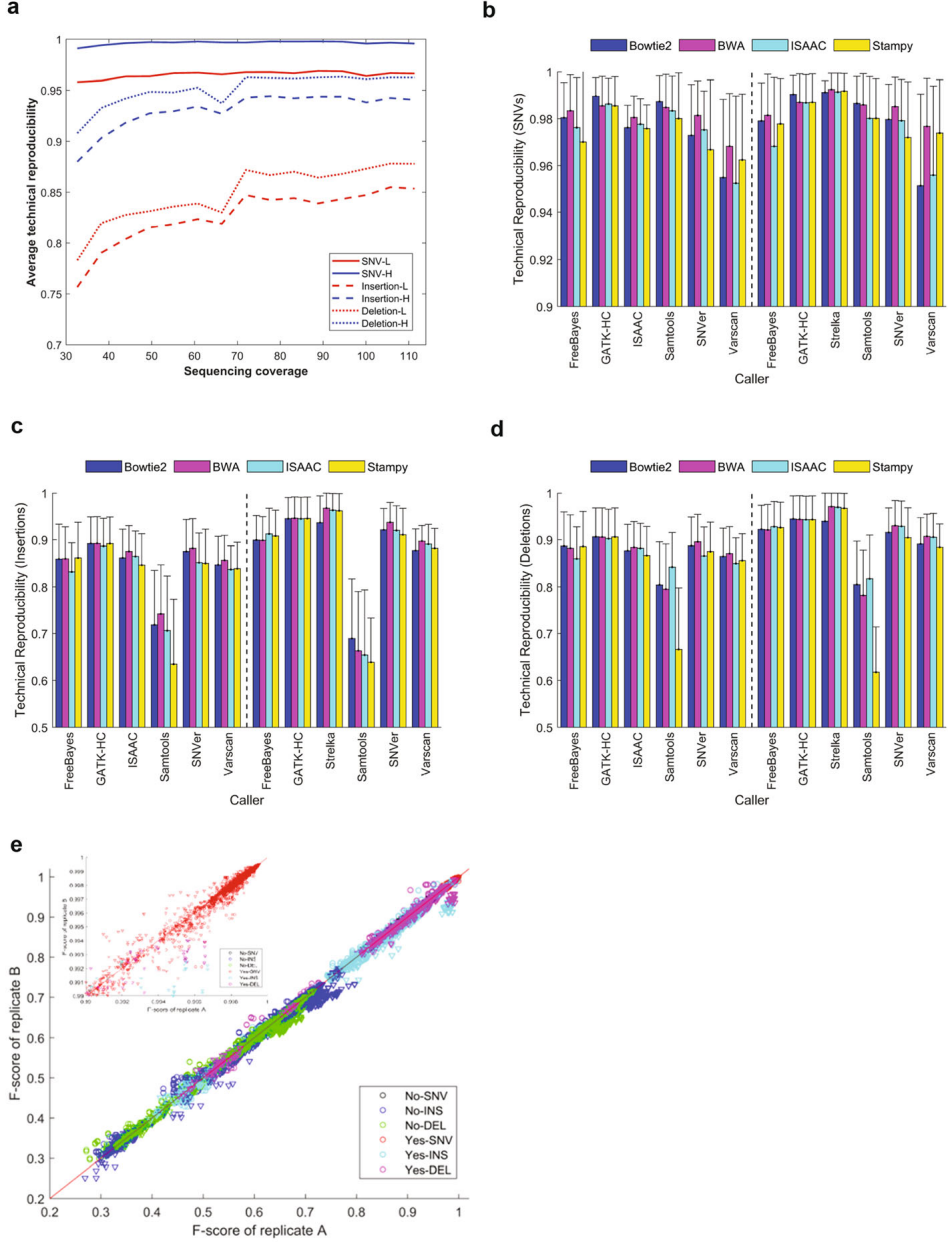
Technical reproducibility. **a** Impact of sequencing coverage on technical reproducibility. Average technical reproducibility (*y*-axis) of detected variants is plotted against the sequencing coverage (*x*-axis). Line types indicate variant types (SNVs: solid lines, insertion: dash lines, deletion: dot line). Red lines represent upper bounds of technical reproducibility and blue lines are lower bounds of technical reproducibility. **b**,**c,d** Technical reproducibility across aligners and callers for SNVs (**b**), insertions (**c**), and deletions (**d**). The average technical reproducibility of variants for pairs of callers (*x*-axis) and aligners (color legend) are plotted as bars with their standard deviation as sticks. The left panels give the results from the original data and the right panels show the results from the confirmatory data. **e** F-scores of technical replicates. The F-scores from one technical replicate (*x*-axis) are plotted against the F-scores from another technical replicate (*y*-axis). The marker colors represent types of variants indicated at the right bottom corner with two-word text. The first indicates HRR filtering (Yes and No) and the second for variant type (SNV: SNVs, INS: insertions, DEL: deletions). The downward triangles represent the F-scores from the original study, while the circles mark the F-scores from the confirmatory study. The inserted figure at top left is a zoom-in of the F-score > 0.99 region

Analysis of the impact of individual factors on reproducibility was performed to identify potential areas to establish good practices for WGS inherited variant detection. We first assessed technical reproducibility by measuring the concordance of inherited variants between triplicates (Additional file 12, Table S11). The technical reproducibility distributions of SNVs, insertions, and deletions (Additional file 8: Fig. S5-S7) revealed that SNVs were more reproducible than indels. The distributions also indicated a large variation in the technical reproducibility of different calling pipelines. We found that sequencing coverage had limited impact on technical reproducibility of SNVs detected with WGS at >30× coverage, while increasing sequencing coverage improved technical reproducibility of indels (Fig. 3 a), especially when sequencing coverage was increased from 30× to 70×. Both lower and upper bounds of reproducibility of replicate pairs were consistent and not dependent on samples, sequencing platforms, and labs (Additional file 8: Fig. S8-S15).

Variance analysis showed callers and aligners were the two largest components (Fig. 2) but could not reveal performance of individual callers and aligners. Therefore, we examined technical reproducibility values for individual callers and aligners. The aligners performed similarly (Additional file 8: Fig. S16), with Stampy having notably lower technical reproducibility for indels than other aligners, especially with Samtools (Fig. 3 c, d). The technical reproducibility differences between the original and confirmatory studies for SNVs, insertions, and deletions were 0.4% (95% confidence: 0.3 to 0.5%), 4.4% (3.8 to 4.9%), and 3.7% (3.2 to 4.2%), respectively, providing confidence in the reliability of the technical reproducibility evaluation.

Technical reproducibility comparisons found that the callers had much more variable performance (Additional file 8: Fig. S17), consistent with the variance analysis. More specifically, SNVs from VarScan were less reproducible than SNVs from other callers (Fig. 3 b). Interestingly, VarScan yielded similarly reproducible indels as other callers, while Samtools generated less reproducible indels (Fig. 3 c, d).

Comparison of the F-scores between the triplicates for SNVs and indels (Fig. 3 e) resulted in similar observations. F-scores of technical replicates were generally reproducible with a correlation coefficient *r* = 0.993 and not dependent on samples, variant types, sequencing platforms, and labs as well as calling pipelines.

### Lab reproducibility

**Fig. 4:**
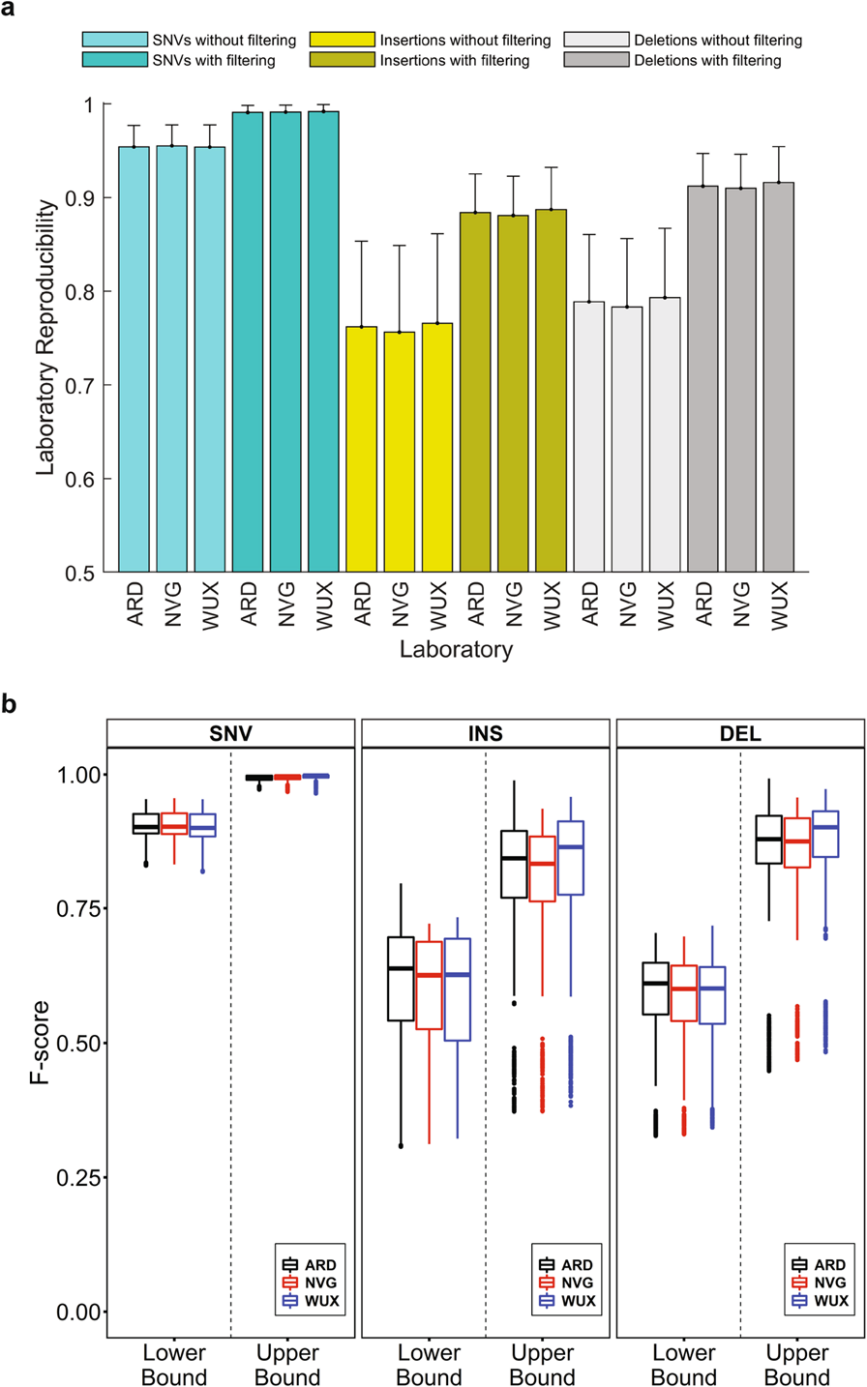
Lab reproducibility. **a** Lab reproducibility of the Chinese quartet samples in the original study. The bars represent average values of lab reproducibility and the error sticks indicate standard deviations. The *x*-axis ticks depict sequencing labs. The color legend represents variant types and HRR filtering status. **b** Boxplots of F-scores for SNVs (left panel), insertions (middle panel), and deletions (right panel). Results from the three labs are plotted in different colors: black for ARD (Annoroad), red for WUX (WuXi NextCODE), and blue for NVG (NovoGene). F-scores from the lower bound and upper bound of variants are separated and marked at *x*-axis

To assess lab reproducibility, the Chinese quartet DNA samples were sequenced in three labs in our original study. We calculated reproducibility of the inherited variants detected across the three labs (Additional file 13, Table S12). The lower and upper bounds of lab reproducibility for SNVs were ~ 0.95 and > 0.99 (Fig. 4 a), demonstrating that current WGS methods vary by reproducibility across labs for SNVs. Lab reproducibility for indels was much lower with the lower bound of 0.75 to 0.78 and the upper bound of 0.89 to 0.91 (Fig. 4 a), indicating relatively large room for improvement for indel detection with current WGS. Reproducibility of technical replicates within a lab was consistent among different labs for all three variant types (Additional file 8: Fig. S18 and S19), demonstrating that reproducibility did not vary by lab. To pinpoint causes for the non-reproducible portion, we compared the lab reproducibility for DNA samples, aligners, and callers (Additional file 8: Fig. S20). Consistent with previous work^21,22^, callers were the major cause of lab reproducibility variance, followed by aligners. Reproducibility did not vary much by sample. SNVs from VarScan and indels from Samtools were the least reproducible across the sequencing labs. The reproducibility of variants from different aligners within each lab was similar between labs (Additional file 8: Fig. S21 and S22), further demonstrating that reproducibility did not vary substantially by lab.

Lab reproducibility was also evaluated using F-scores which showed small variations between labs (Fig. 4 b), indicating the dependence of lab reproducibility on other factors (callers, aligners, and platforms). Observations in analysis of the F-scores between labs were consistent with the findings from analysis of variants between labs: lab reproducibility is higher for SNVs, especially for the upper bound, while indels are relatively less reproducible across labs.

### Aligner reproducibility

**Fig. 5:**
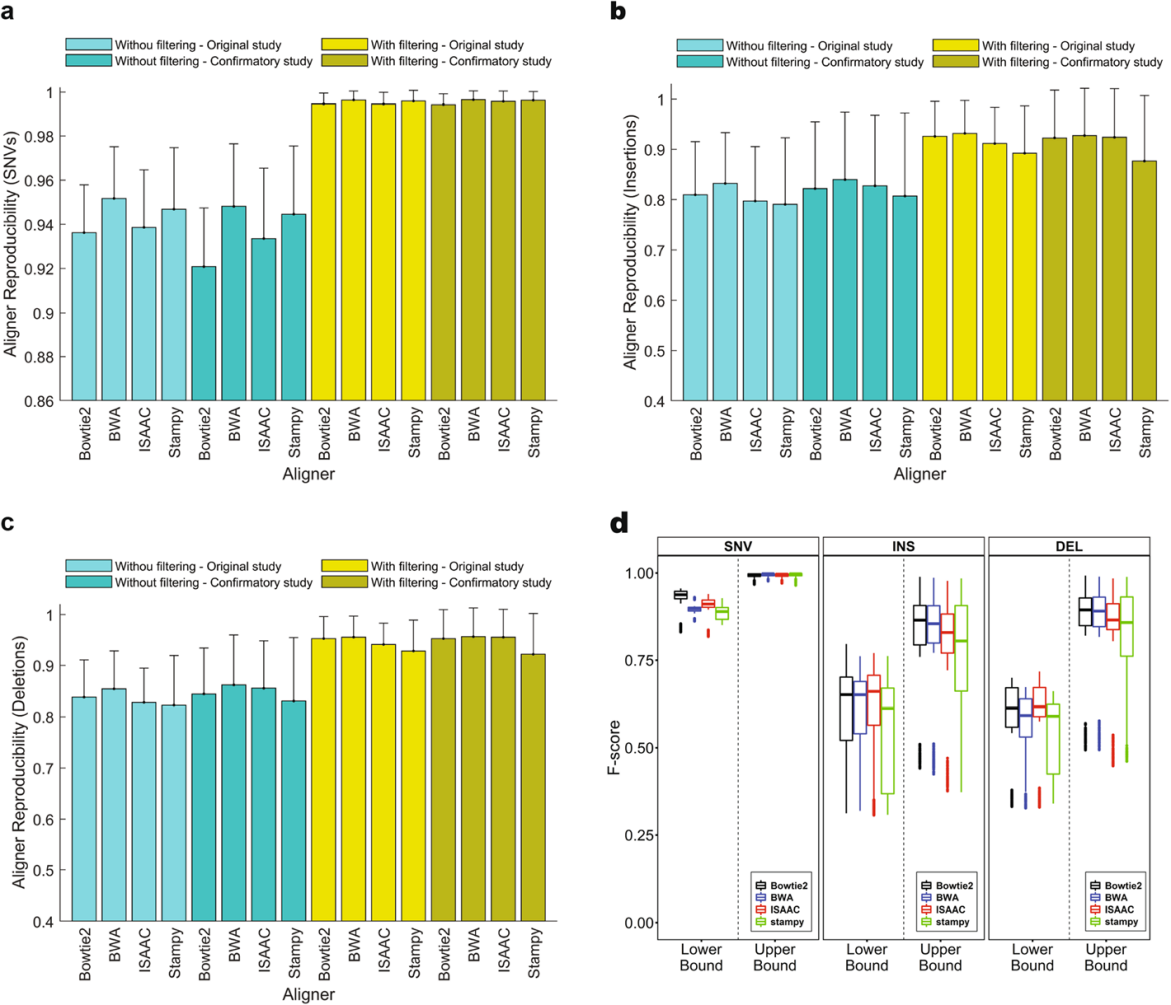
Aligner reproducibility. **a** Aligner reproducibility of SNVs. **b** Aligner reproducibility of insertions. **c** Aligner reproducibility of deletions. The bars represent average values of aligner reproducibility for the four aligners depicted by the *x*-axis ticks. The error sticks show standard deviation. The color legend specifies if variants were filtered by HRR or not as well as if the data are from original or confirmatory studies. **d** Boxplots of F-scores for SNVs (left panel), insertions (middle panel), and deletions (right panel). Results from the four aligners are plotted in different colors: black for Bowtie2, blue for BWA, red for ISAAC, and green for Stampy. F-scores from the lower bound and upper bound of variants are separated and marked at the *x*-axis

To ascertain causes of the considerable contributions of aligners (Bowtie2, BWA-MEM (shorten as BWA hereafter), ISAAC, and Stampy) to reproducibility, we calculated variant reproducibility between aligners holding other factors (sample, lab, and caller) constant (Additional file 14, Table S13). The lower bound of aligner reproducibility for SNVs from the original study varied among the aligners, but the upper bound increased from (0.936 to 0.952) to (0.994 to 0.998) (Fig. 5 a), demonstrating that SNVs in the HRR were reproducible among aligners. We examined the impact of other factors on aligner reproducibility. Aligner reproducibility appeared independent of DNA samples and sequencing labs but varied with the callers, especially for the variants before filtering to the HRR (Additional file 8: Fig. S23), consistent with the overall variance analysis. Careful examination of aligner reproducibility of the SNVs without filtering found that VarScan and FreeBayes were less reproducible between aligners (0.918 and 0.927, respectively), than ISSAC and GATK-HC (Haplotype caller) (0.967 and 0.961, respectively). However, SNVs in the HRR for all callers reached a high aligner reproducibility > 0.99 for small variations, indicating that the HRR are useful in identification of reproducible SNVs. Moreover, the observations on aligner reproducibility in the original study were replicated in the confirmatory study (Fig. 5 a).

The lower bound of insertion aligner reproducibility from the original study varied among the aligners (0.822 to 0.854), while the upper bound increased to 0.936 to 0.952 (Fig. 5 b), demonstrating usefulness of the HRR. DNA samples and sequencing labs did not show substantial differences in aligner reproducibility of insertions. However, callers had a large variation in aligner reproducibility, especially for the lower bound. Comparison between the callers found that Samtools had the lowest aligner reproducibility of insertions, while GATK-HC had the highest aligner reproducibility, possibly due to the local reassembly step in GATK-HC (Additional file 8: Fig. S24). The observations in the original study were confirmed in the confirmatory study.

The deletions had slightly higher aligner reproducibility than the insertions, but the patterns were similar to insertions (Fig. 5 c). Callers had the largest variation in aligner reproducibility and Samtools had the lowest aligner reproducibility. The patterns of aligner reproducibility for deletions observed in the original study repeated in the confirmatory study (Additional file 8: Fig. S25).

The F-scores between aligners were used to measure aligner reproducibility. The F-scores (Fig. 5 d) indicated that aligner reproducibility was higher for SNVs than for indels. Comparing the lab reproducibility using F-scores (Fig. 4 b) revealed that aligners were less reproducible than labs, indicating aligners caused a relatively larger variation in inherited variants than labs.

### Caller reproducibility

**Fig. 6:**
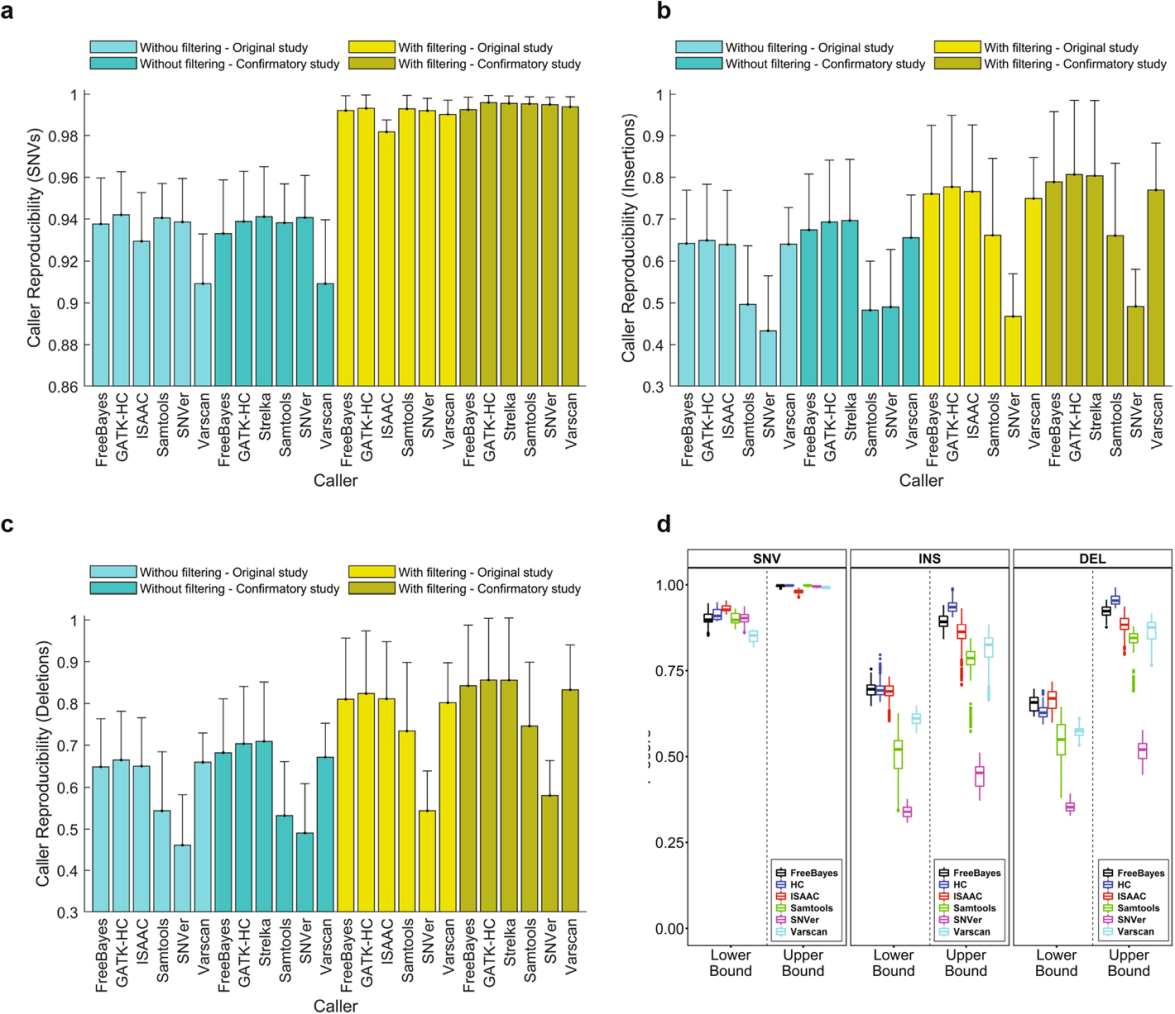
Caller reproducibility. **a** Caller reproducibility of SNVs. **b** Caller reproducibility of insertions. **c** Caller reproducibility of deletions. The bars represent average values of caller reproducibility for the six callers depicted at the *x*-axis ticks. The error sticks above the bars represent standard deviations. The color legend specifies if variants were filtered by HRR or not as well as data are from original or confirmatory studies. **d** Boxplots of F-scores for SNVs (left panel), insertions (middle panel), and deletions (right panel). Results from the six callers are plotted in different colors: black for FreeBayes, blue for HC, red for ISAAC, green for Samtools, magenta for SNVer, and cyan for VarScan. F-scores from the lower bound and upper bound of variants are separated and marked at the *x*-axis

We dissected caller reproducibility in detail to understand the causes of variation. Variants called using different callers were compared to calculate caller reproducibility (Additional file 15, Table S14). The lower bound of caller reproducibility in SNVs from the original study varied from 0.909 to 0.942, while the upper bound increased to 0.980 to 0.998 (Fig. 6 a). Interestingly, ISAAC in the original study showed a lower reproducibility than the updated version Strelka2 in the confirmatory study. Examining the impact of other factors found caller reproducibility was affected by aligners, but not by DNA samples and sequencing labs, especially for its lower bound. Compared to aligner reproducibility, caller reproducibility not only was lower but also had a larger variation, consistent with the overall variance analysis (Fig. 2). Bowtie2 and Stampy had a worse lower bound of caller reproducibility in SNVs, while the upper bounds were not substantially different, confirming that the HRR are useful in identification of reproducible SNVs (Additional file 8: Fig. S26). Moreover, the patterns in caller reproducibility in the original study were replicated in the confirmatory study.

Caller reproducibility of insertions (Fig. 6 b) and deletions (Fig. 6 c) not only were lower but also had a larger variation than aligner reproducibility, for both lower and upper bounds. Furthermore, other factors including DNA samples and sequencing labs did not show substantial differences in caller reproducibility for indels (Additional file 8: Fig. S27 and S28). Again, patterns in caller reproducibility for indels in the original study were replicated in the confirmatory study.

The F-scores had a larger variation among the callers (Fig. 6 d) compared with those among aligners, further confirming that callers were the major factor causing variation in reproducibility. The F-scores showed that caller reproducibility was higher for SNVs than for indels.

### GATK realignment effect

The old GATK best practices suggested realignment of reads near indels after initial alignment [35]. To evaluate the effect of GATK realignment, we generated variants with and without realignment and then compared their reproducibility. We found GATK realignment did not substantially improve SNV reproducibility across technical replicates (Additional file 8: Fig. S29-S32), labs (Additional file 8: Fig. S33 and S34), aligners (Additional file 8: Fig. S35-S38), and callers (Additional file 8: Fig. S39-S42), indicating benefit of GATK realignment on variant reproducibility is limited. Considering its computational cost, we recommend removal of realignment from variant calling, consistent with the new GATK best practices. Therefore, we did not include it in our confirmatory study.

## Discussion

Inherited variants underpin diseases ranging from rare diseases [36], autoimmune [37, 38], inflammatory conditions [39], developmental disorders [40, 41], and certain familial cancer types [42]. The urgent unmet clinical need in all these disease areas and more precipitates the need for rigorous and robust WGS inherited variant detection. While producing single-nucleotide-level genomic resolution of SNVs and indels, WGS variant calling can be impacted by a multitude of wet lab, sequencing, reference genome [43], and computational factors, as well as interactions of these factors. To fully understand and measure the impact of these factors and to improve the reproducibility of WGS variant calling, it is imperative to comprehensively analyze the relative importance of these sources of variability on inherited variant calling results. In order to understand sources of variation and which variants and regions are most reproducible, we defined the HRVs and HRR for each DNA sample. Furthermore, we performed a confirmatory study with different short-read WGS platforms, library preparations, and bioinformatics methods to replicate the findings in our original study. Consistent results imply that the observed reproducibility could be extendable to other short-read WGS-based methods.

By comparing the HRVs to the GIAB benchmark for NA12878, we found that the HRVs had a low FP rate. However, approximately 91% of SNVs in the benchmark were reproducible across all the short-read WGS methods we tested, but about 97% of SNVs were reproducible after excluding all difficult, repetitive regions defined by GA4GH. Most of the non-reproducible SNVs were in difficult to map regions. Approximately 30% of indels were not reproducible, particularly for indels > 5 bp. These results highlight limitations of short-read technology and the importance of optimizing bioinformatics in difficult to map regions and for large indels.

Short-read sequencing is notably limited for tandem repeats longer than the read length and segmental duplications [44, 45]. Thus, we excluded these regions in the HRR. Both sequencing technology and calling algorithms will need to improve to increase the reproducibility of variants in these regions in the future [46]. Development of long-read sequencing technology of ~ 1000 bp or more with higher base-call precision could be ideal for variant detection in such repetitive regions [47, 48]. Moreover, the observation of a large distance between the upper and lower bounds of reproducibility, especially for indels (Fig. 3 A) suggests caution when considering inherited variants detected outside of the HRR with new algorithms or technologies.

We systematically evaluated the impact of several factors in WGS inherited variant detection. Our results revealed that bioinformatics pipelines had the largest impact on variant calling reproducibility. Breaking bioinformatics up into components, the caller contributed more than the aligner to the variance in reproducibility. In contrast to the impact of bioinformatics, short-read sequencing experiment-related factors (labs, platforms, library preparations, DNA samples) had much smaller impacts, though they can still be important. Our findings suggest that selection of aligner and caller for inherited variants calling should be done carefully. The somatic mutations working group of SEQC2 also found that bioinformatics components were the largest source of variability in somatic mutations [49]. The specific ranked performance of the bioinformatics tools established here was solely based on reproducibility and thus may not be extrapolated accurately to other studies with focus on accuracy, sensitivity, or efficiency [46]. It is important to understand that reproducibility is no panacea. Even though our results did not show a specific aligner or caller constantly outperform others in reproducibility, we demonstrated that depending on variant types some aligners and callers performed worse than others. Therefore, when setting up bioinformatics pipeline for inherited variant calling from short-read WGS data, Strelka2 and HaplotypeCaller in GATK are recommended to ensure reproducibility. On the other hand, comprehensive comparisons of extensive WGS analyses for inherited variants are directly applicable to most mammalian species that have a comparable size to the human genome with a similar G/C content. Thus, our study should contribute not only to medical and clinical fields but also to fundamental genomic sciences, e.g., from evolutionary studies to disease model animal developments.

For every study, the question arises of how the tradeoff between sensitivity at the potential expense of reproducibility should be handled, depending on the study goal and the resources available. Furthermore, our study clearly highlights the necessity of standardized pipelines, especially for large projects, and the importance of harmonization approaches and continuous quality assessment over controls (e.g., UK-biobank). Nevertheless, it is also apparent from our study that challenges in SNV calling remain, such as the impact of alignment methods that cannot be resolved over a realignment step, or other factors carefully outlined here that lead to variabilities. Ideally, the SNV callers would in the future incorporate models that may make deduplication of reads and such unnecessary and further increase reproducibility and confidence in variation.

To confirm that our observations from the reproducibility analysis continue to be useful for new short-read sequencing platforms and analysis methods, we applied similar methods to the confirmatory sequencing data from new platform or library preparations (TruSeq without PCR and Nextera with PCR) based on our generated HRVs and HRR. We found most individual callers performed similarly as in the original data set. Only ISAAC and its updated version Strelka2 changed in performance. This observed improvement in the confirmatory data set is likely driven by algorithmic updates. Taken together, our results suggest an advantage of selecting a pipeline with continued support and development at the time of setting up the bioinformatics pipeline. As an interesting addendum, we also found the PCR-free TruSeq library had similar reproducibility to the PCR-based Nextera library.

We did not include filtering/variant recalibration for GATK-HC. It is worth to point out that variant recalibration is an important source of variability. However, this is a time-consuming variable to explore and requires well-curated training resources and is also not suitable for small-sample-size experiments such as this study. Though VariantFiltration tool in GATK can be used to hard-filter variants called from GATK when variant recalibration is hard to perform, such variant filtering (variant recalibration tool) is beyond the scope of this paper because we focused on reproducibility of variants without post-calling processing for all calling algorithms.

Despite our experimental design, the current study has several limitations. First, our analysis focused on SNVs and small indels. Other types of variants including structural variants, copy number variations, and tandem repeats were kept for future work. Our results were for short-read data and short-read variant callers; thus, our findings may not be extrapolated to long-read technologies and different calling algorithms. Nevertheless, we expect similar challenges, if not more, with the infant state of methods currently available for long reads and the constant updating of sequencing technologies [50]. In this study, we further followed the default/recommended parameters for short-read aligners and callers and their combinations. We focus on the impact of bioinformatics pipelines with their most commonly used default settings on inherited variants calling rather than to optimize and recommend specific parameters for bioinformatics pipelines or performing a comprehensive analysis of each caller itself, since bioinformatics pipelines evolve rapidly.

## Conclusions

In summary, we queried whether various factors in inherited variant detection with WGS including sequencing platform/lab/library, aligner, and caller contribute to variance in reproducibility. The performance of data sets of technical replicates from different sequencing labs, libraries, and bioinformatics components assessed in our study can be used as a reference supporting regulatory science and precision medicine research for the WGS research community. To enable the research community to leverage our work, we provided our raw data and code for defining the HRVs and HRR to the community for making their own tweaks to improve practices for inherited variant detection and to develop more reproducible bioinformatics pipelines.

## Methods

### Sequencing of HapMap trio by Illumina HiSeq2000

The Hapmap Trio DNA samples (NA10835, NA12248, and NA12249) were purchased from Coriell Cell Repositories (Camden, NJ). The concentration and quality were quantified using a NanoDrop 2000c. The OD260/280 ranged from 1.8 to 1.89. The original DNA samples were diluted to 50 ng/μL and 100 μL from each sample and were used for Illumina sequencing.

Genomic DNA was quantified prior to library construction using PicoGreen (Quant-iT™ PicoGreen® dsDNA Reagent, Invitrogen, Catalog #: P11496). Quants were read with Spectromax Gemini XPS (Molecular Devices).

Paired-end libraries were manually generated from 500 ng to 1 μg of gDNA using the Illumina TruSeq DNA Sample Preparation Kit (Catalog number FC-121-2001), based on the protocol in the TruSeq DNA PCR-Free Sample Preparation Guide. Pre-fragmentation gDNA cleanup was performed using paramagnetic sample purification beads (Agencourt® AMPure® XP reagents, Beckman Coulter). Samples were fragmented and libraries were size selected following fragmentation and end-repair using paramagnetic sample purification beads, targeting 300-bp inserts. Final libraries were quality controlled for size using a gel electrophoretic separation system and were quantified.

Following library quantitation, DNA libraries were denatured, diluted, and clustered onto v3 flow cells using the Illumina cBot™ system. cBot runs were performed based on the cBot User Guide, using the reagents provided in Illumina TruSeq Cluster Kit v3.

Clustered v3 flow cells were loaded onto HiSeq 2000 instruments and sequenced with 100 bp paired-end, non-indexed runs. All samples were sequenced on independent lanes. Sequencing runs were performed based on the HiSeq 2000 User Guide, using Illumina TruSeq SBS v3 Reagents. Illumina HiSeq Control Software (HCS) and real-time analysis (RTA) was used on HiSeq 2000 sequencing runs for real-time image analysis and base calling.

### Sequencing of Chinese quartet and NA12878 by Illumina XTen

Chinese Quartet reference materials were from four immortalized lymphoblastoid cell lines (LCLs) of a “Chinese Quartet” family including father, mother, and two monozygotic twin daughters. These family volunteers were from the Fudan Taizhou cohort, representing a typical Chinese ethnicity genetic background. Lymphoblastoid cell lines were immortalized from blood B cells using Epstein-Barr virus (EBV) transformation. This study was approved by the independent ethics committee at the School of Life Sciences of Fudan University. All volunteers provided written informed consent to participate in the study.

The LCLs were cultured in RPMI 1640 (Gibco Catalog No. 31870-082) supplemented with fetal bovine serum (Gibco 10091-148) to a final concentration of 10% by volume. Cells were maintained at 37 °C with 5% CO_2_ and were sub-cultured every 3 to 4 days. After six passages, a total of about 2 × 109 cells were used for DNA extraction. The collected cells were washed with PBS for twice before DNA extraction using Blood & Cell Culture DNA Maxi Kit (QIAGEN 13362). The extracted DNA samples were stocked in TE buffer (10 mM TRIS, 1 mM EDTA, pH 8.0).

The NA12878 DNA reference material (RM8398) was purchased from the National Institute of Standards and Technology (NIST).

WGS data for Chinese Quartet and RM8398 reference materials were generated from three sequencing labs (ARD: Annoroad, WUX: WuXi NextCODE, and NVG: NovoGene) using the Illumina XTen machine.

Libraries were prepared for whole genome sequencing using TruSeq DNA nano (Illumina catalog number 15041110) according to the manufacturer’s instructions. In total, 200 ng DNA was used for the TruSeq library preparations. All labs unified in-house fragmentation conditions using Covaris with a target size of 350 bp. All reference materials were prepared with three replicates in a single batch. The library concentrations were measured by the Qubit 3.0 fluorometer with the Quant-iT dsDNA HS Assay kit (Thermo Fisher Scientific, catalog number Q32854). The quality of all libraries was assessed using an Agilent 2100 Bioanalyzer or TapeStation instrument (Agilent).

These whole-genome libraries were sequenced on the Hiseq XTen (Illumina) with paired end 150 bp read length leveraging synthesis (SBS) chemistry. Sequencing was performed following the manufacturer’s instructions.

### Sequencing of HapMap trio and Chinese quartet by Illumina NovoSeq with library preparation kit Nextera

Samples were quantified for dsDNA content with the Qubit dsDNA HS assay kit. Out of 21 samples, two contained less than 100 ng DNA. For samples with sufficient DNA, 100 ng was used as input for the Illumina Nextera DNA Flex library preparation kit (Illumina, catalog number 20018704). Libraries were prepared according to the manufacturer’s instructions (Illumina, Nextera DNA Flex Library Prep Reference Guide), with five PCR cycles used for amplification. For the two lower input samples, one sample had 62 ng DNA input and was prepared the same as the 100-ng samples. The other sample had 17 ng DNA input, so the PCR cycle number was increased to nine cycles, which resulted in shorter insert sizes in the final library for this sample.

Library yield and fragment size were quantified using the Qubit dsDNA HS assay kit and Agilent 2100 Bioanalyzer HS DNA chip, respectively. Libraries were loaded onto two NovaSeq S4 flow cells and clustered according to manufacturer’s instructions. Run data sets were uploaded to BaseSpace, and fastq files were generated.

### Sequencing of HapMap Trio and Chinese quartet by Illumina NovaSeq with library preparation kit TrueSeq

Libraries were prepared using the TruSeq DNA PCR-Free Library Prep Kit (Illumina, catalog number 20015962) with a modified protocol to target 450 bp insert using 600 ng input. Shearing was performed with Covaris LE220 (18% Duty factor, 450 PIP (W), 200 Cycles/Burst, 60 s, 4 to 8.5 °C) and SPRI dilution to remove large DNA fragments (88 μL SPB + 72 μL Water), and IDT for Illumina Unique Dual Indexes (Illumina, catalog number 20020178). Sequencing was performed on the Illumina NovaSeq6000 Sequencing System with Xp loading on an S4 flowcell and 151 × 8 × 8 × 151 cycles. Raw run data were streamed onto the BaseSpace Sequence Hub from the sequencer. Fastq files were generated using bcl2fastq on the BaseSpace Sequence hub with default parameters. Adapters were trimmed during fastq generation using AGATCGGAAGAGCACACGTCTGAACTCCAGTCA as the read 1 adapter and AGATCGGAAGAGCGTCGTGTAGGGAAAGAGTGT as the read 2 adapter. To confirm quality and coverage of samples, fastqs were processed through the Whole Genome Sequencing v8.0.1 BaseSpace app, with alignment against the GRCh38Decoy reference genome with default parameters. Down sampling to 100× coverage was enabled in the Whole Genome Sequencing app for any sample with coverage beyond 100×. Two samples in the original NovaSeq 6000 S4 run did not reach 60× average autosomal coverage and were re-sequenced on an S1 flowcell. Re-sequenced samples were analyzed in the same manner, and we confirmed greater than 60× coverage.

### Quality assessment of sequencing data

All fastq files were evaluated with FastQC [51] (v0.11.5) with default setting for assessment of base quality, adapter content, and so on. Per base sequence quality was extracted with shell script from the “fastqc_data.txt” file reported by FastQC to check if the data quality passed or not.

### Sequence reads alignment

The short reads were first aligned to the latest human reference genome [43] (GRCh38 with decoy sequences downloaded from Genomic data commons of the National Cancer Institute) using four aligners: Bowtie2 (v2.2.9) [52], BWA [53] (v0.7.15), ISAAC [54] (v1.0.7), and Stampy [55] (v1.0.29). Default settings for Bowtie2 and BWA were applied. BWA was used as a pre-aligner for Stampy, which was suggested by Stampy’s developer for efficient alignment. Stampy’s default settings were used except for application of the “--bamkeepgoodreads”. The setting “--base-calls-format --stop-at Bam --keep-unaligned back --realign-gaps yes” was used in ISAAC alignment to get sorted BAM files. Resulting SAM files from the other three aligners were sorted and converted to BAM files by the SortSam module in Picard [56] (v2.7.1). Duplicates in the sorted BAM files were marked by module MarkDuplicates and read groups were assigned by module AddOrReplaceReadGroups in Picard (v2.7.1).

### GATK realignment

All BAM files obtained from alignment in the original study were processed with GATK [35] realignment following the best practices recommended by the Broad Institute (Notice: the realignment recommendation was removed by the Broad Institute beginning with GATK v4.0). Each BAM file was processed with local-realignment by GATK modules RealignerTargetCreator and IndelRealigner and base-quality recalibration by GATK modules BaseRecalibrator and PrintReads by following the best practices from the Broad Institute. The known SNPs and indels for GRCh38 in DBsnp146 (ftp://gsapubftp-anonymous@ftp.broadinstitute.org/bundle/hg38/dbsnp_146.hg38.vcf.gz) and two indel files (ftp://ftp.1000genomes.ebi.ac.uk/vol1/ftp/technical/reference/GRCh38_reference_genome/other_mapping_resources/Mills_and_1000G_gold_standard.indels.b38.primary_assembly.vcf.gz) and (ftp://ftp.1000genomes.ebi.ac.uk/vol1/ftp/technical/reference/GRCh38_reference_genome/other_mapping_resources/ALL.wgs.1000G_phase3.GRCh38.ncbi_remapper.20150424.shapeit2_indels.vcf.gz) were used as reference in the realignment and recalibration process.

### Variant calling

BAM files with and without GATK realignment were used for variant calling using six different callers: FreeBayes [57] (v1.1.0), GATK-HaplotypeCaller [35] (v3.7), ISAAC [54] (v 1.0.7), Samtools [58] (v1.3.1), SNVer [59] (v0.5.3), and VarScan [60] (version 2.3.9). The running options “-X -0 -u -v” in FreeBayes, “-rf BadCigar –dbsnp dbsnp_146.hg38.VCF --stand_call_conf 30” in GATK-HaplotypeCaller, “minMapq = 20; minGQX = 30” in ISAAC, “-ugf” and “-vmO” from bcftools (v1.3.1) in Samtools, “-p 0.05” in SNVer, and “-p 0.05 --min-coverage 8 --min-reads2 2 --p-value 0.05” in VarScan were used in variant calling. Variant calling results were stored in VCF format.

### Variant calling by Sentieon

The decoy version of GRCh38 human reference genome (https://gdc.cancer.gov/about-data/data-harmonization-and-generation/gdc-reference -files; GRCh38.d1.dv1.fa) from the Genomic Data Commons (GDC) was used in variant calling by Sentieon (v201711.02). The Sentieon DNAseq pipeline is a tool for variant calling from raw Fastq files, including read mapping by BWA, duplicate removal, indel realignment, base quality score re-calibration, and variant calling by Haplotyper. The Sentieon DNAseq pipeline, Sentieon [61] v201711.03, provides a complete rewrite of the mathematical models of the GATK Best Practices with a focus on computational efficiency, accuracy, and consistency. In variant calling by Sentieon, sequence reads were first aligned to GRCh38.d1.vd1.fa with Sentieon BWA, followed by sorting and indexing with the Sentieon utility. Subsequently, duplicate reads were removed, and base qualities were recalibrated with the Sentieon driver program. Variant calling was then performed with Sentieon Haplotype caller.

### Variant calling by Dragen

Reads were aligned to human genome reference GRCh38 in BaseSpace using DRAGEN Germline Pipeline version 3.2.8 in Whole Genome Sequencing v7.7.0 (WGSv7). Although the DRAGEN aligner was able to use all reads for alignment, WGSv7 requires fewer than 1 billion paired end reads for analysis. Therefore, fastq files were down-sampled to 990 million paired-end reads in BaseSpace using FASTQ Toolkit v2.2.0 to enable WGSv7 analysis. Variant calling was performed in BaseSpace using DRAGEN Germline Pipeline version 3.2.8. The DRAGEN pipeline was run with default settings with “Map/Align + Variant Caller” selected, and CNV calling, SV calling, and duplicate marking enabled.

### Variant calling by RTG

The following describes the processing used to align reads and call variants using RTG [62] alignment and variant calling algorithms.

All FASTQs underwent quality-based filtering to trim off poor quality read ends (using “rtg fastqtrim” --end-quality-threshold 15) and formatting to the RTG SDF format (which allows random access to arbitrary chunks of reads during mapping) using “rtg format”. FASTQ file pairs for each replicate were merged to a single per-replicate SDF and assigned a unique read group.

Alignment of the reads for each sample to the reference genome GRCh38 was via “rtg map,” processing reads from the input SDF file in chunks to permit partitioning of the alignment across multiple nodes. A typical chunk size was 40 million read-pairs. During alignment, an appropriate pedigree file was supplied to the mapping command to allow the aligner to lookup the sex of the sample. After primary alignment, an additional mate-pair rescue tool (currently in development) was executed on any reads which were unmapped but for which the other arm of the pair was uniquely mapped, and any rescued alignments were included in subsequent variant calling.

Across the various samples and families in the SEQC2 project, several variant calling modes were employed. When calling a single sample in isolation, the “rtg snp” command was used, for example: rtg snp -t GRCh38.d1.vd1.sdf \ -T 8 --pedigree pedigree.ped \ --enable-allelic-fraction --XXcom.rtg.variant.mask-homopolymer=true \ -o snp_HG001-r1-H3WNJDSXX_S8 \ map_HG001-r1-H3WNJDSXX_S8.sdf_*/alignments.bam. The final argument supplies all the alignment BAMs corresponding to the particular sample.

Analysis of Mendelian inheritance errors were computed using “rtg mendelian.” Overall variant statistics for each sample were computed using “rtg vcfstats.”

### Variance analysis

Variants concordance was calculated using the average Jaccard Index as follows: ((A∩B)/(A∪B) + (A∩C)/(A∪C) + (B∩C)/(B∪C))/3, where A, B, and C are variants from the three replicates of each sample. Contribution of four factors (caller, aligner, platform, and sample) to the variation in concordances was estimated by a non-linear Gradient Boosted Tree (JMP Pro v14.3). The importance of each factor was estimated by how often it is used to make key decisions with decision trees. Boosting is the process of building a large, additive decision tree by fitting a sequence of smaller decision trees, called layers. The tree at each layer consists of a small number of splits. The tree is fit based on the residuals of the previous layers, which allows each layer to correct the fit for poorly fitting data from the previous layers. The final prediction for an observation is the sum of the predictions for that observation over all of the layers. The factor contributions were estimated in the model fitting, which is based on the total number of instances over all of the trees when the specific factor is used to split the data. The proportion of the contribution of each factor was calculated as sum of squares attributed to the factor divided by the total sum of squares.

In addition to the non-linear method, we also estimated the contribution of all possible 2-way interactions of the factors in a Variance Components Analysis (JMP Pro v14.3). The variance components were parameterized using an unrestricted method [63] in a mixed model fitted with restricted maximum likelihood (REML). Student’s *t* test was used to assess the contribution difference between factors. Variance components were estimated through fitting a random effect model as follows:

$$ Y= Z\gamma +\varepsilon, $$

$$ \gamma \sim N\left(0,G\right) $$

$$ \varepsilon \sim N\left(0,{\sigma}^2{I}_n\right) $$

where *Y* denotes an *n* × 1 vector of response (Jaccard index), *Z* is the design matrix for the random effects, and *γ* is a vector of unknown random effects with design matrix *Z*. Both *γ* and *ε* are assumed following normal distribution with means at 0. *G* and *σ*^2^ are the variance components that need to be estimated. The ratio of the contribution of each factor to the overall variability was calculated by the variance component of each factor divided by the total.

### Generation of highly reproducible variants (HRV) and highly reproducible regions (HRR)

We generated HRVs and defined HRRs to assess the upper bound of reproducibility based on the suggestion from GIAB4. We used the workflow shown in Additional file 8: Fig. S1 to generate the HRRs. Detailed procedures for defining the HRRs are given below.

#### Determining mappable regions

Each of the BAM files from all aligners (with and without GATK realignment), replicates, and labs was used to generate a region file having aligned reads using GATK-CallableLoci with cutoffs of minimum depth of 6, maximum depth of 160, minimum mapping quality of 10, and minimum base mapping quality of 20%. We created 81 and 27 region files for each Chinese Quartet sample and HapMap sample (including NA12878), respectively. The mappable regions for each sample were determined as the genome regions that were covered by any of the region files of the sample. Technically, the mappable regions for a sample are the union of the region files.

#### Identify consensus mappable regions

The determined mappable regions for a sample were covered by a different number of region files. To identify the consensus mappable regions of the sample, its determined mappable regions were ranked by the number of region files that cover the regions. The top 99% ranked determined mappable regions were elected as the consensus mappable regions. The resulting consensus mappable regions are the regions covered by ≥ 10 region files for CQ-5, CQ-6, and CQ-7, by ≥ 11 region files for CQ-8, by ≥ 9 for NA12878, and ≥ 3 for all three HapMap samples.

#### Determine callable regions

Some genomic regions present variant calling difficulties and were removed from the identified consensus mappable regions. Specifically, simple repeats including homopolymer regions and super duplications defined in “SimpleRepeat_imperfecthomopolgt10_slop5.bed” and “remapped_superdupsmerged_all_sort.bed” by GIAB and GA4GH were removed using the subtract command from bedtools. The remaining consensus mappable regions were determined to be callable regions.

#### Define HRVs

First, the variants called from the same pipelines for three replicates of the same sample were compared and the variants called in only one replicate were filtered out as discordant variants. The remaining variants were used as replicate-concordant variants for the sample. Comparing the replicate-concordant variants from the three labs for the Chinese Quartet samples further filtered discordant variants that were found in the replicate-concordant variants of only one lab. The replicate-concordant variants of the HapMap samples and NA12878 as well as the post-filter replicate-concordant variants of the Chinese Quartet samples were then compared among aligners to determine aligner-concordant variants by filtering discordant variants that were identified in the replicate-concordant variants from only one aligner. The aligner-concordant variants were further compared among callers by filtering discordant variants that were shared by six or less callers. The remaining aligner-variants were determined as caller-concordant variants. The caller-concordant variants for NA12878 were defined as HRVs. The caller-concordant variants for the twins of Chinese Quartet were compared to filter discordant variants between the twins and the remaining caller-concordant variants were used for Mendelian rule compliance checking together with the call-concordant variants of the parent samples of Chinese Quartet and the HapMap trio samples. Variants violating the Mendelian rule were filtered out as discordant variants and the remaining Mendelian rule compliant variants were defined as HRVs.

#### Defining HRRs

For each sample, its HRVs and all discordant variants were used to filter the callable regions. For each of the discordant variants, the genome region 50 bp to its left and 50 bp to its right was compared with HRVs. If no HRV was located in this region, this region was removed from the callable region. When this region had HRVs, half of the region between the discordant variant and the nearest HRV were removed. After removal of such regions for all discordant variants, the remaining callable regions were defined as the HRR of the sample.

### Filtering variants from different pipelines

Different callers report variants with different minimum read depths. We applied depth filtering prior to lower bound reproducibility calculation so that the variants in reproducibility calculation have the same minimum read depth. We also filtered variants with very high read depth using a cutoff of mean read depth plus three times standard deviation of the read depth of all variants. Specifically, we used a minimum read depth of 8 for all samples and a maximum read depth of 223 for the Chinese quartet samples and NA12878 and a maximum read depth of 350 for HapMap trio samples. To assess the upper bound of reproducibility, we selected variants only in HRR. Specifically, the vcffilter command of RTG tool was used to filter the variants outside HRR.

### Reproducibility calculation

We calculated four types of reproducibility: technical reproducibility, lab reproducibility, aligner reproducibility, and caller reproducibility.

A reproducibility value was calculated between two sets a (*N*^*a*^ variants) and b (*N*^*b*^ variants) using eval command of RTG Tools (v3.9). First, we indexed the reference genome to sdf format with RTG’s format command. Then we took set a as querying and set b as baseline for the calculation. Four sets of variants were output from the calculation: unique variants in a, variants of a found in b (*n*^*a*^), unique variants in b, and variants of b found in a (*n*^*b*^). Basic information such as variant type and number was counted by RTG’s stat command. All numbers were extracted with a shell script written to extract the numbers and to calculate reproducibility R using equation (1).

1 $$ R=\frac{1}{2}\left(\frac{n^a}{N^a}+\frac{n^b}{N^b}\right) $$

### Calculation of precision, recall, and F-score

Precision, recall, and F-score were calculated by comparing a set of variants with its corresponding HRVs using equations (2-4). The comparison was done using RTG’s eval command.

2 $$ \mathrm{Precision}=\frac{Q^c}{Q^c+{Q}^u} $$

3 $$ \mathrm{Recall}=\frac{Q^c}{Q^c+{H}^u} $$

4 $$ {\mathrm{F}}_{\mathrm{score}}=2\ast \mathrm{Precsion}\ast \frac{\mathrm{Recall}}{\mathrm{Precision}+\mathrm{Recall}} $$

where *Q*^*c*^ is number of common variants; *Q*^*u*^ is number of variants in the comparing set but not in the HRVs; and *H*^*u*^ is number of variants in the HRVs but not in the comparing set.

## Supplementary Information

**Additional file 1: Table S1.** Detail information for WGS data generated.

**Additional file 2: Table S2.** Calling pipelines used.

**Additional file 3: Table S3.** SNV number.

**Additional file 4: Table S4.** Insertion number.

**Additional file 5: Table S5.** Deletion number.

**Additional file 6: Table S6.** Highly reproducible variants.

**Additional file 7: Table S7.** Highly reproducible beds.

**Additional file 8: Supplementary figures Fig. S1-S42.**

**Additional file 9: Table S8.** Comparison statistics to GIAB truth v4.0.

**Additional file 10: Table S9.** Factor contribution analysis by boosted tree.

**Additional file 11: Table S10.** Factor contribution analysis by variance component analysis.

**Additional file 12: Table S11.** Summary of technical reproducibility.

**Additional file 13: Table S12.** Summary of lab reproducibility.

**Additional file 14: Table S13.** Summary of aligner reproducibility

**Additional file 15: Table S14.** Summary of caller reproducibility.

## Data Availability

All raw read data (FASTQ files) are available in the National Omics Data Encyclopedia (NODE) database (https://www.biosino.org/node) and the SRA database (https://www.ncbi.nlm.nih.gov/sra). The Chinese Quartet data are available in NODE with the accession number OEP001896 [64]. The HapMap Trio data are available in SRA with the accession number PRJNA723125 [65]. High reproducible sets are available at Zenodo (https://zenodo.org/record/5275189#.YaaYn9DMJPZ) [66]. All codes used in processing the WGS data and reproducibility calculation are available at Github (https://github.com/justwalking2017/SEQC_WG3_Script) [67].
