# Supplementary material for: Assessing reproducibility of inherited variants detected with short-read whole genome sequencing
Source: Genome Biol. 2022 Jan 3;23:2. doi: 10.1186/s13059-021-02569-8 (PMC8722114; doi:10.1186/s13059-021-02569-8)
Supplement: Supplementary file 8 — Additional file 8: Supplementary figures Fig. S1-S42. [file 13059_2021_2569_MOESM8_ESM.pdf]

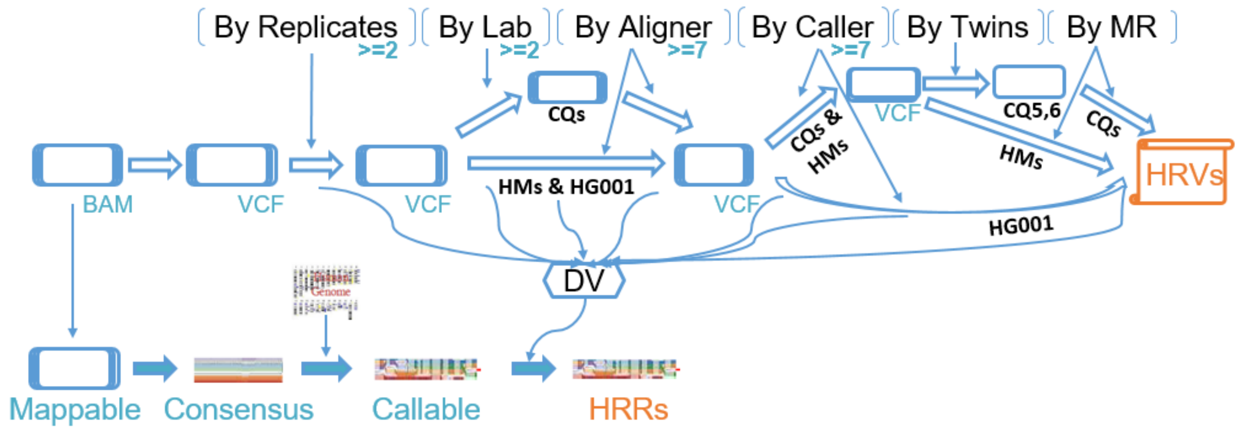

Fig. S1. Workflow for defining highly reproducible variants (HRVs) and highly reproducible regions (HRRs). The consensus progress consists of two parts: call set and genome region. The BAM files obtained from mapping were used to generate mappable regions. All mappable regions for a sample were analyzed to identify the consensus genome regions which was shared by most of the mappable regions. The difficult genome regions defined in the GIAB project were filtered from the consensus regions and the resulting regions were termed as "callable regions" for the sample. The callable regions were used to filter all call sets. Based on the filtered call sets, HRVs were determined by removing discordant variants among the replicates, labs, aligners, and callers. The variants not concordant between the twin samples and/or violating Mendelian rule were also identified as discordant variants and were removed. The resulting variants were reproducible among most of the replicates, labs, aligners and callers and compliant with Mendelian rule and were defined as HRVs. The discordant variants were used to filter the callable regions, removing the genome regions around the discordant variants (with a 50 bp extension to both left and right sides) if no HRVs existed in such regions. Otherwise, half of the extended regions were removed. The resulting callable regions were defined as HRRs for the sample.

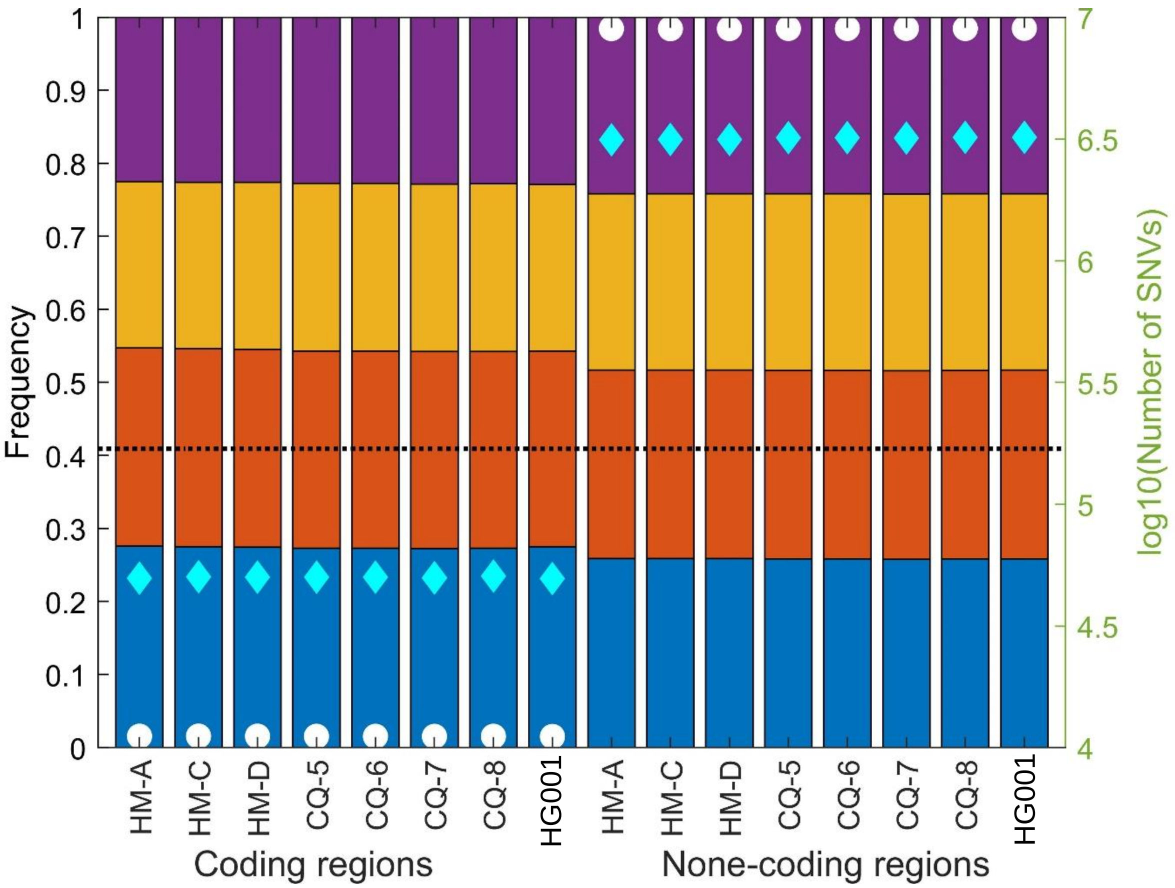

Fig. S2. Highly reproducible SNVs in the samples (x-axis ticks). Four-letter codes are used for samples (HM-A: NA10835; HM-C: NA12248; HM-D: NA12249; HG001: GIAB reference sample NA12878; CQ-5 and CQ-6: Chinese quartet twin daughters; CQ-7: Chinese quartet father; CQ8: Chinese mother). The left eight and right eight stacked bars show the base composition (left y-axis) of the highly reproducible SNVs in the coding regions and non-coding regions (x-axis label), respectively. The four bases are color coded (blue for G, red for C, yellow for A, and purple for T). The black dashed line represents the frequency of G and C in the reference genome. The solid white circles show the percentage of SNVs (left y-axis) in the coding region (1.52-1.57%) and non-coding region (98.43-98.48%). The solid cyan diamonds depict the number of SNVs (right y-axis in log10 unit) in the coding region (49516-50588) and non-coding region (3,133175-3209889).

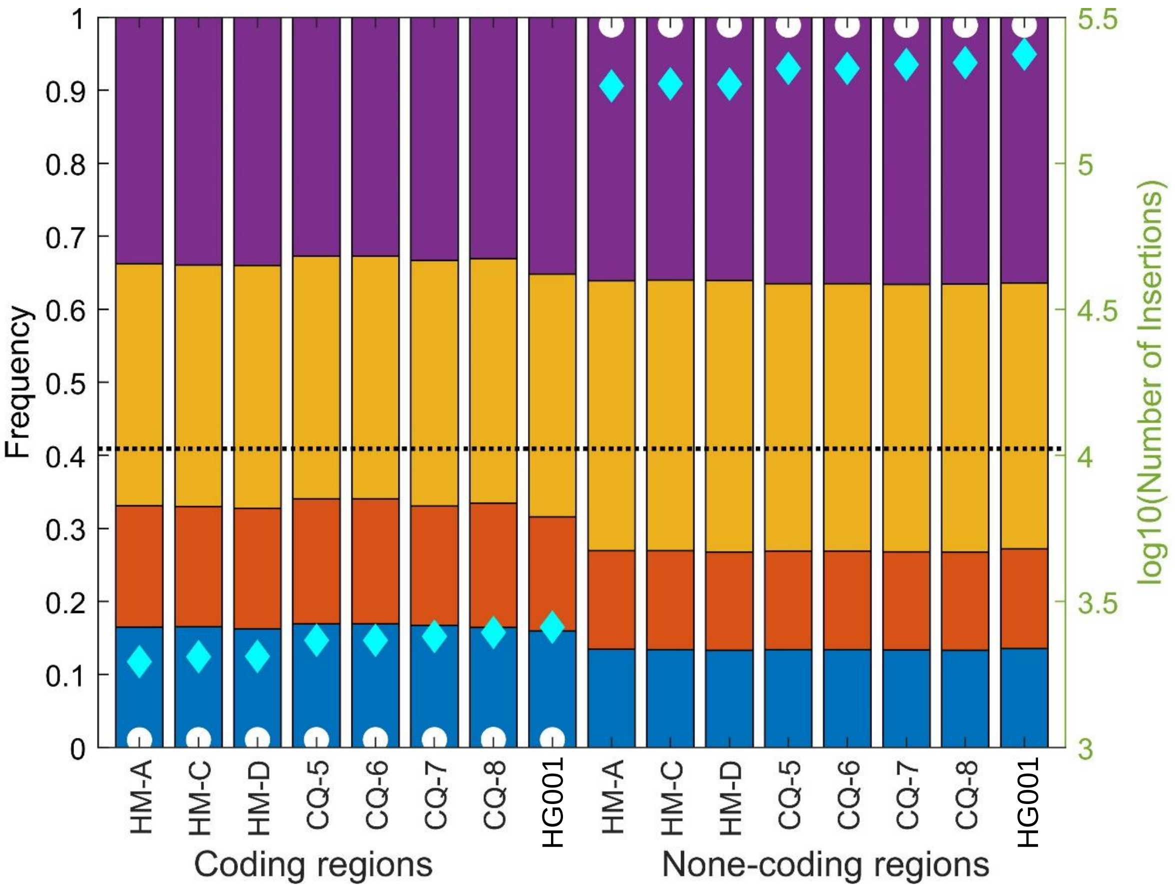

Fig. S3. Highly reproducible insertions in the samples (x-axis ticks). Four-letter codes are used for samples (HM-A: NA10835; HM-C: NA12248; HM-D: NA12249; HG001: GIAB reference sample NA12878; CQ-5 and CQ-6: Chinese quartet twin daughters; CQ-7: Chinese quartet father; CQ8: Chinese mother). The left eight and right eight stacked bars show the base composition (left y-axis) of the highly reproducible insertions in the coding regions and non-coding regions (x-axis label), respectively. The four bases are color coded (blue for G, red for C, yellow for A, and purple for T). The black dashed line represents the frequency of G and C in the reference genome. The solid white circles show the percentage of insertions (left y-axis) in the coding region (1.06-1.11%) and non-coding region (98.89-98.94%). The solid cyan diamonds depict the number of insertions (right y-axis, in log10 unit) in the coding region (1967-2586) and non-coding region (183772-236155).

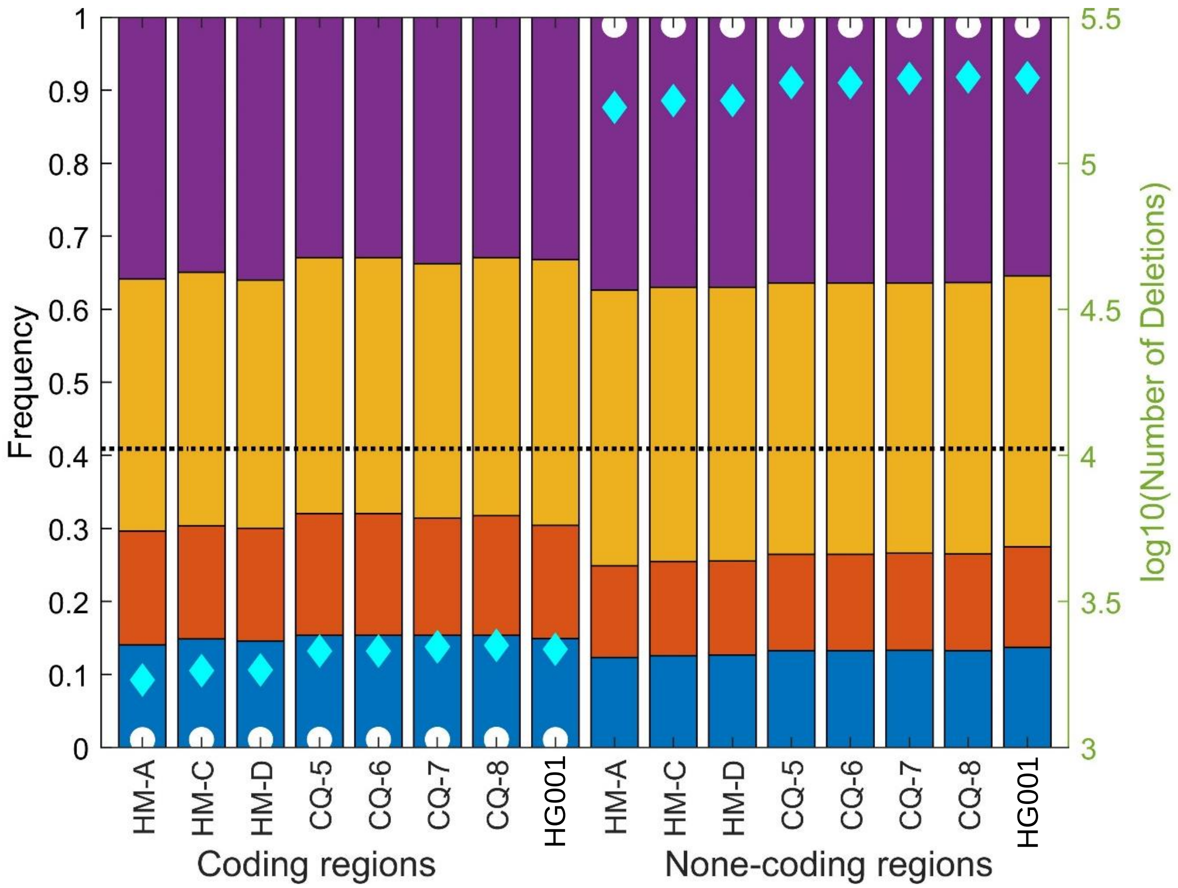

Fig. S4. Highly reproducible deletions in the samples (x-axis ticks). Four-letter codes are used for samples (HM-A: NA10835; HM-C: NA12248; HM-D: NA12249; HG001: GIAB reference sample NA12878; CQ-5 and CQ-6: Chinese quartet twin daughters; CQ-7: Chinese quartet father; CQ8: Chinese mother). The left eight and right eight stacked bars show the base composition (left y-axis) of the highly reproducible deletions in the coding regions and non-coding regions (x-axis label), respectively. The four bases are color coded (blue for G, red for C, yellow for A, and purple for T). The black dashed line represents the frequency of G and C in the reference genome. The solid white circles show the percentage of deletions (left y-axis) in the coding region (1.09-1.12%) and none-coding region (98.88-98.91%). The solid cyan diamonds depict the number of deletions (right y-axis, in log10 unit) in the coding region (1706-2242) and non-coding region (155255-197150).

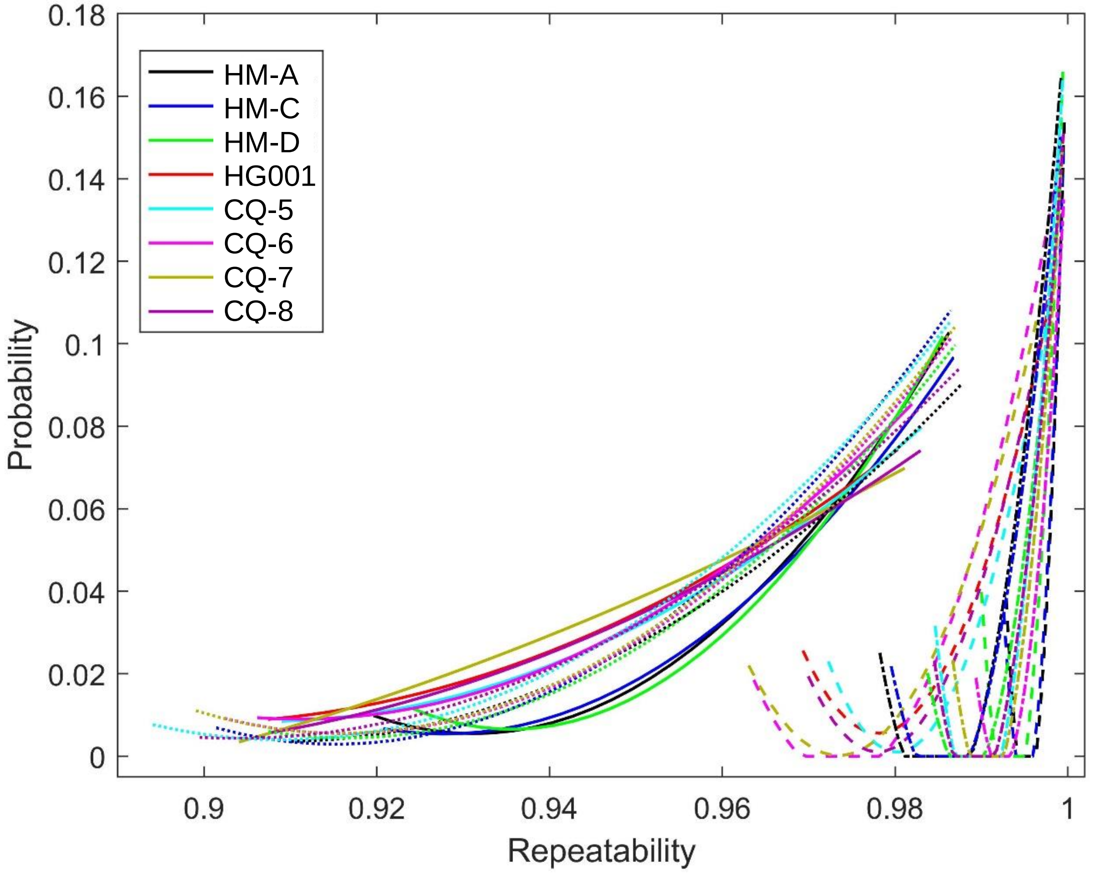

Fig. S5. Technical repeatability of SNVs. Samples are color coded as shown in the top left corner. Four-letter codes are used for samples (HM-A: NA10835; HM-C: NA12248; HM-D: NA12249; HG001: GIAB reference sample NA12878; CQ-5 and CQ-6: Chinese quartet twin daughters; CQ-7: Chinese quartet father; CQ8: Chinese mother). The repeatability distributions from the call sets without or with bed file filtering in the original study are plotted with solid and dashed curves respectively, while the repeatability distributions from the call sets without or with bed file filtering in the confirmatory study are plotted with dotted and dash-dot curves respectively.

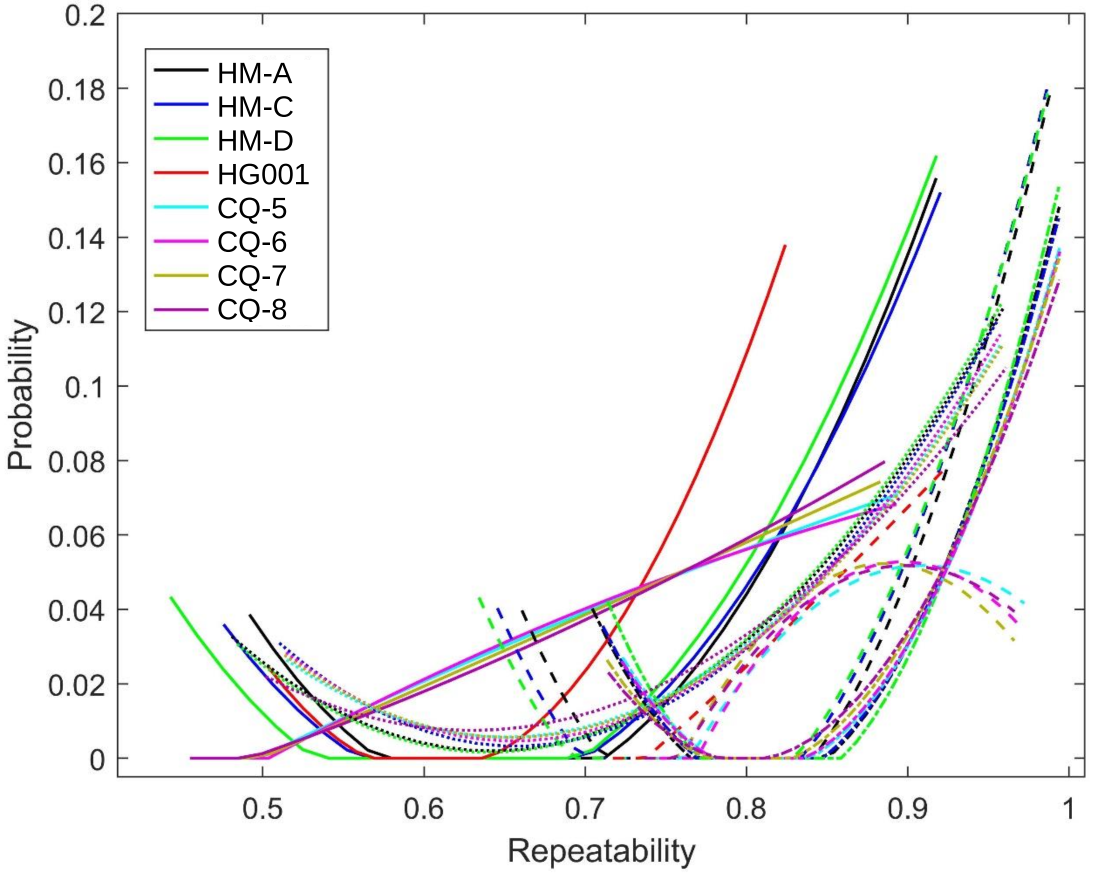

Fig. S6. Technical repeatability of insertions. Samples are color coded as shown in the top left corner. Four-letter codes are used for samples (HM-A: NA10835; HM-C: NA12248; HM-D: NA12249; HG001: GIAB reference sample NA12878; CQ-5 and CQ-6: Chinese quartet twin daughters; CQ-7: Chinese quartet father; CQ8: Chinese mother). The repeatability distributions from the call sets without or with bed file filtering in the original study are plotted with solid and dashed curves respectively, while the repeatability distributions from the call sets without or with bed file filtering in the confirmatory study are plotted with dotted and dash-dot curves respectively.

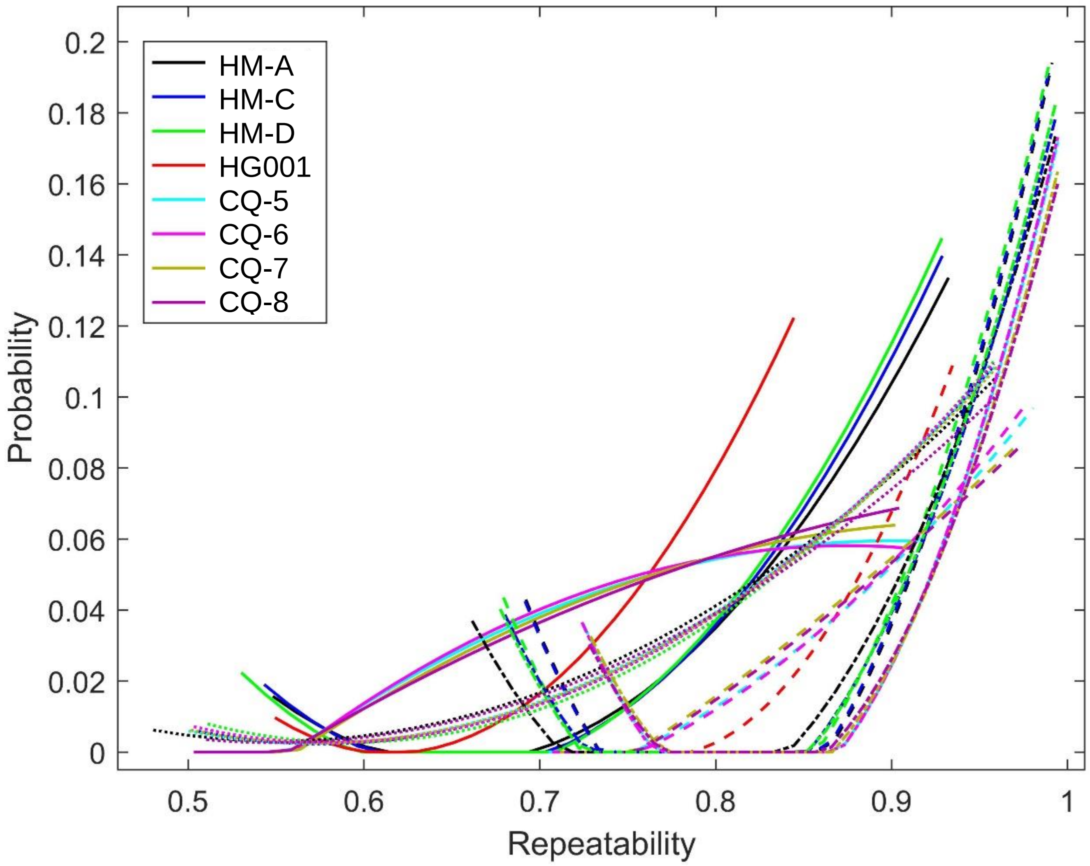

Fig. S7. Technical repeatability of deletions. Samples are color coded as shown in the top left corner. Four-letter codes are used for samples (HM-A: NA10835; HM-C: NA12248; HM-D: NA12249; HG001: GIAB reference sample NA12878; CQ-5 and CQ-6: Chinese quartet twin daughters; CQ-7: Chinese quartet father; CQ8: Chinese mother). The repeatability distributions from the call sets without or with bed file filtering in the original study are plotted with solid and dashed curves respectively, while the repeatability distributions from the call sets without or with bed file filtering in the confirmatory study are plotted with dotted and dash-dot curves respectively.

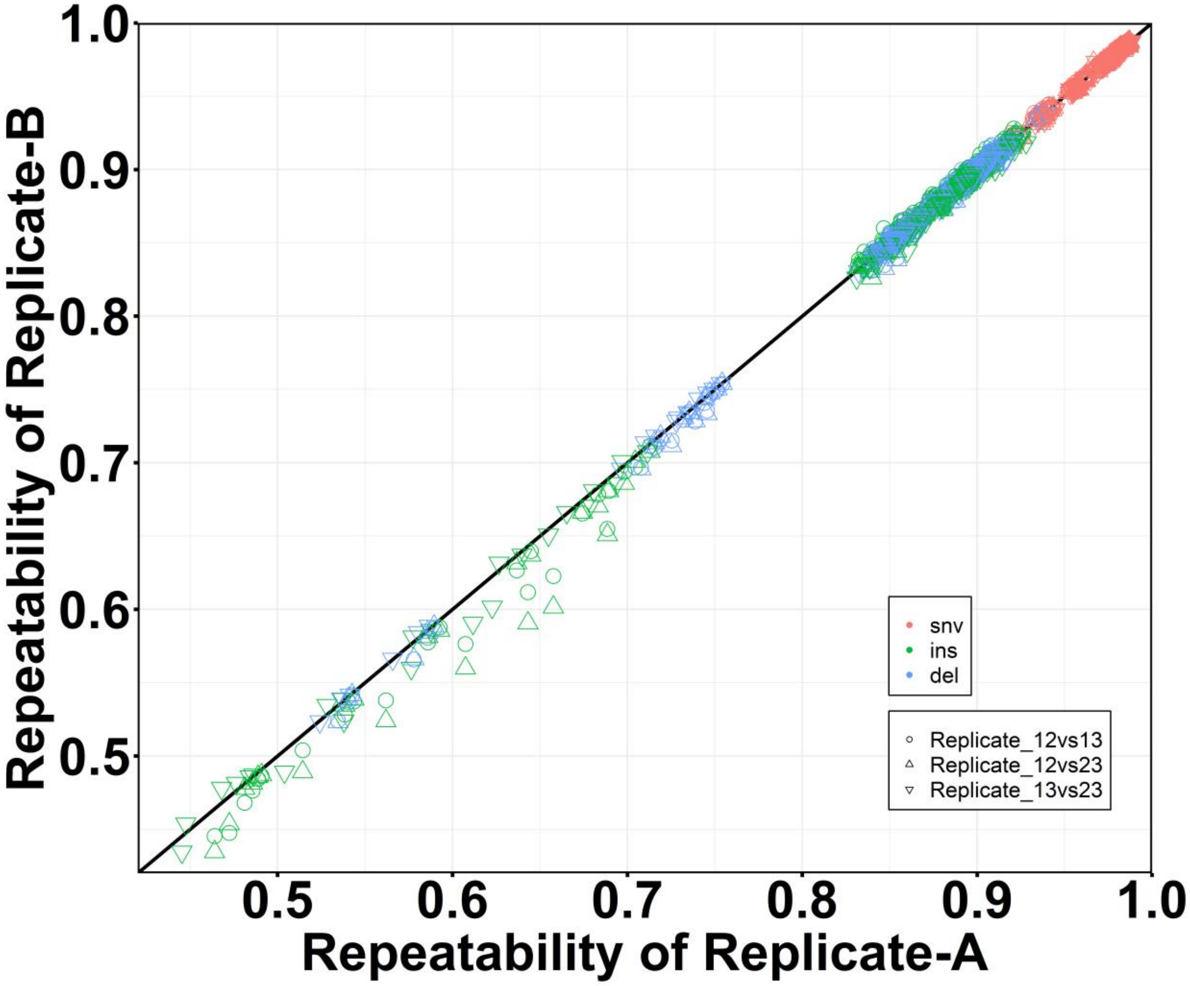

Fig. S8. Scatter plots of repeatability of replicate pairs of original HapMap sample data without filtering by HRRs. The points represent repeatability of two replicate pairs. Three types of variants are colored in three different colors: red for SNV, green for Insertion and blue for Deletion. The x-axis depicts repeatability calculated from one replicate pair, while the y-axis represents repeatability from the other replicate pair. Three comparison pairs presented in different shapes: circle for comparison between replicate pairs 1&2 and 1&3; upward triangles for replicate pairs 1&2 and 2&3; downward triangles for replicate pairs 1&3 and 2&3.

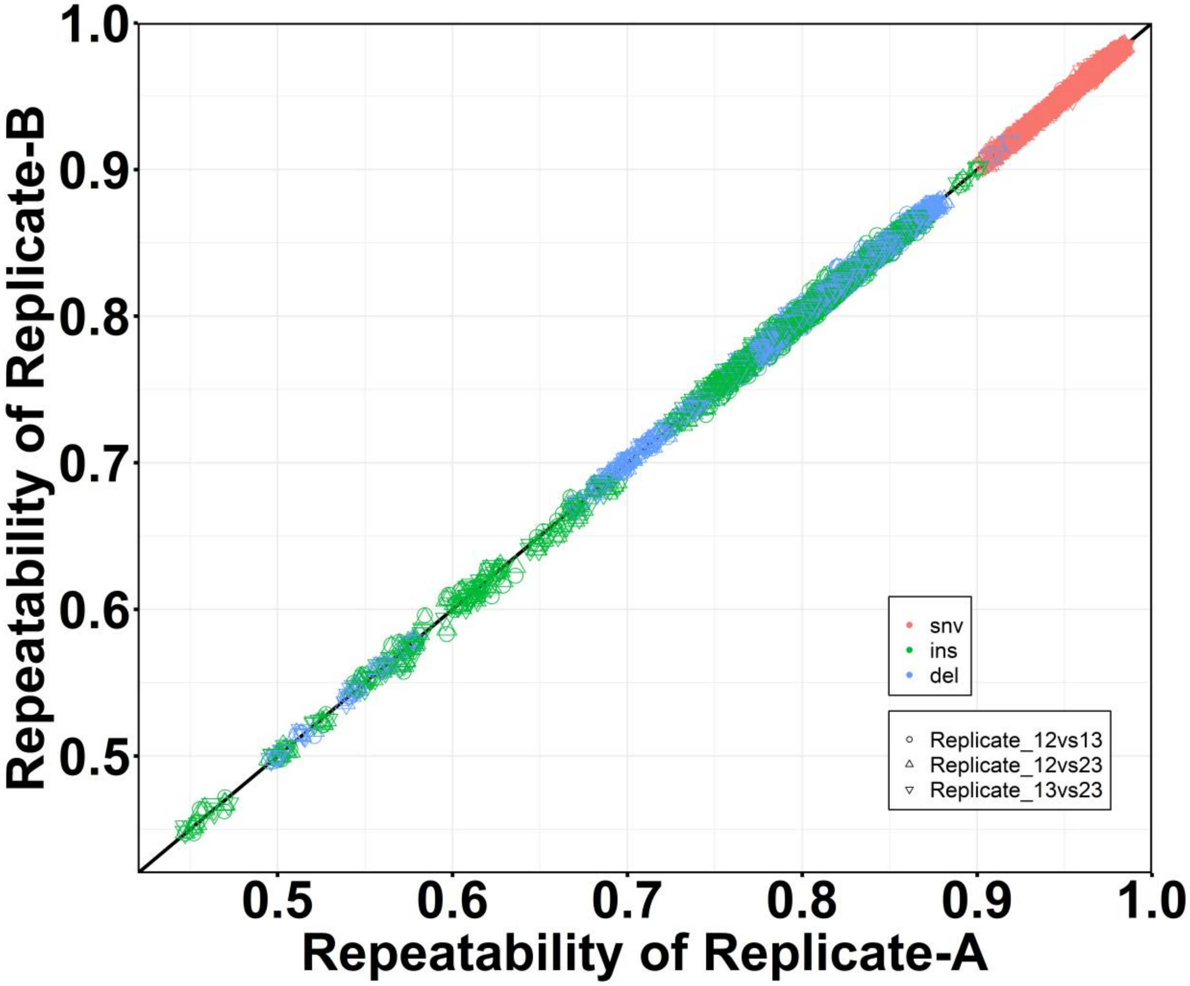

Fig. S9. Scatter plots of repeatability of replicate pairs of original Chinese Quartet sample data without filtering by HRRs. The points represent repeatability of two replicate pairs. Three types of variants are colored in three different colors: red for SNV, green for Insertion and blue for Deletion. The x-axis depicts repeatability calculated from one replicate pair, while the y-axis represents repeatability from the other replicate pair. Three comparison pairs presented in different shapes: circle for comparison between replicate pairs 1&2 and 1&3; upward triangles for replicate pairs 1&2 and 2&3; downward triangles for replicate pairs 1&3 and 2&3.

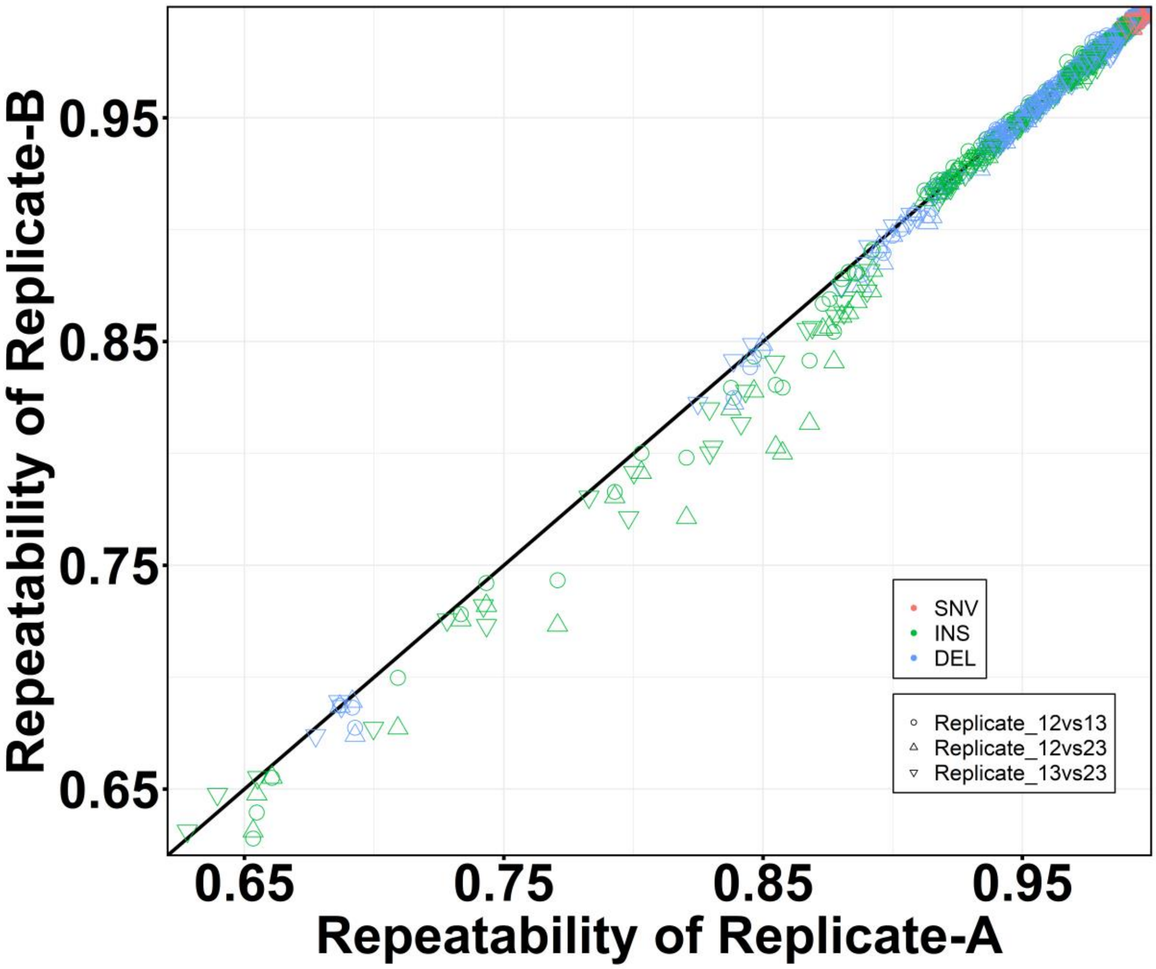

Fig. S10. Scatter plots of repeatability of replicate pairs of original data HapMap sample data with filtering by HRRs. The points represent repeatability of two replicate pairs. Three types of variants are colored in three different colors: red for SNV, green for Insertion and blue for Deletion. The x-axis depicts repeatability calculated from one replicate pair, while the y-axis represents repeatability from the other replicate pair. Three comparison pairs presented in different shapes: circle for comparison between replicate pairs 1&2 and 1&3; upward triangles for replicate pairs 1&2 and 2&3; downward triangles for replicate pairs 1&3 and 2&3.

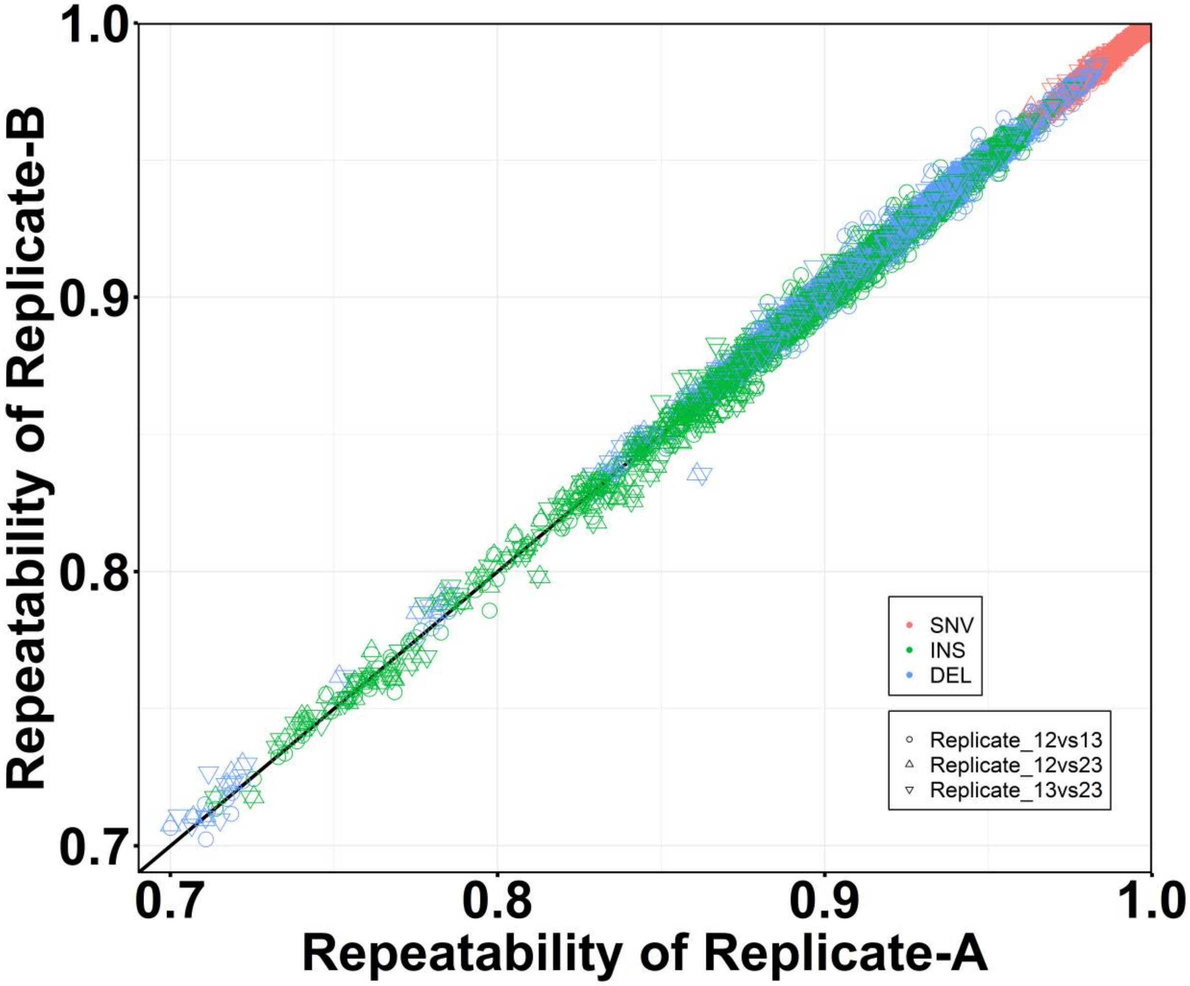

Fig. S11. Scatter plots of repeatability of replicate pairs of original Chinese Quartet sample data with filtering by HRRs. The points represent repeatability of two replicate pairs. Three types of variants are colored in three different colors: red for SNV, green for Insertion and blue for Deletion. The x-axis depicts repeatability calculated from one replicate pair, while the y-axis represents repeatability from the other replicate pair. Three comparison pairs presented in different shapes: circle for comparison between replicate pairs 1&2 and 1&3; upward triangles for replicate pairs 1&2 and 2&3; downward triangles for replicate pairs 1&3 and 2&3.

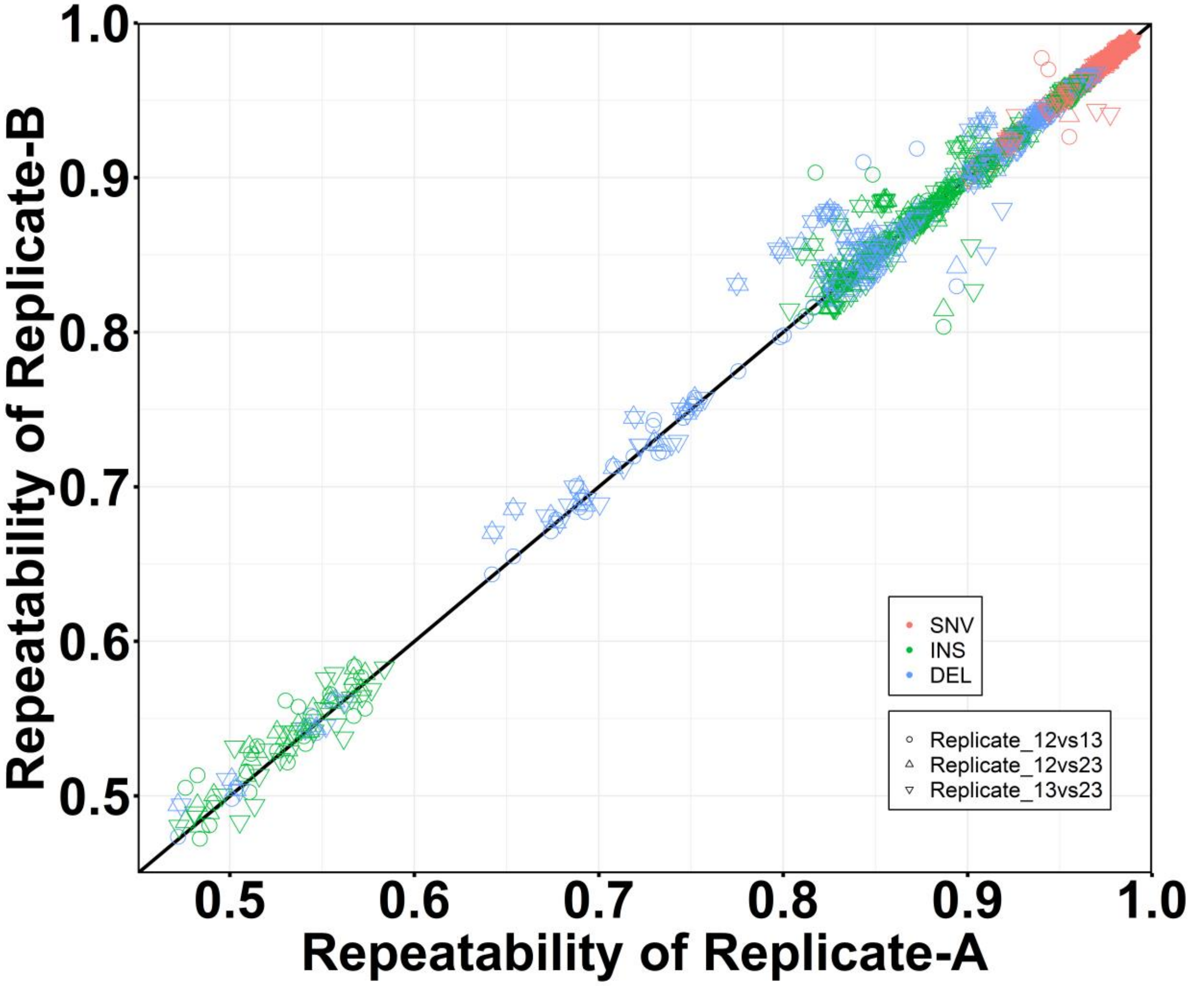

Fig. S12. Scatter plots of repeatability of replicate pairs of confirmatory HapMap sample data without filtering by HRRs. The points represent repeatability of two replicate pairs. Three types of variants are colored in three different colors: red for SNV, green for Insertion and blue for Deletion. The x-axis depicts repeatability calculated from one replicate pair, while the y-axis represents repeatability from the other replicate pair. Three comparison pairs presented in different shapes: circle for comparison between replicate pairs 1&2 and 1&3; upward triangles for replicate pairs 1&2 and 2&3; downward triangles for replicate pairs 1&3 and 2&3.

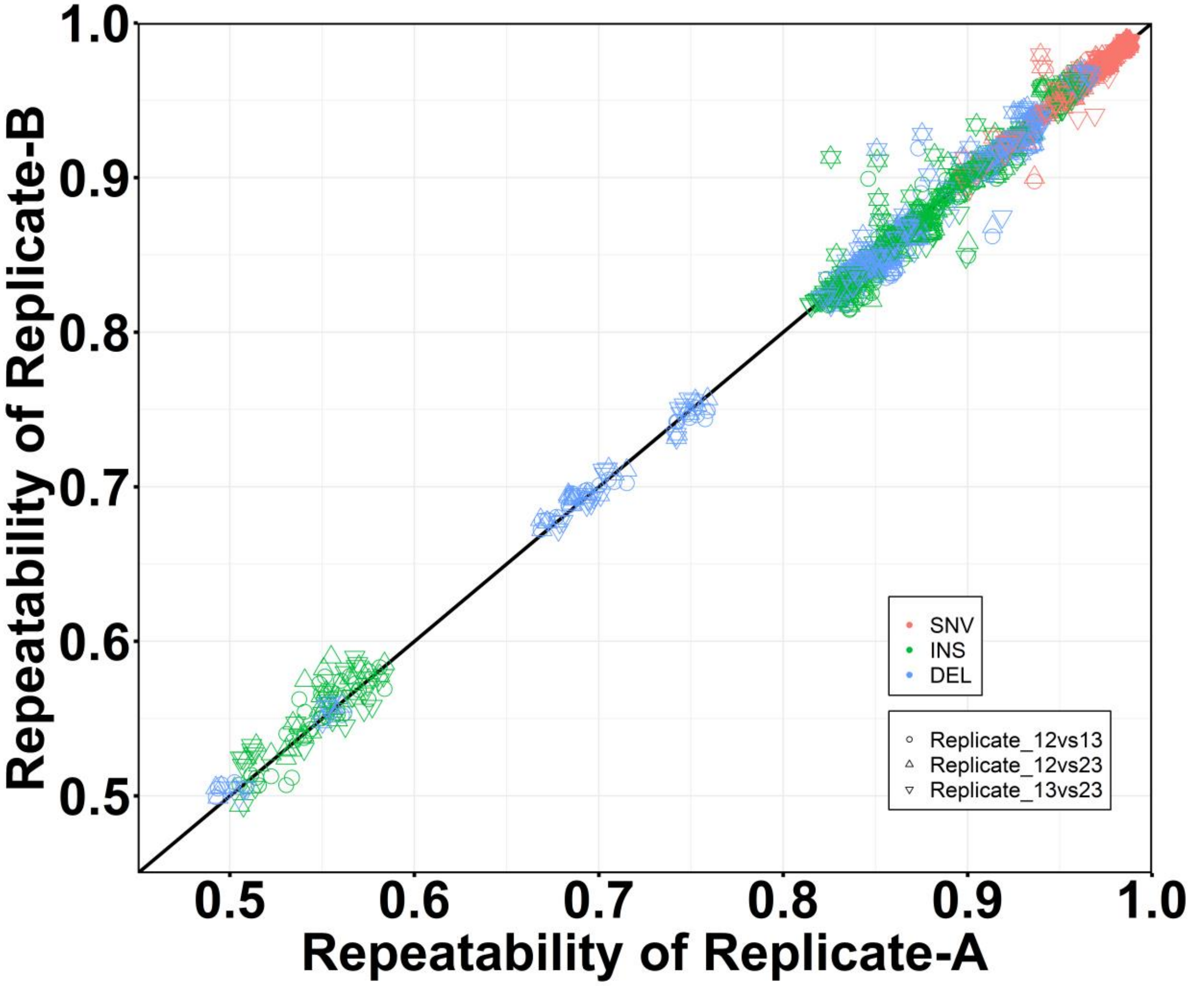

Fig. S13. Scatter plots of repeatability of replicate pairs of confirmatory Chinese Quartet sample data with filtering by HRRs. The points represent repeatability of two replicate pairs. Three types of variants are colored in three different colors: red for SNV, green for Insertion and blue for Deletion. The x-axis depicts repeatability calculated from one replicate pair, while the y-axis represents repeatability from the other replicate pair. Three comparison pairs presented in different shapes: circle for comparison between replicate pairs 1&2 and 1&3; upward triangles for replicate pairs 1&2 and 2&3; downward triangles for replicate pairs 1&3 and 2&3.

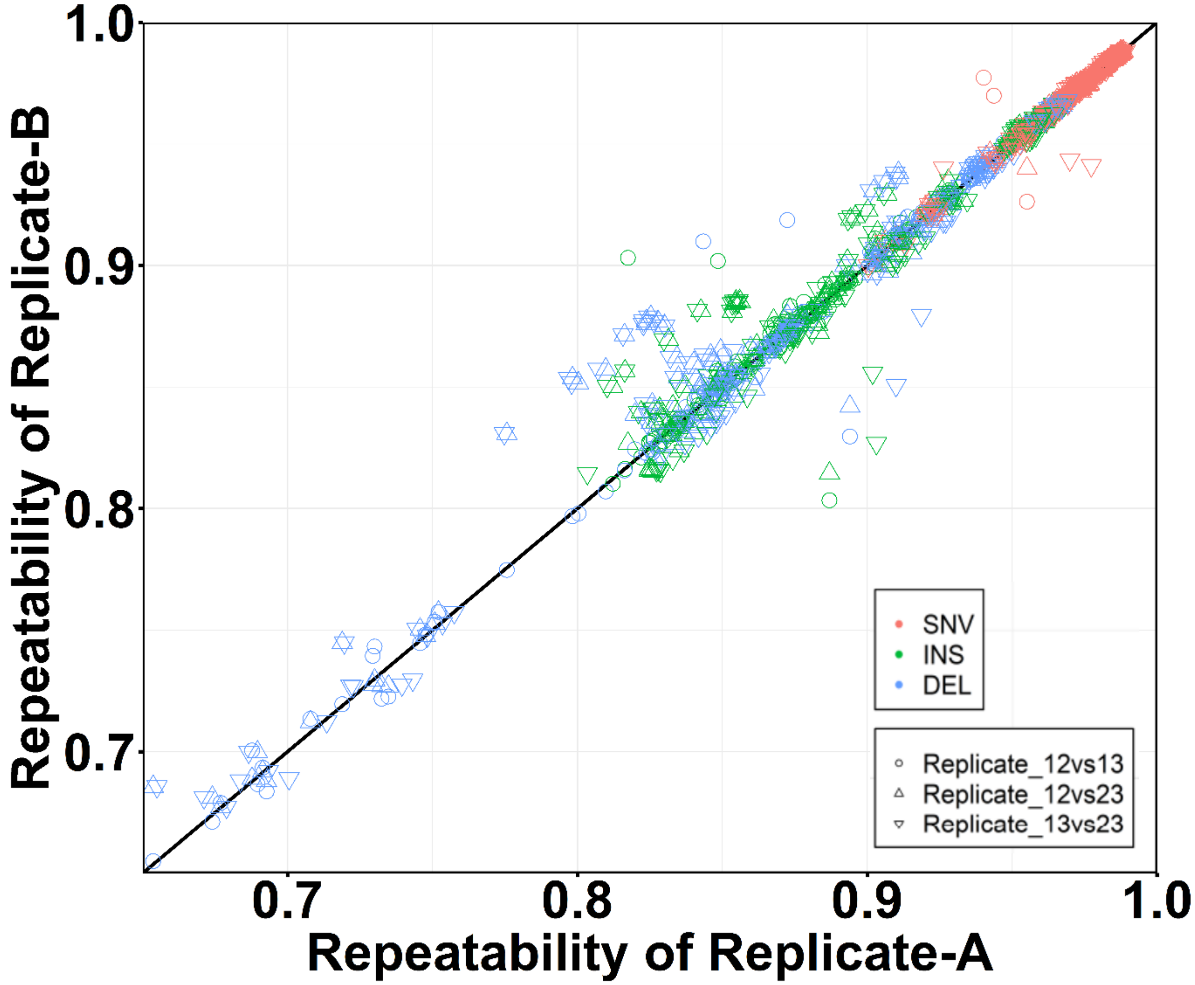

Fig. S14. Scatter plots of repeatability of replicate pairs of confirmatory HapMap sample data with filtering by HRRs. The points represent repeatability of two replicate pairs. Three types of variants are colored in three different colors: red for SNV, green for Insertion and blue for Deletion. The x-axis depicts repeatability calculated from one replicate pair, while the y-axis represents repeatability from the other replicate pair. Three comparison pairs presented in different shapes: circle for comparison between replicate pairs 1&2 and 1&3; upward triangles for replicate pairs 1&2 and 2&3; downward triangles for replicate pairs 1&3 and 2&3.

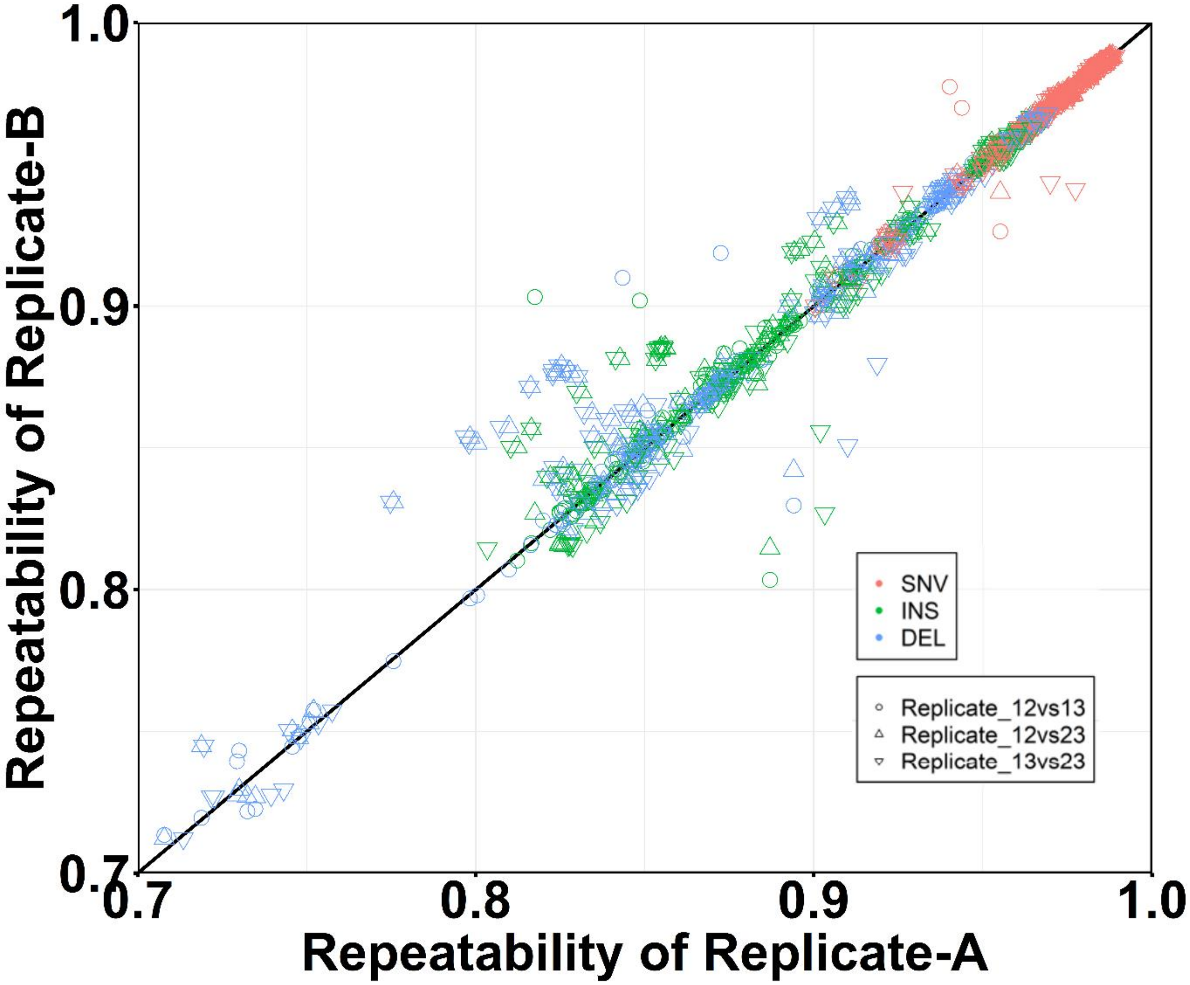

Fig. S15. Scatter plots of repeatability of replicate pairs of confirmatory Chinese Quartet sample data with filtering by HRRs. The points represent repeatability of two replicate pairs. Three types of variants are colored in three different colors: red for SNV, green for Insertion and blue for Deletion. The x-axis depicts repeatability calculated from one replicate pair, while the y-axis represents repeatability from the other replicate pair. Three comparison pairs presented in different shapes: circle for comparison between replicate pairs 1&2 and 1&3; upward triangles for replicate pairs 1&2 and 2&3; downward triangles for replicate pairs 1&3 and 2&3.

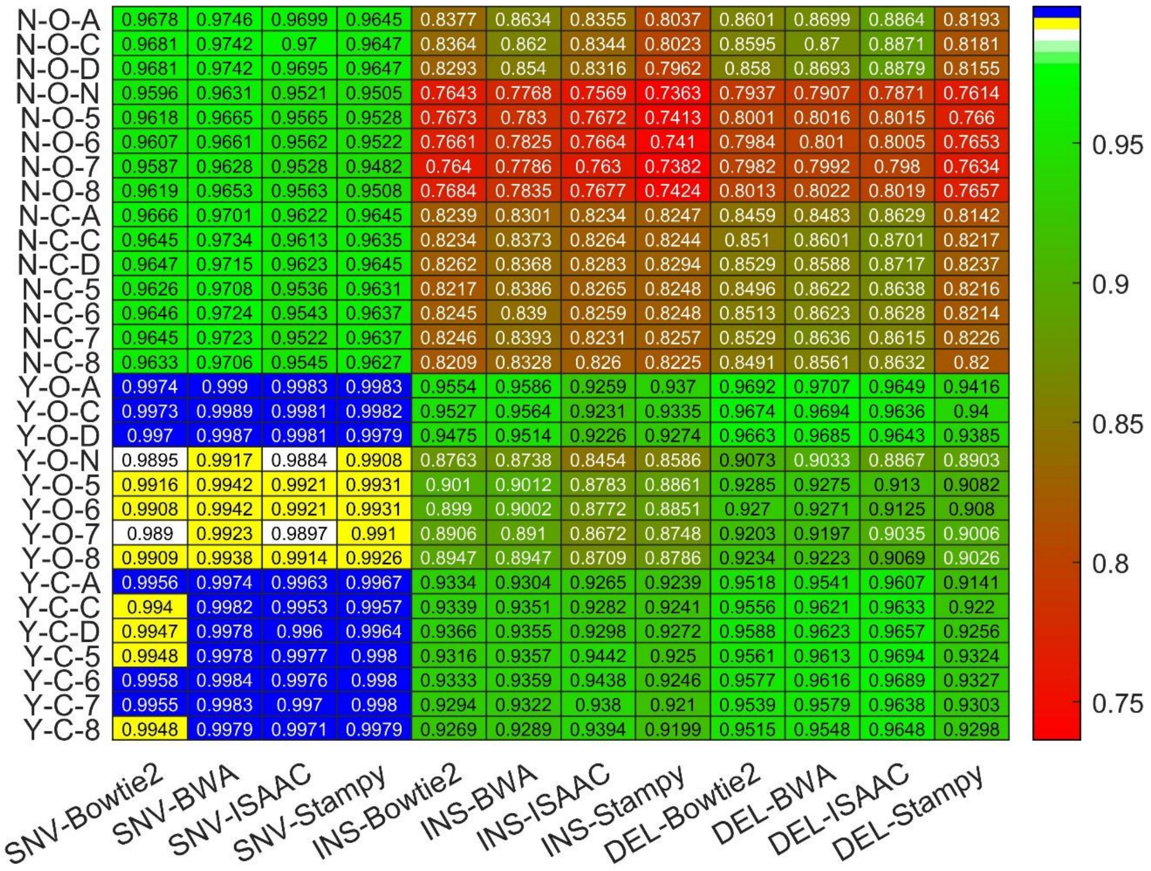

Fig. S16. Technical repeatability for aligners. The average technical repeatability values are calculated for different types of call sets (given in the row names) from the same aligners (depicted in second part of the column names) and displayed in the heatmap with color legend shown in the right. The first 3 columns are repeatability in SNVs. The middle three columns are repeatability in insertions. The last 3 columns are repeatability in deletions. The row name are coded in three letters: the first letter indicates the call sets are filtered with HRRs (Y) or not (N); the second letter denotes the call sets are from original study (O) or confirmatory study (C); the third letter gives samples (A: NA10835; C: NA12248; D: NA12249; N: NA12878; 5 and 6: Chinese quartet twin daughters; 7: Chinese quartet father; 8: Chinese mother).

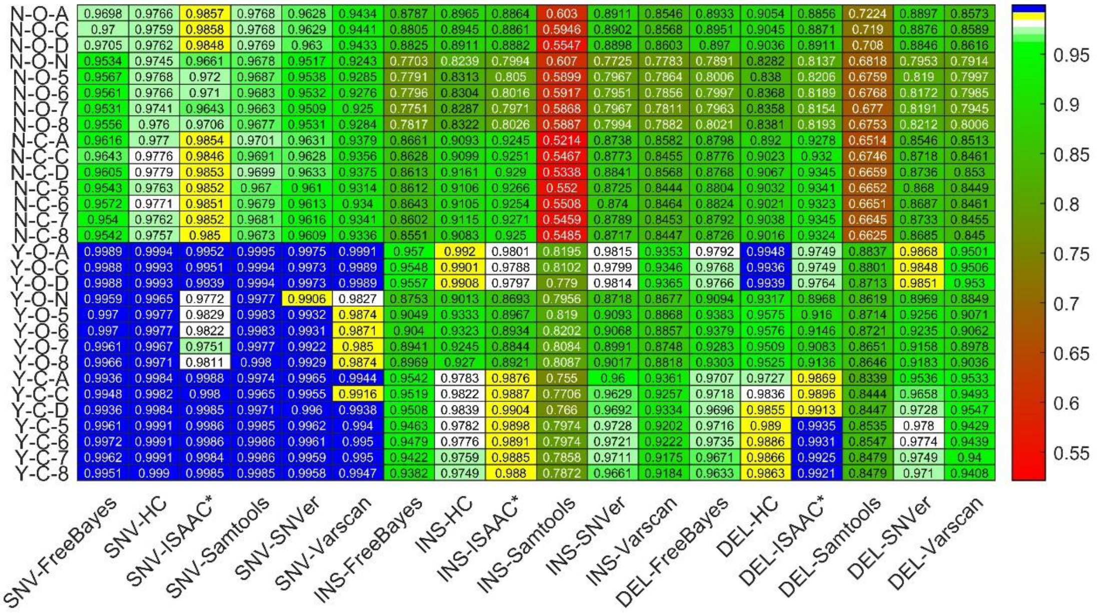

	SNV-FreeBayes	SNV-HC	SNV-ISAAC*	SNV-Samtools	SNV-SNVer	SNV-Varscan	INS-FreeBayes	INS-HC	INS-ISAAC*	INS-Samtools	INS-SNVer	INS-Varscan	DEL-FreeBayes	DEL-HC	DEL-ISAAC*	DEL-Samtools	DEL-SNVer	DEL-Varscan
N-O-A	0.9698	0.9766	0.9857	0.9768	0.9628	0.9434	0.8787	0.8965	0.8864	0.603	0.8911	0.8546	0.8933	0.9054	0.8856	0.7224	0.8897	0.8573
N-O-C	0.97	0.9759	0.9858	0.9768	0.9629	0.9441	0.8805	0.8945	0.8861	0.5946	0.8902	0.8568	0.8951	0.9045	0.8871	0.719	0.8876	0.8589
N-O-D	0.9705	0.9762	0.9848	0.9769	0.963	0.9433	0.8825	0.8911	0.8882	0.5547	0.8898	0.8603	0.897	0.9036	0.8911	0.708	0.8846	0.8616
N-O-N	0.9534	0.9745	0.9661	0.9678	0.9517	0.9243	0.7703	0.8239	0.7994	0.607	0.7725	0.7783	0.7891	0.8282	0.8137	0.6818	0.7953	0.7914
N-O-5	0.9567	0.9768	0.972	0.9687	0.9538	0.9285	0.7791	0.8313	0.805	0.5899	0.7967	0.7864	0.8006	0.838	0.8206	0.6759	0.819	0.7997
N-O-6	0.9561	0.9766	0.971	0.9683	0.9532	0.9276	0.7796	0.8304	0.8016	0.5917	0.7951	0.7856	0.7997	0.8368	0.8189	0.6768	0.8172	0.7985
N-O-7	0.9531	0.9741	0.9643	0.9663	0.9509	0.925	0.7751	0.8287	0.7971	0.5868	0.7967	0.7811	0.7963	0.8358	0.8154	0.677	0.8191	0.7945
N-O-8	0.9556	0.976	0.9706	0.9677	0.9531	0.9284	0.7817	0.8322	0.8026	0.5887	0.7994	0.7882	0.8021	0.8381	0.8193	0.6753	0.8212	0.8006
N-C-A	0.9616	0.977	0.9854	0.9701	0.9631	0.9379	0.8661	0.9093	0.9245	0.5214	0.8738	0.8582	0.8798	0.892	0.9278	0.6514	0.8546	0.8513
N-C-C	0.9643	0.9776	0.9846	0.9691	0.9628	0.9356	0.8628	0.9099	0.9251	0.5467	0.8773	0.8455	0.8776	0.9023	0.932	0.6746	0.8718	0.8461
N-C-D	0.9605	0.9779	0.9853	0.9699	0.9633	0.9375	0.8613	0.9161	0.929	0.5338	0.8841	0.8568	0.8768	0.9067	0.9345	0.6659	0.8736	0.853
N-C-5	0.9543	0.9763	0.9852	0.967	0.961	0.9314	0.8612	0.9106	0.9266	0.552	0.8725	0.8444	0.8804	0.9032	0.9341	0.6652	0.868	0.8449
N-C-6	0.9572	0.9771	0.9851	0.9679	0.9613	0.934	0.8643	0.9105	0.9254	0.5508	0.874	0.8464	0.8824	0.9021	0.9323	0.6651	0.8687	0.8461
N-C-7	0.954	0.9762	0.9852	0.9681	0.9616	0.9341	0.8602	0.9115	0.9271	0.5459	0.8789	0.8453	0.8792	0.9038	0.9345	0.6645	0.8733	0.8455
N-C-8	0.9542	0.9757	0.985	0.9673	0.9609	0.9336	0.8551	0.9083	0.925	0.5485	0.8717	0.8447	0.8726	0.9016	0.9324	0.6625	0.8685	0.845
Y-O-A	0.9989	0.9994	0.9952	0.9995	0.9975	0.9991	0.957	0.992	0.9801	0.8195	0.9815	0.9353	0.9792	0.9948	0.9749	0.8837	0.9868	0.9501
Y-O-C	0.9988	0.9993	0.9951	0.9994	0.9973	0.9989	0.9548	0.9901	0.9788	0.8102	0.9799	0.9346	0.9768	0.9936	0.9749	0.8801	0.9848	0.9506
Y-O-D	0.9988	0.9993	0.9939	0.9994	0.9973	0.9989	0.9557	0.9908	0.9797	0.779	0.9814	0.9365	0.9766	0.9939	0.9764	0.8713	0.9851	0.953
Y-O-N	0.9959	0.9965	0.9772	0.9977	0.9906	0.9827	0.8753	0.9013	0.8693	0.7956	0.8718	0.8677	0.9094	0.9317	0.8968	0.8619	0.8969	0.8849
Y-O-5	0.997	0.9977	0.9829	0.9983	0.9932	0.9874	0.9049	0.9333	0.8967	0.819	0.9093	0.8868	0.9383	0.9575	0.916	0.8714	0.9256	0.9071
Y-O-6	0.997	0.9977	0.9822	0.9983	0.9931	0.9871	0.904	0.9323	0.8934	0.8202	0.9068	0.8857	0.9379	0.9576	0.9146	0.8721	0.9235	0.9062
Y-O-7	0.9961	0.9967	0.9751	0.9977	0.9922	0.985	0.8941	0.9245	0.8844	0.8084	0.8991	0.8748	0.9283	0.9509	0.9083	0.8651	0.9158	0.8978
Y-O-8	0.9966	0.9971	0.9811	0.998	0.9929	0.9874	0.8969	0.927	0.8921	0.8087	0.9017	0.8818	0.9303	0.9525	0.9136	0.8646	0.9183	0.9036
Y-C-A	0.9936	0.9984	0.9988	0.9974	0.9965	0.9944	0.9542	0.9783	0.9876	0.755	0.96	0.9361	0.9707	0.9727	0.9869	0.8339	0.9536	0.9533
Y-C-C	0.9948	0.9982	0.998	0.9965	0.9955	0.9916	0.9519	0.9822	0.9887	0.7706	0.9629	0.9257	0.9718	0.9836	0.9896	0.8444	0.9658	0.9493
Y-C-D	0.9936	0.9984	0.9985	0.9971	0.996	0.9938	0.9508	0.9839	0.9904	0.766	0.9692	0.9334	0.9696	0.9855	0.9913	0.8447	0.9728	0.9547
Y-C-5	0.9961	0.9991	0.9986	0.9985	0.9962	0.994	0.9463	0.9782	0.9898	0.7974	0.9728	0.9202	0.9716	0.989	0.9935	0.8535	0.978	0.9429
Y-C-6	0.9972	0.9991	0.9986	0.9986	0.9961	0.995	0.9479	0.9776	0.9891	0.7974	0.9721	0.9222	0.9735	0.9886	0.9931	0.8547	0.9774	0.9439
Y-C-7	0.9962	0.9991	0.9984	0.9986	0.9959	0.995	0.9422	0.9759	0.9885	0.7858	0.9711	0.9175	0.9671	0.9866	0.9925	0.8479	0.9749	0.94
Y-C-8	0.9951	0.999	0.9985	0.9985	0.9958	0.9947	0.9382	0.9749	0.988	0.7872	0.9661	0.9184	0.9633	0.9863	0.9921	0.8479	0.971	0.9408

Fig. S17. Technical repeatability for callers. The average technical repeatability values are calculated for different types of call sets (given in the row names) from the same callers (depicted in second part of the column names; ISAAC* indicates the call sets in original study are from ISAAC but the call sets from the confirmatory study are from Strelka) and displayed in the heatmap with color legend shown in the right. The first 3 columns are repeatability in SNVs. The middle 3 columns are repeatability in insertions. The last 3 columns are repeatability in deletions. The row name are coded in 3 letters: the first letter indicates the call sets are filtered with HRRs (Y) or not (N); the second letter denotes the call sets are from original study (O) or confirmatory study (C); the third letter gives samples (A: NA10835; C: NA12248; D: NA12249; N: NA12878; 5 and 6: Chinese quartet twin daughters; 7: Chinese quartet father; 8: Chinese mother).

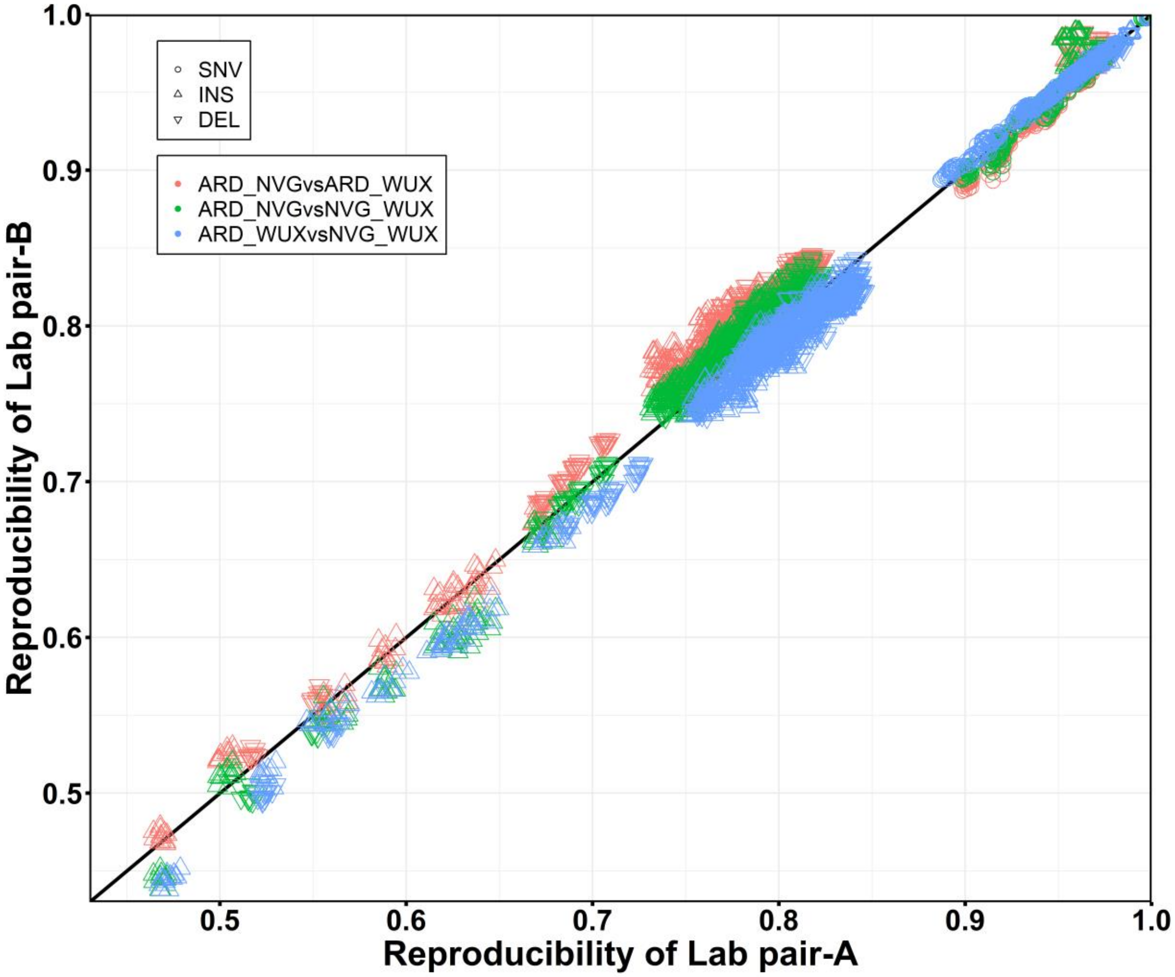

Fig. S18. Scatter plot of lab pair reproducibility for original CQ set without filtering by HRRs. The points represent lab reproducibility of two lab pairs for the same replicate. The x-axis depicts reproducibility calculated from one optional lab pair, while y-axis represent reproducibility from the other optional lab pair. Three type of variants are plotted in three different shapes: circle for SNV, triangle point up for (INS) Insertion and triangle point down for DEL(Deletion). Three comparison pair presented in different colors: red for comparison between reproducibility from lab pair ARD_NVG and reproducibility from lab pair ARD_WUX; green for comparison between reproducibility from lab pair ARD_NVG and reproducibility from lab pair NVG_WUX; blue for comparison reproducibility from lab pair ARD_WUX and reproducibility from lab pair NVG_WUX.

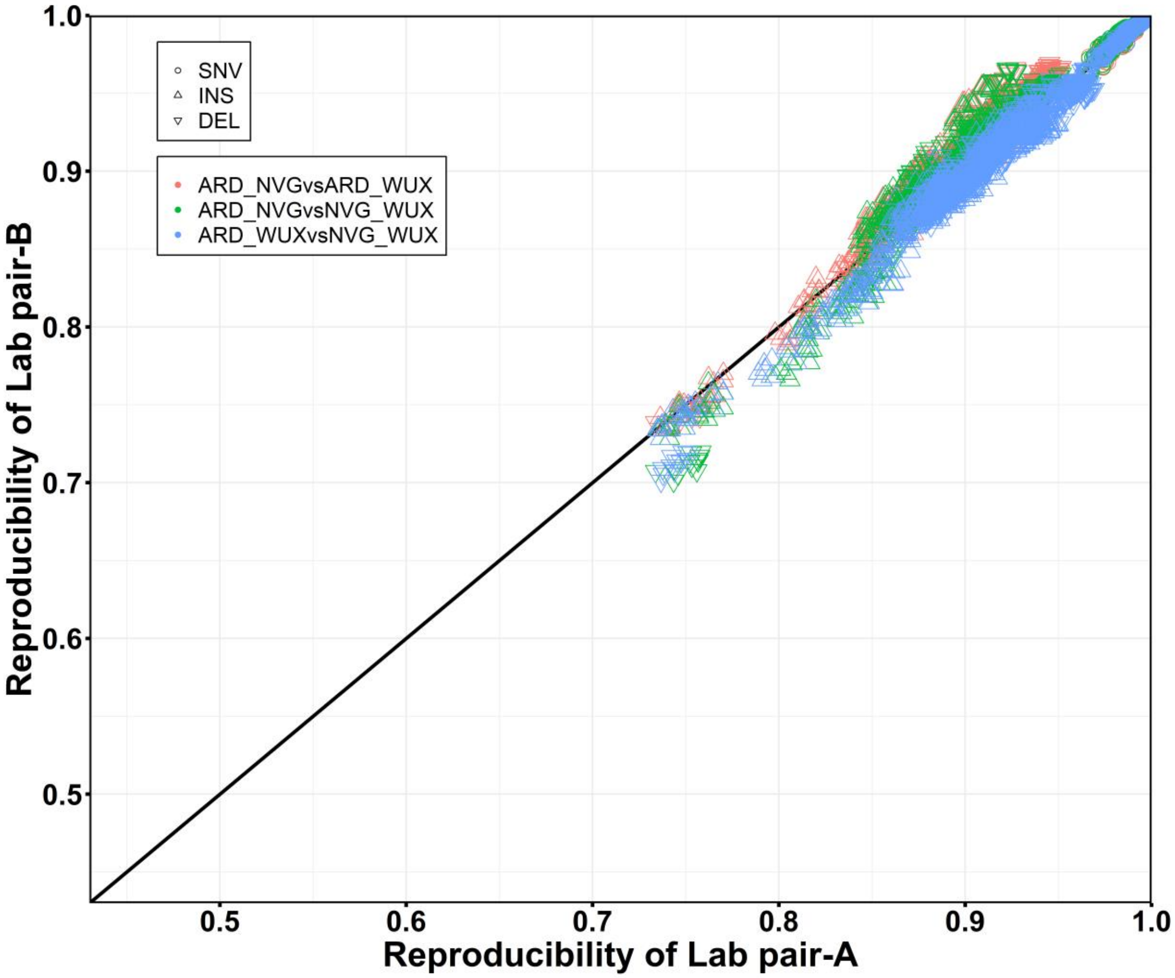

Fig. S19. Scatter plot of lab pair reproducibility for original CQ set with filtering by HRRs. The points represent lab reproducibility of two lab pairs for the same replicate. The x-axis depicts reproducibility calculated from one optional lab pair, while y-axis represent reproducibility from the other optional lab pair. Three type of variants are plotted in three different shapes: circle for SNV, triangle point up for (INS) Insertion and triangle point down for DEL(Deletion). Three comparison pair presented in different colors: red for comparison between reproducibility from lab pair ARD_NVG and reproducibility from lab pair ARD_WUX; green for comparison between reproducibility from lab pair ARD_NVG and reproducibility from lab pair NVG_WUX; blue for comparison reproducibility from lab pair ARD_WUX and reproducibility from lab pair NVG_WUX.

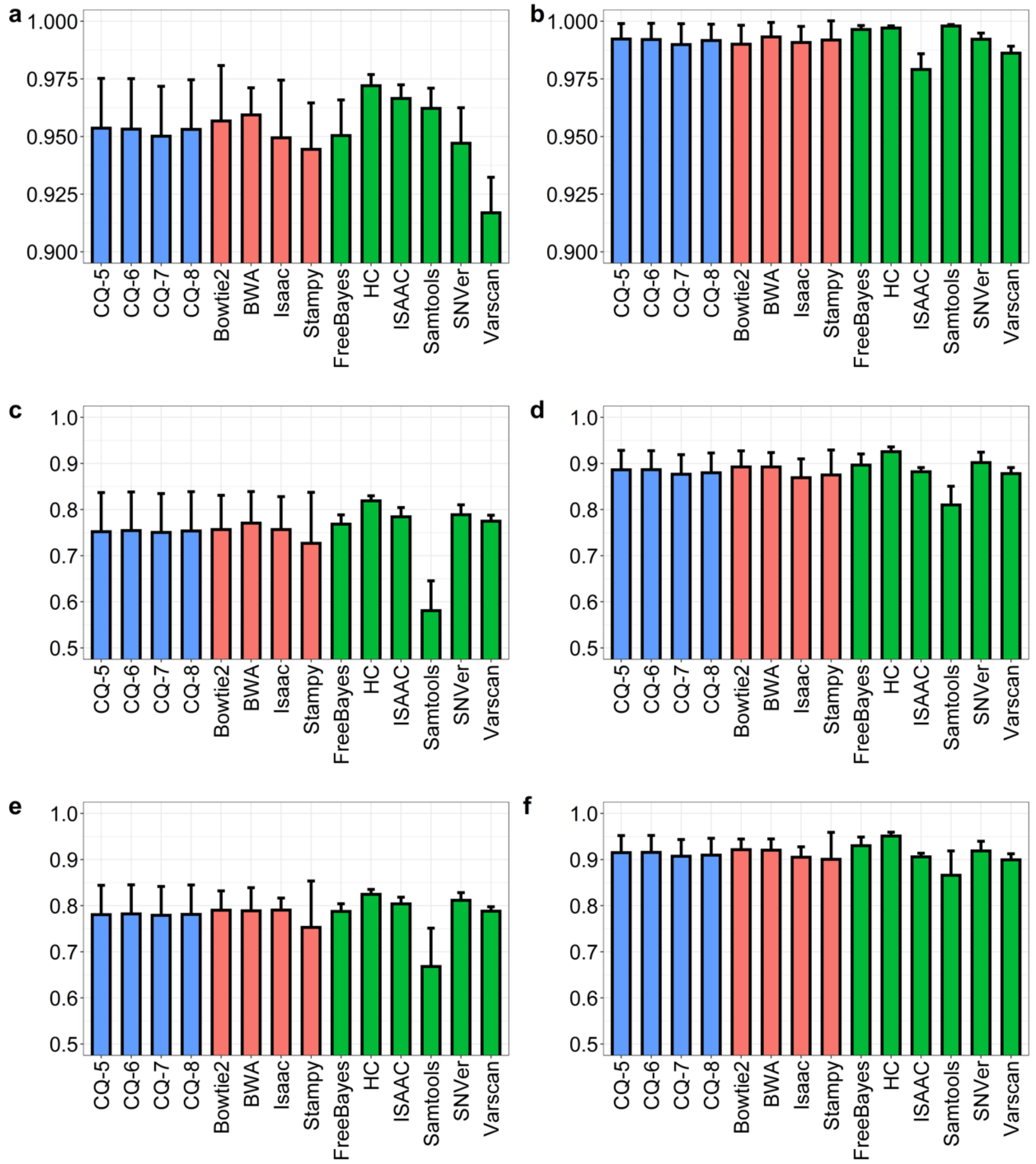

Fig. S20. Lower bound of lab reproducibility (y-axes) of SNVs (**a**), upper bound of lab reproducibility (y-axes) of SNVs (**b**), lower bound of lab reproducibility (y-axes) of insertions (**c**), upper bound of lab reproducibility (y-axes) of insertions (**d**), lower bound of lab reproducibility (y-axes) of deletions (**e**), and upper bound of lab reproducibility (y-axes) of deletions (**f**). The bars represent means and the sticks give standard deviations of lab reproducibility values for the samples (blue), aligners (red) and callers (green) indicated at the x-axes.

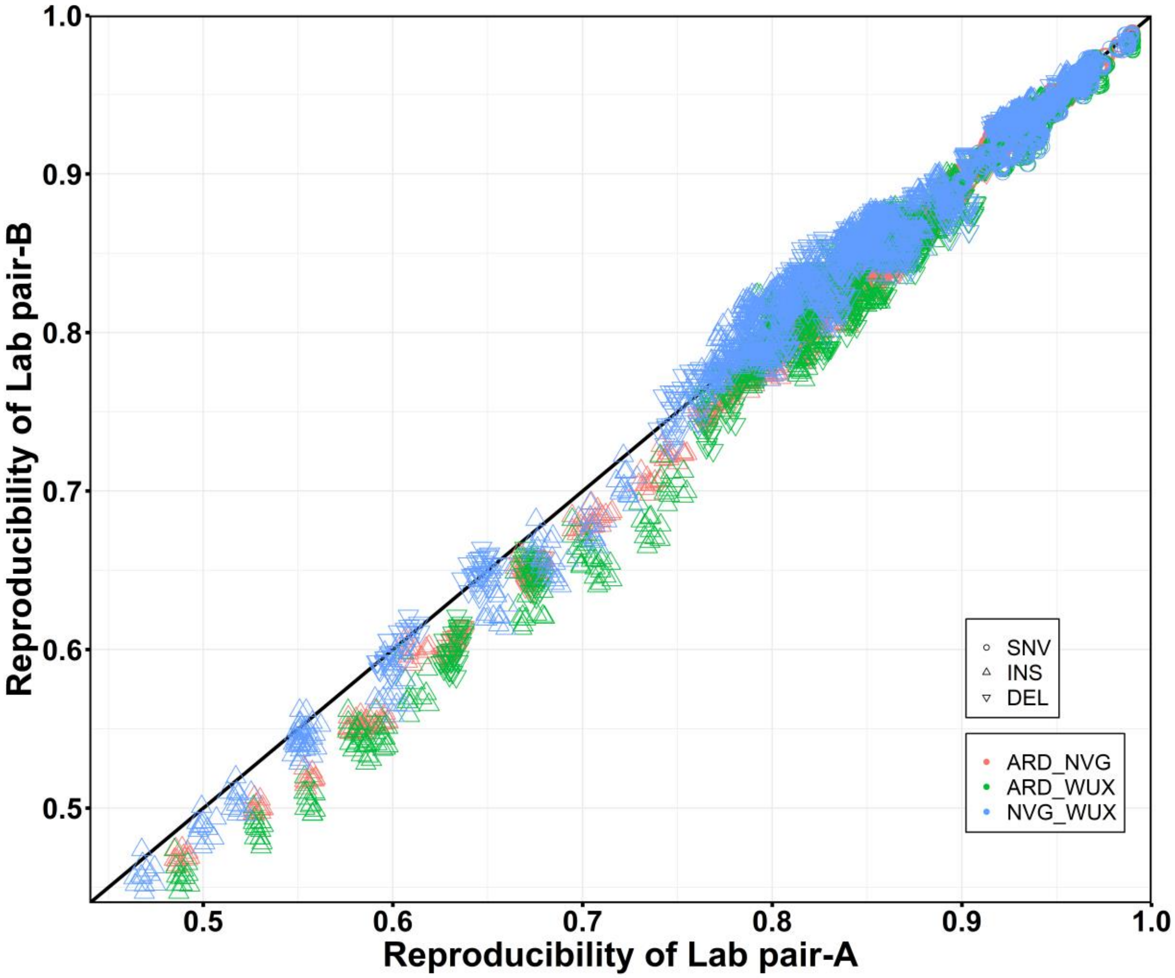

Fig. S21. Scatter plot for lab pair's impact on aligner reproducibility for original CQ call sets without filtering by HRRs. The points represent aligner reproducibility in three lab pairs for various sample set. Three type of variants are plotted in three different shapes: circle for SNV, up triangle for INS (Insertion) and down triangle for DEL(Deletion). The x-axis depicts aligner reproducibility calculated from one lab, while y-axis represent aligner reproducibility from the other lab. Three comparison pair presented in different colors: red for ARD versus NVG, green for ARD versus NVG, blue for NVG versus WUX.

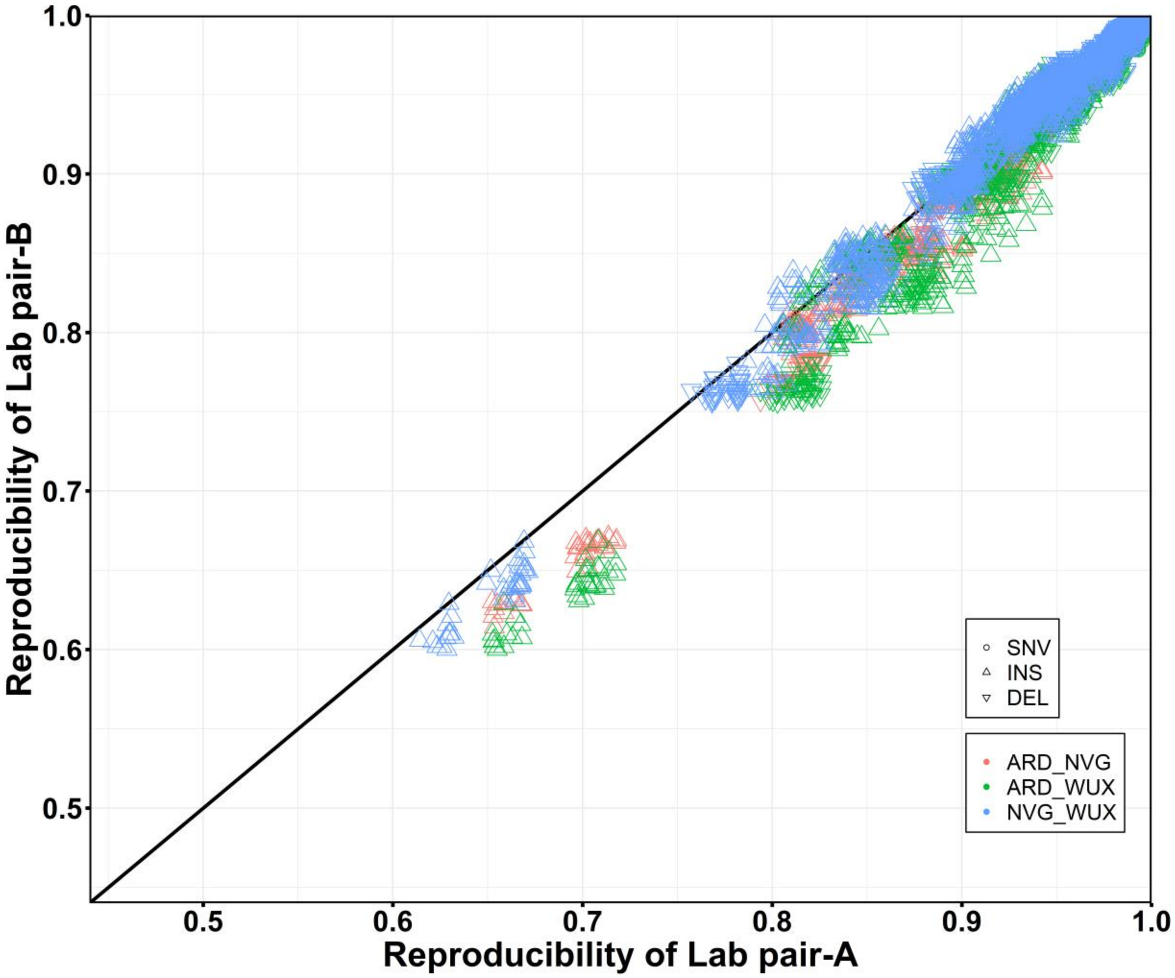

Fig. S22. Scatter plot for lab pair's impact on aligner reproducibility for original CQ call sets with filtering by HRRs. The points represent aligner reproducibility in three lab pairs for various sample set. Three type of variants are plotted in three different shapes: circle for SNV, up triangle for INS (Insertion) and down triangle for DEL(Deletion). The x-axis depicts aligner reproducibility calculated from one lab, while y-axis represent aligner reproducibility from the other lab. Three comparison pair presented in different colors: red for ARD versus NVG, green for ARD versus NVG, blue for NVG versus WUX.

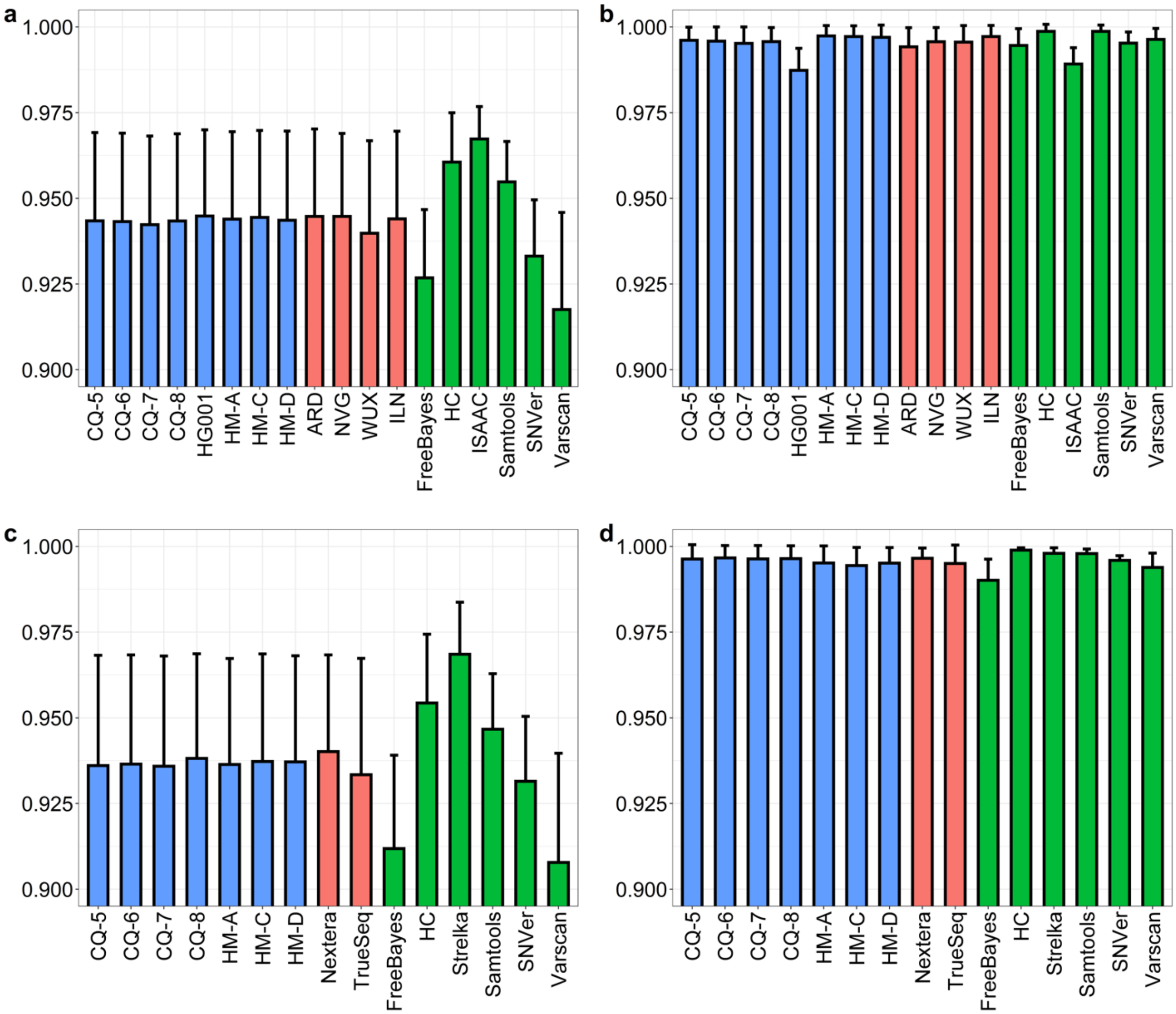

Fig. S23. Aligner reproducibility in SNVs (y-axes). The bars represent means and the sticks give standard deviations of aligner reproducibility values for the samples (blue, HM-A: NA10835; HM-C: NA12248; HM-D: NA12249; HG001: GIAB reference sample NA12878; CQ-5 and CQ-6: Chinese quartet twin daughters; CQ-7: Chinese quartet father; CQ8: Chinese mother), labs/library kits (red) and callers (green) indicated at the x-axes. **a**) lower bound of reproducibility from original data; **b**) upper bound reproducibility of original data; **c**) lower bound of reproducibility from confirmatory data; **d**) upper bound of reproducibility from confirmatory data.

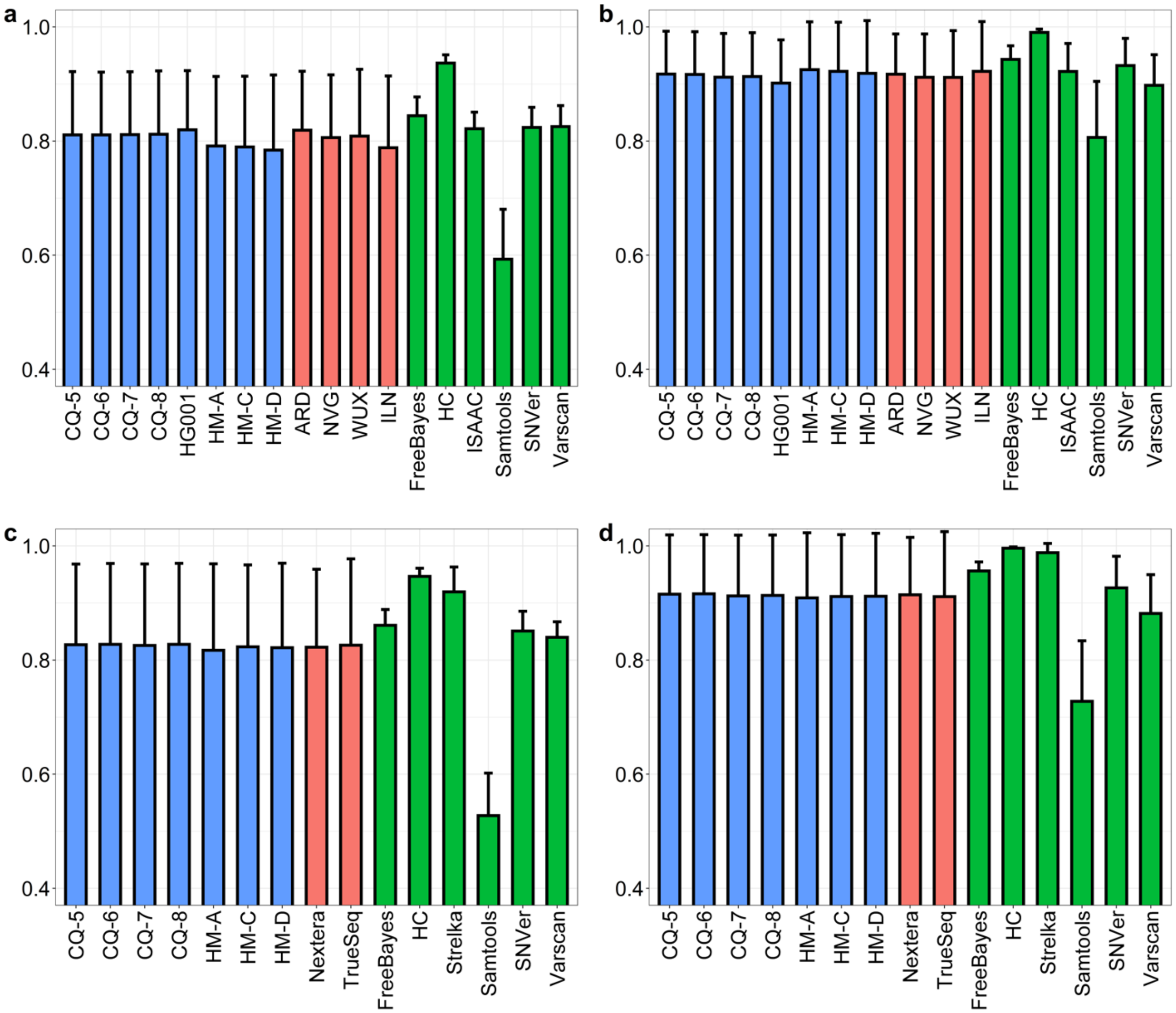

Fig. S24. Aligner reproducibility in insertions (y-axes). The bars represent means and the sticks give standard deviations of aligner reproducibility values for the samples (blue, HM-A: NA10835; HM-C: NA12248; HM-D: NA12249; HG001: GIAB reference sample NA12878; CQ-5 and CQ-6: Chinese quartet twin daughters; CQ-7: Chinese quartet father; CQ8: Chinese quartet mother), labs/library kits (red) and callers (green) indicated at the x-axes. **a**) lower bound of aligner reproducibility from original data; **b**) upper bound of aligner reproducibility from original data; **c**) lower bound of aligner reproducibility from confirmatory data; **d**) upper bound of aligner reproducibility from confirmatory data.

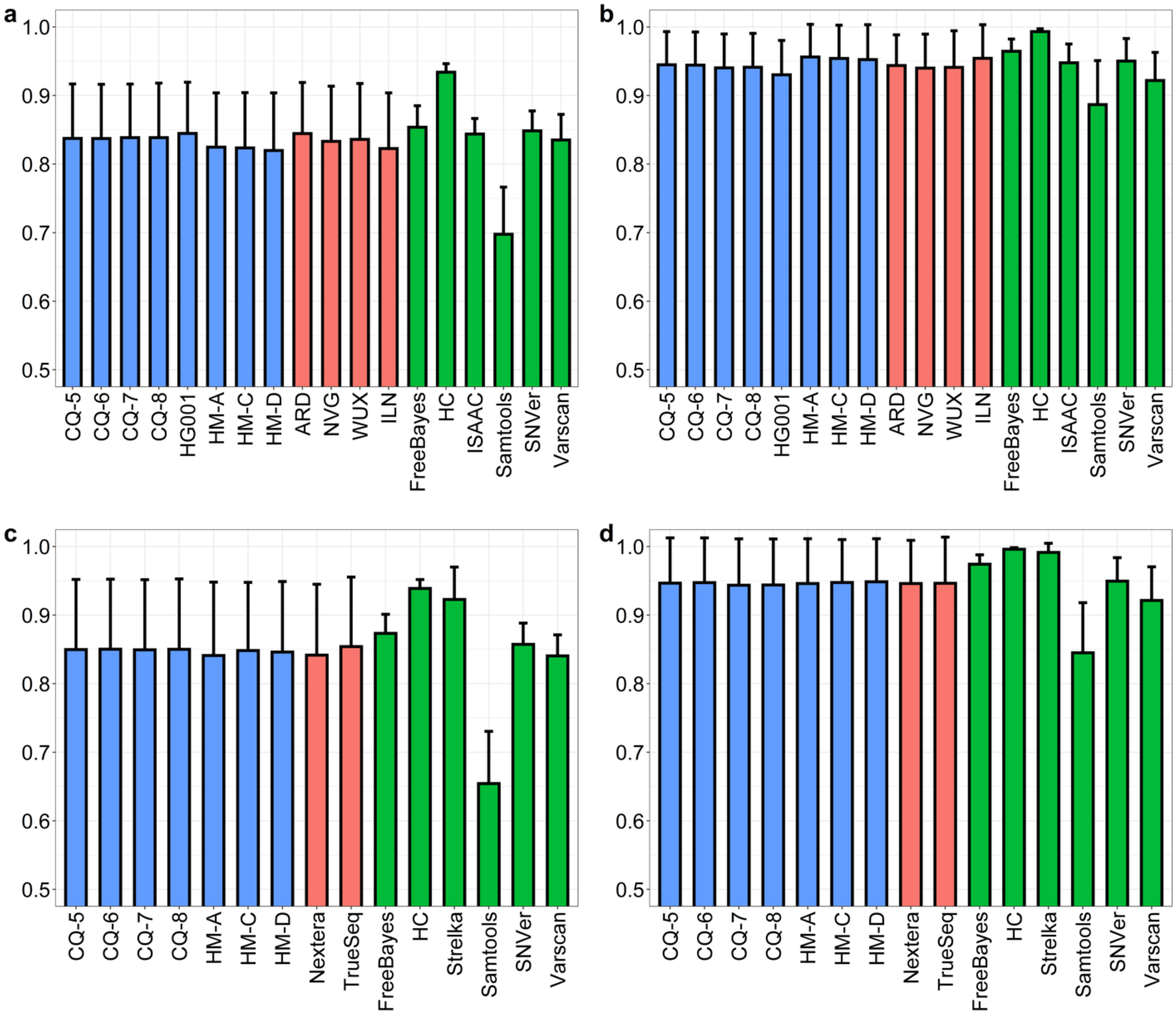

Fig. S25. Aligner reproducibility in deletions (y-axes). The bars represent means and the sticks give standard deviations of aligner reproducibility values for the samples (blue, HM-A: NA10835; HM-C: NA12248; HM-D: NA12249; HG001: GIAB reference sample NA12878; CQ-5 and CQ-6: Chinese quartet twin daughters; CQ-7: Chinese quartet father; CQ8: Chinese mother), labs/library kits (red) and callers (green) indicated at the x-axes. **a**) lower bound of aligner reproducibility from original data; **b**) upper bound of aligner reproducibility from original data; **c**) lower bound of aligner reproducibility from confirmatory data; **d**) upper bound of aligner reproducibility from confirmatory data.

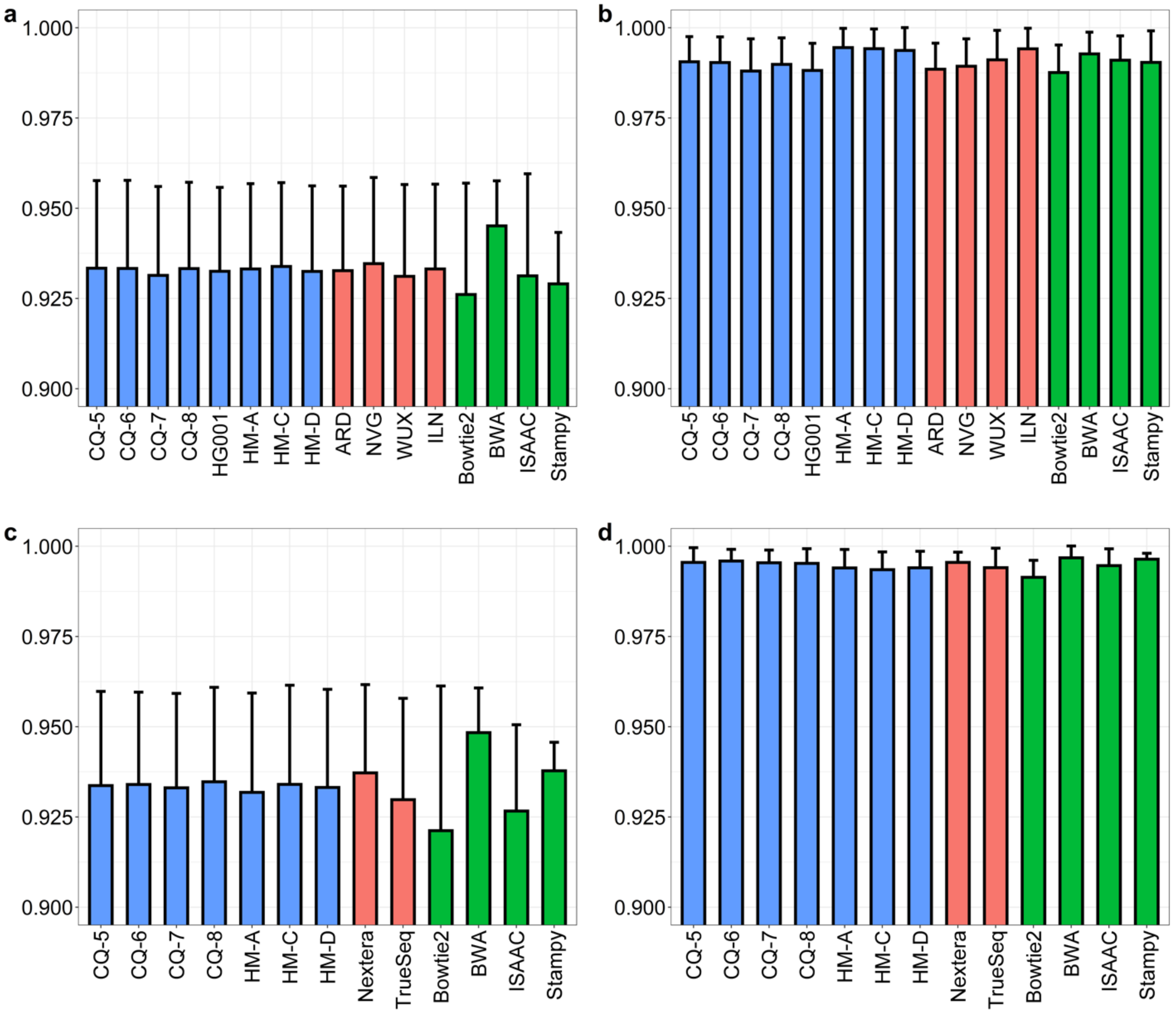

Fig. S26. Caller reproducibility in SNVs (y-axes). The bars represent means and the sticks give standard deviations of caller reproducibility values for the samples (blue, HM-A: NA10835; HM-C: NA12248; HM-D: NA12249; HG001: GIAB reference sample NA12878; CQ-5 and CQ-6: Chinese quartet twin daughters; CQ-7: Chinese quartet father; CQ8: Chinese quartet mother), labs/library kits (red) and aligners (green) indicated at the x-axes. **a**) lower bound of caller reproducibility from original data; **b**) upper bound of caller reproducibility from original data; **c**) lower bound of caller reproducibility from confirmatory data; **d**) upper bound of caller reproducibility from confirmatory data.

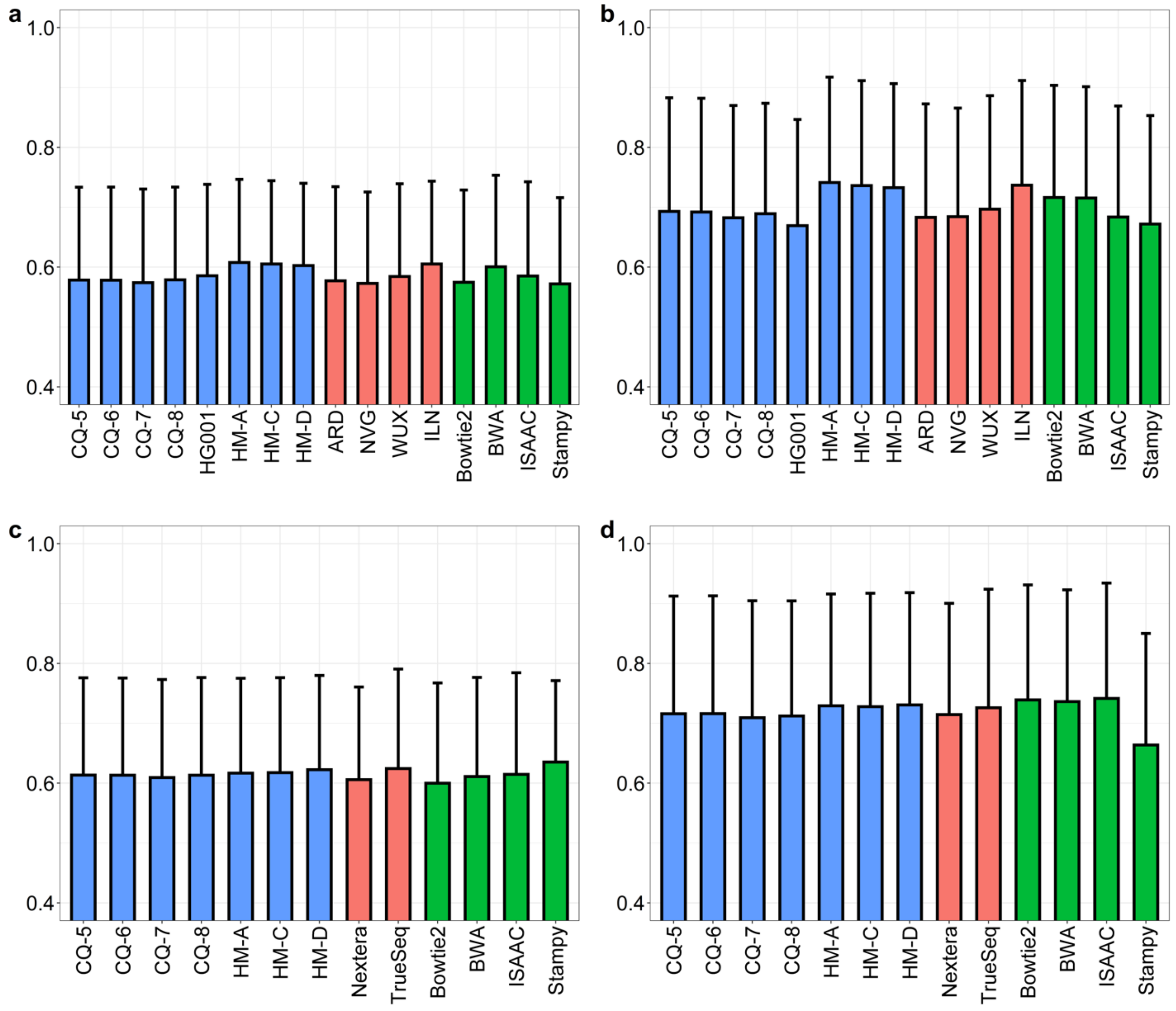

Fig. S27. Caller reproducibility in insertions (y-axes). The bars represent means and the sticks give standard deviations of caller reproducibility values for the samples (blue, HM-A: NA10835; HM-C: NA12248; HM-D: NA12249; HG001: GIAB reference sample NA12878; CQ-5 and CQ-6: Chinese quartet twin daughters; CQ-7: Chinese quartet father; CQ8: Chinese quartet mother), labs/library kits (red) and aligners (green) indicated at the x-axes. **a**) lower bound of caller reproducibility from original data; **b**) upper bound of caller reproducibility from original data; **c**) lower bound of caller reproducibility from confirmatory data; **d**) upper bound of caller reproducibility from confirmatory data.

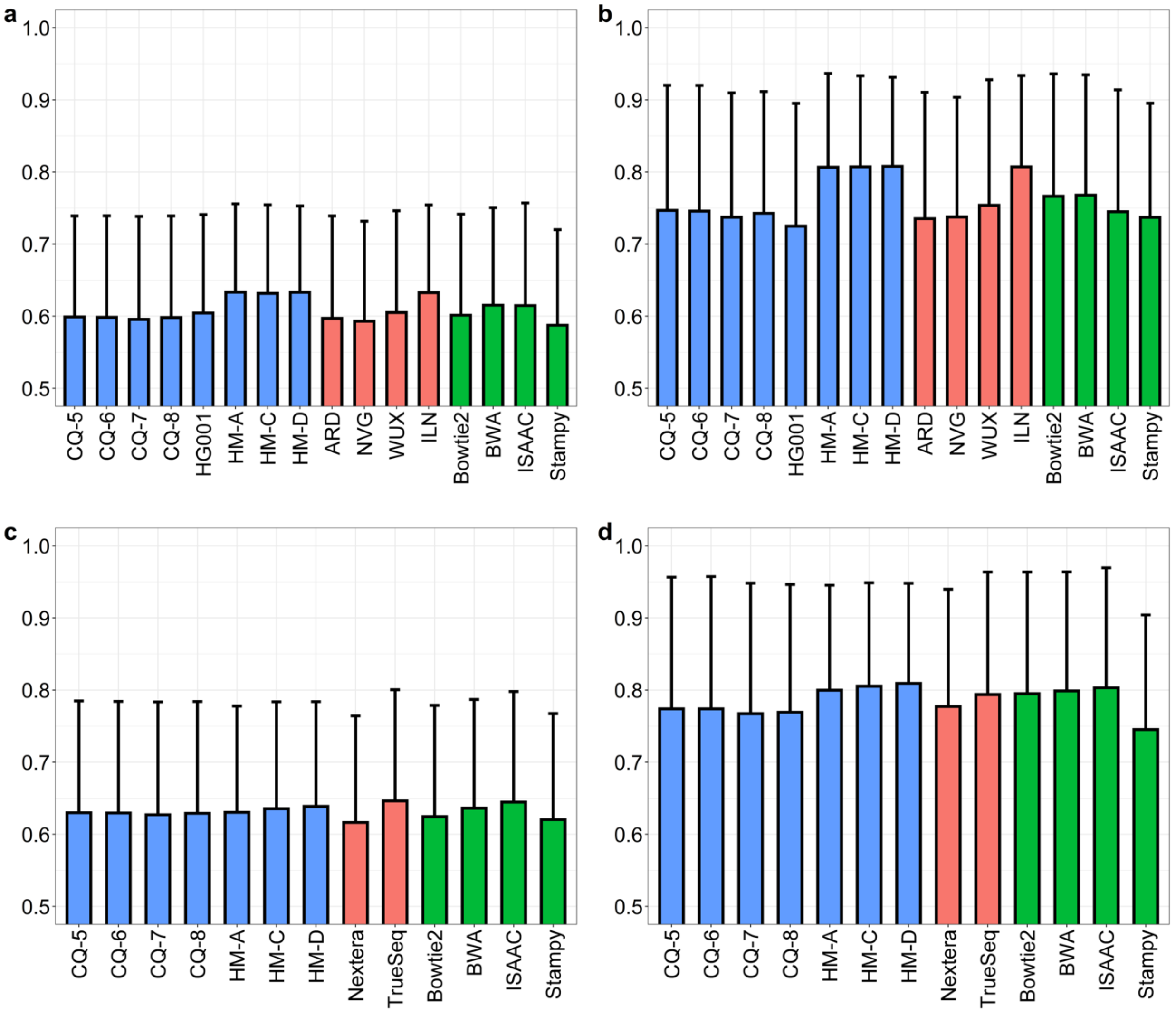

Fig. S28. Caller reproducibility in deletions (y-axes). The bars represent means and the sticks give standard deviations of caller reproducibility values for the samples (blue, HM-A: NA10835; HM-C: NA12248; HM-D: NA12249; HG001: GIAB reference sample NA12878; CQ-5 and CQ-6: Chinese quartet twin daughters; CQ-7: Chinese quartet father; CQ8: Chinese mother), labs/library kits (red) and aligners (green) indicated at the x-axes. **a**) lower bound of caller reproducibility from original data; **b**) upper bound of caller reproducibility from original data; **c**) lower bound of caller reproducibility from confirmatory data; **d**) upper bound of caller reproducibility from confirmatory data.

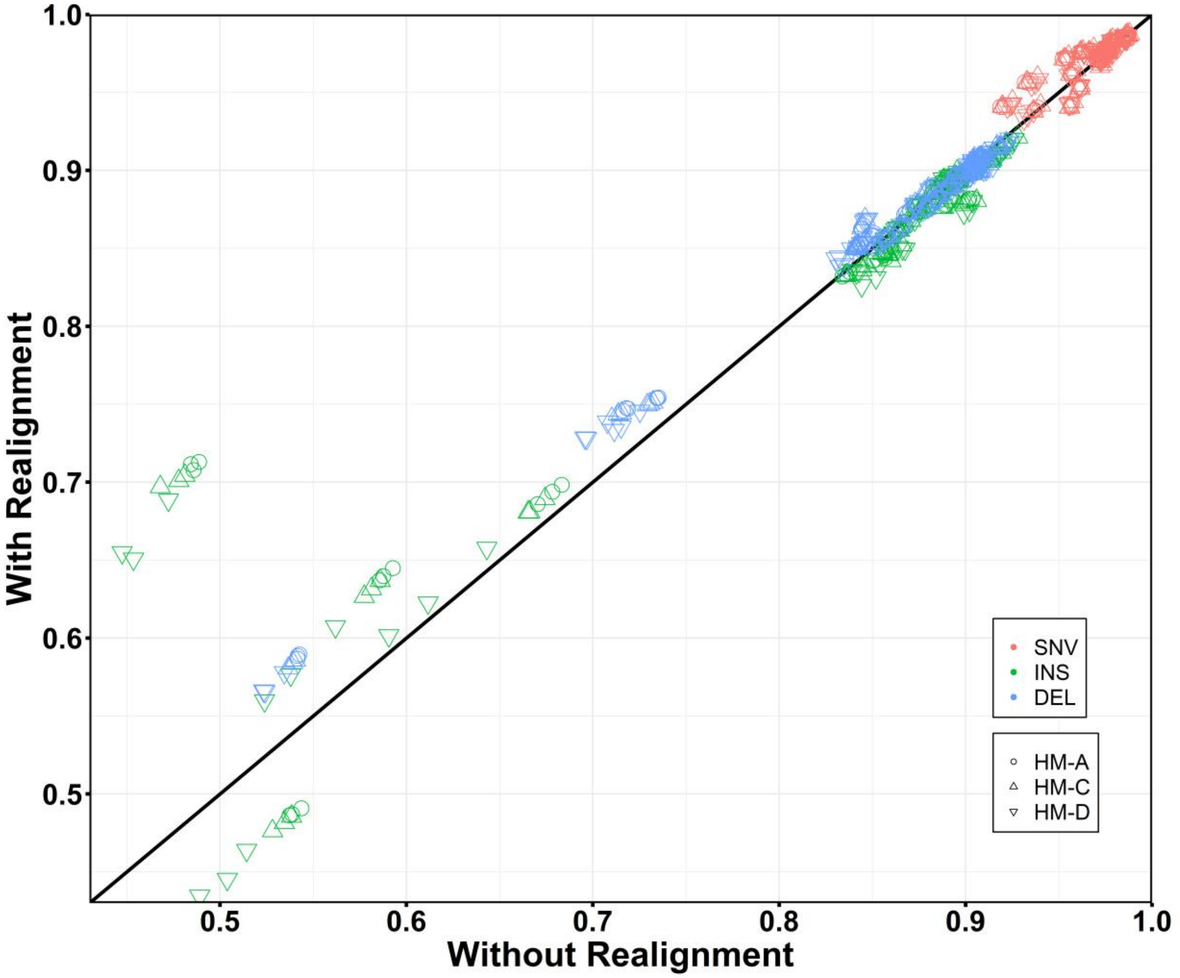

Fig. S29. GATK impact on repeatability of original HapMap data without filtering by HRRs. The points represent repeatability for pipelines with GATK realignment and without GATK realignment for various sample sets. Three type of variants are colored in three different colors: red for SNV, green for Insertion and blue for Deletion. Different samples are presented in different shapes. The x-axis depicts repeatability calculated from pipelines without GATK realignment in their variant calling progress, while y-axis represents repeatability from the related pipeline (the same aligner and caller) with GATK realignment.

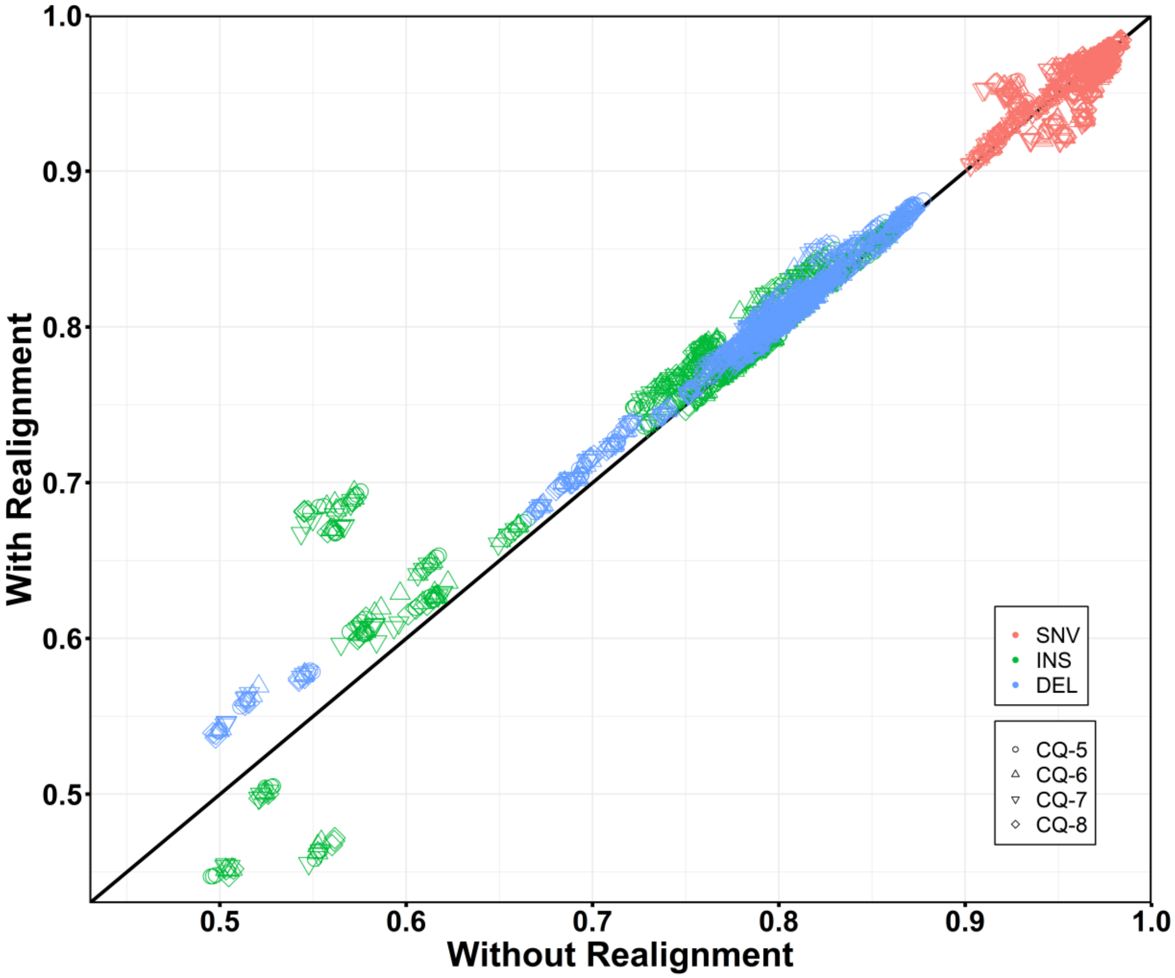

Fig. S30. GATK impact on repeatability of original CQ data without filtering by HRRs. The points represent repeatability for pipelines with GATK realignment and without GATK realignment for various sample sets. Three type of variants are colored in three different colors: red for SNV, green for Insertion and blue for Deletion. Different samples are presented in different shapes. The x-axis depicts repeatability calculated from pipelines without GATK realignment in their variant calling progress, while y-axis represents repeatability from the related pipeline (the same aligner and caller) with GATK realignment.

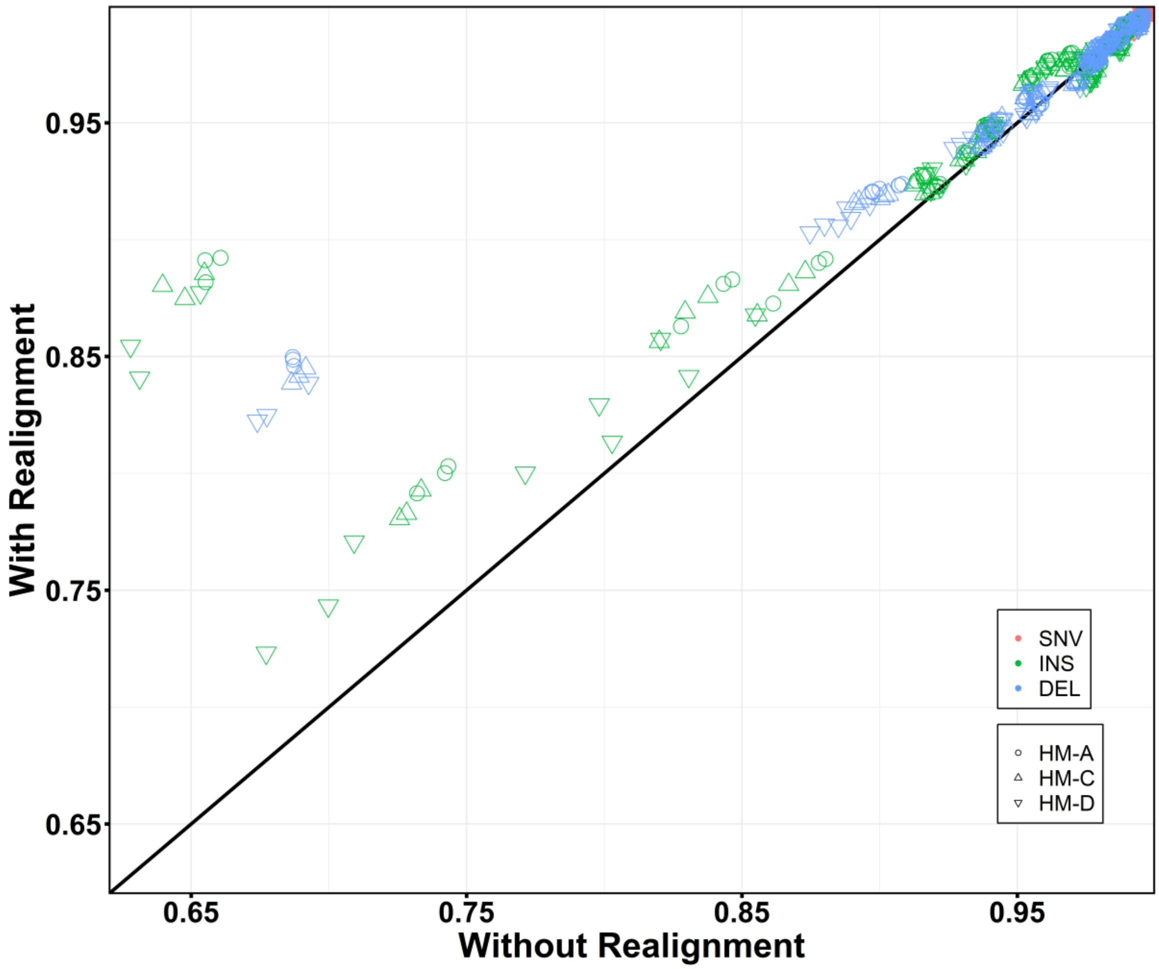

Fig. S31. GATK impact on repeatability of original HapMap data with filtering by HRRs. The points represent repeatability for pipelines with GATK realignment and without GATK realignment for various sample sets. Three type of variants are colored in three different colors: red for SNV, green for Insertion and blue for Deletion. Different samples are presented in different shapes. The x-axis depicts repeatability calculated from pipelines without GATK realignment in their variant calling progress, while y-axis represents repeatability from the related pipeline (the same aligner and caller) with GATK realignment.

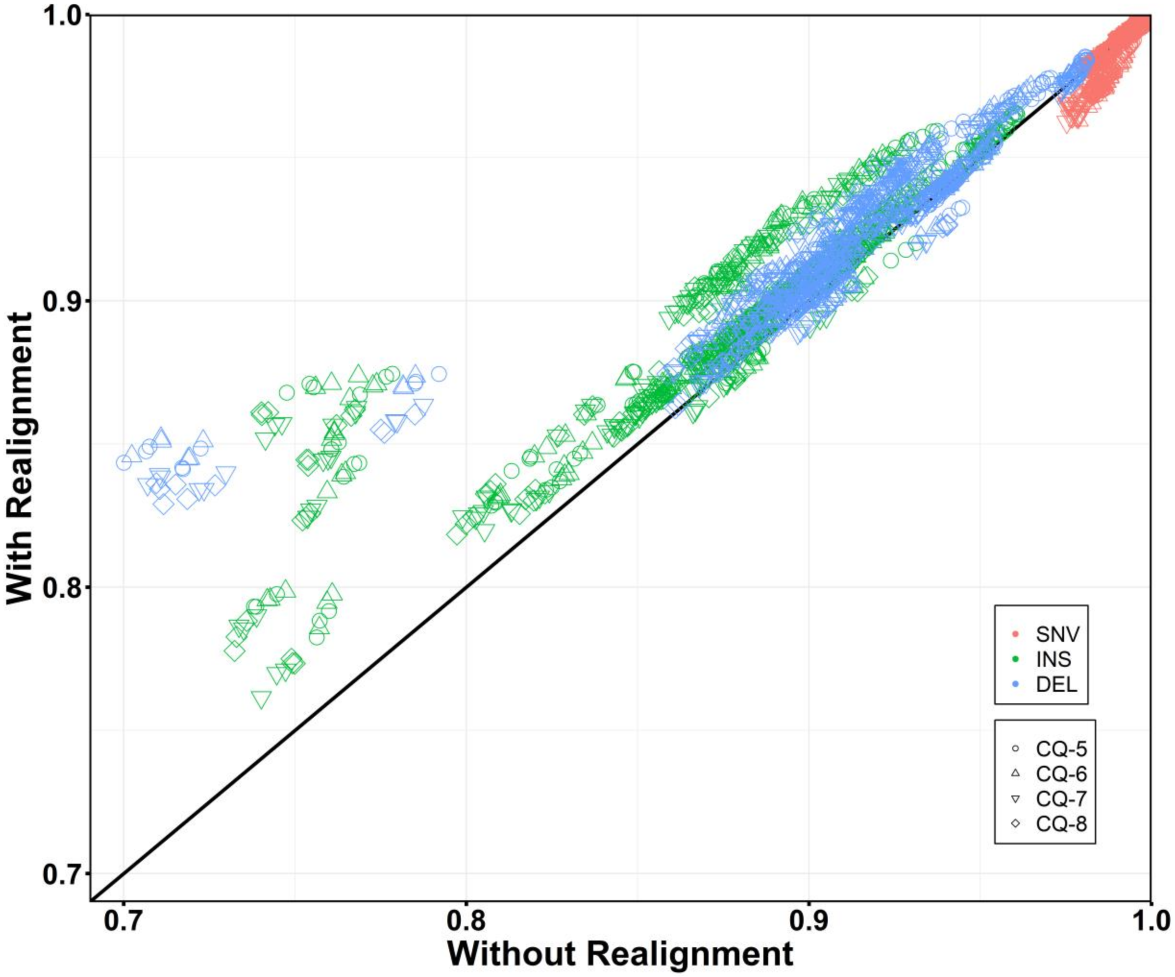

Fig. S32. GATK impact on repeatability of original CQ data with filtering by HRRs. The points represent repeatability for pipelines with GATK realignment and without GATK realignment for various sample sets. Three type of variants are colored in three different colors: red for SNV, green for Insertion and blue for Deletion. Different samples are presented in different shapes. The x-axis depicts repeatability calculated from pipelines without GATK realignment in their variant calling progress, while y-axis represents repeatability from the related pipeline (the same aligner and caller) with GATK realignment.

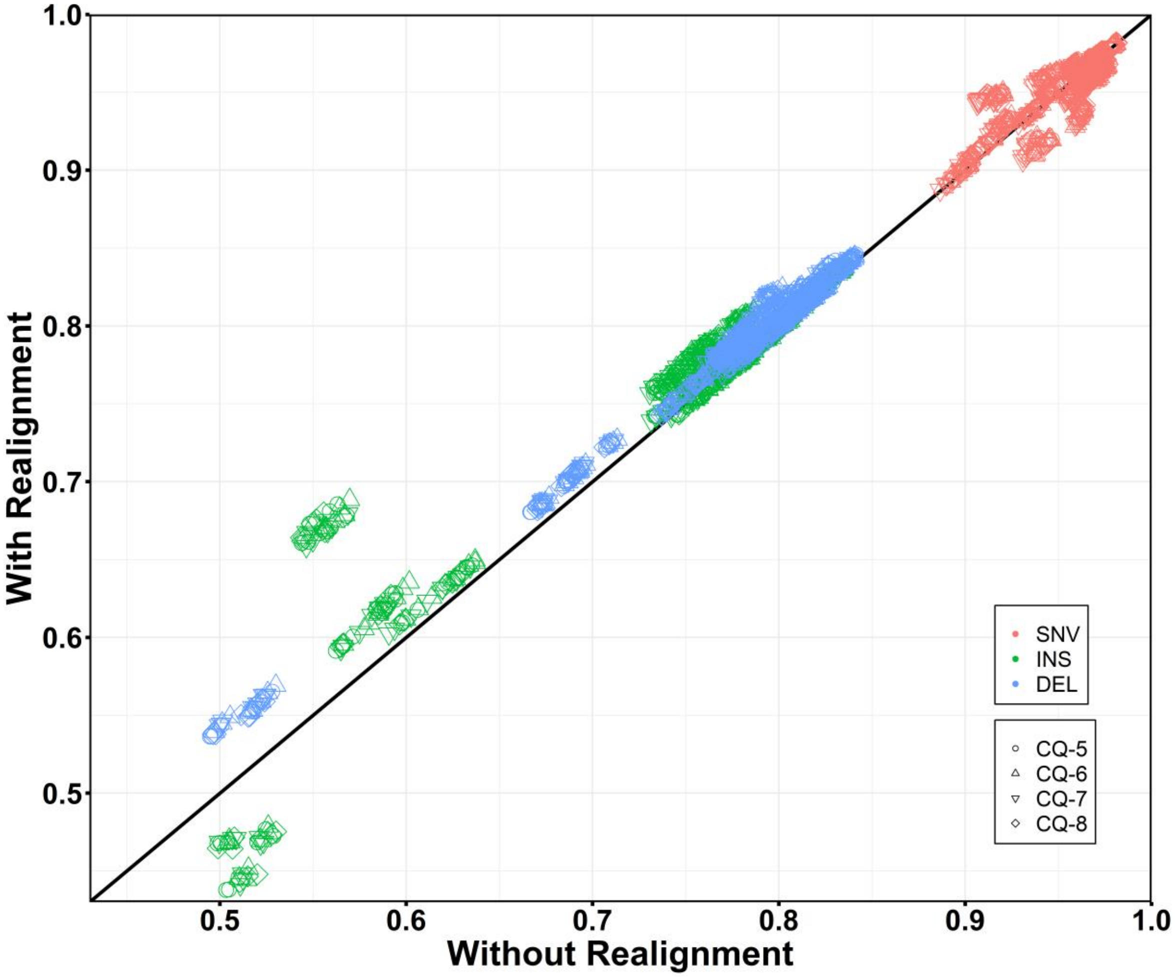

Fig. S33. Scatter plot of the impact of GATK realignment on lab reproducibility for original CQ data without filtering by HRRs. The points represent lab reproducibility of variants called from alignments with GATK realignment and without GATK realignment. Three type of variants are colored in three different colors: red for SNV, green for Insertion and blue for Deletion. Different samples are presented in different shapes. The x-axis depicts lab reproducibility calculated from variants obtained without GATK realignment, while the y-axis represents lab reproducibility of variants obtained with GATK realignment.

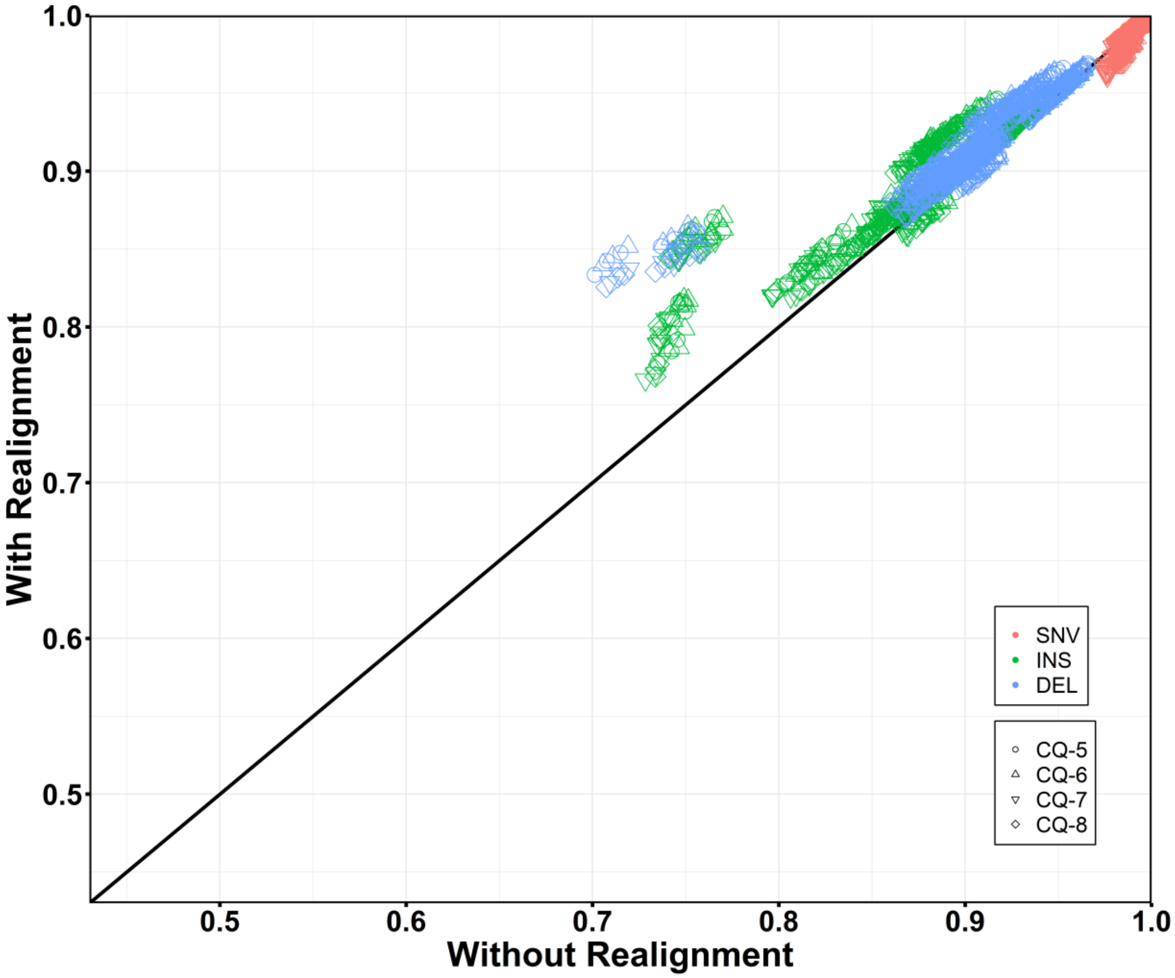

Fig. S34. Scatter plot of the impact of GATK realignment on lab reproducibility for original CQ data with filtering by HRRs. The points represent lab reproducibility of variants called from alignments with GATK realignment and without GATK realignment. Three type of variants are colored in three different colors: red for SNV, green for Insertion and blue for Deletion. Different samples are presented in different shapes. The x-axis depicts lab reproducibility calculated from variants obtained without GATK realignment, while the y-axis represents lab reproducibility of variants obtained with GATK realignment.

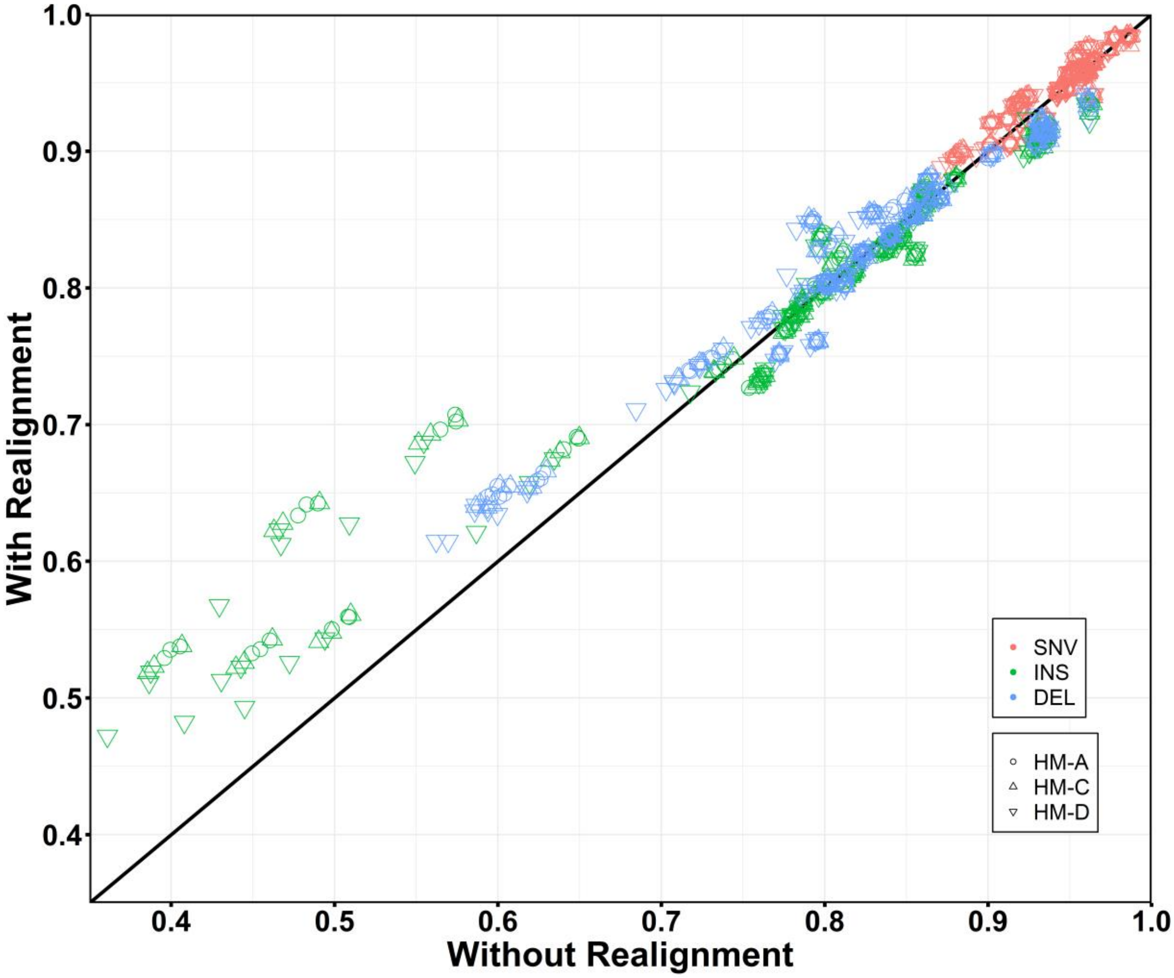

Fig. S35. Scatter plot for the impact of GATK realignment on aligner reproducibility of original HapMap data without filtering by HRRs. The points represent aligner reproducibility for calling pipelines with GATK realignment and without GATK realignment for various sample sets (HM-A: NA10835; HM-C: NA12248; HM-D: NA12249). Three type of variants are colored in three different colors: red for SNV, green for Insertion and blue for Deletion. Different samples are presented in different shapes. The x-axis depicts aligner reproducibility without GATK realignment in variant calling, while the y-axis represents aligner reproducibility with GATK realignment.

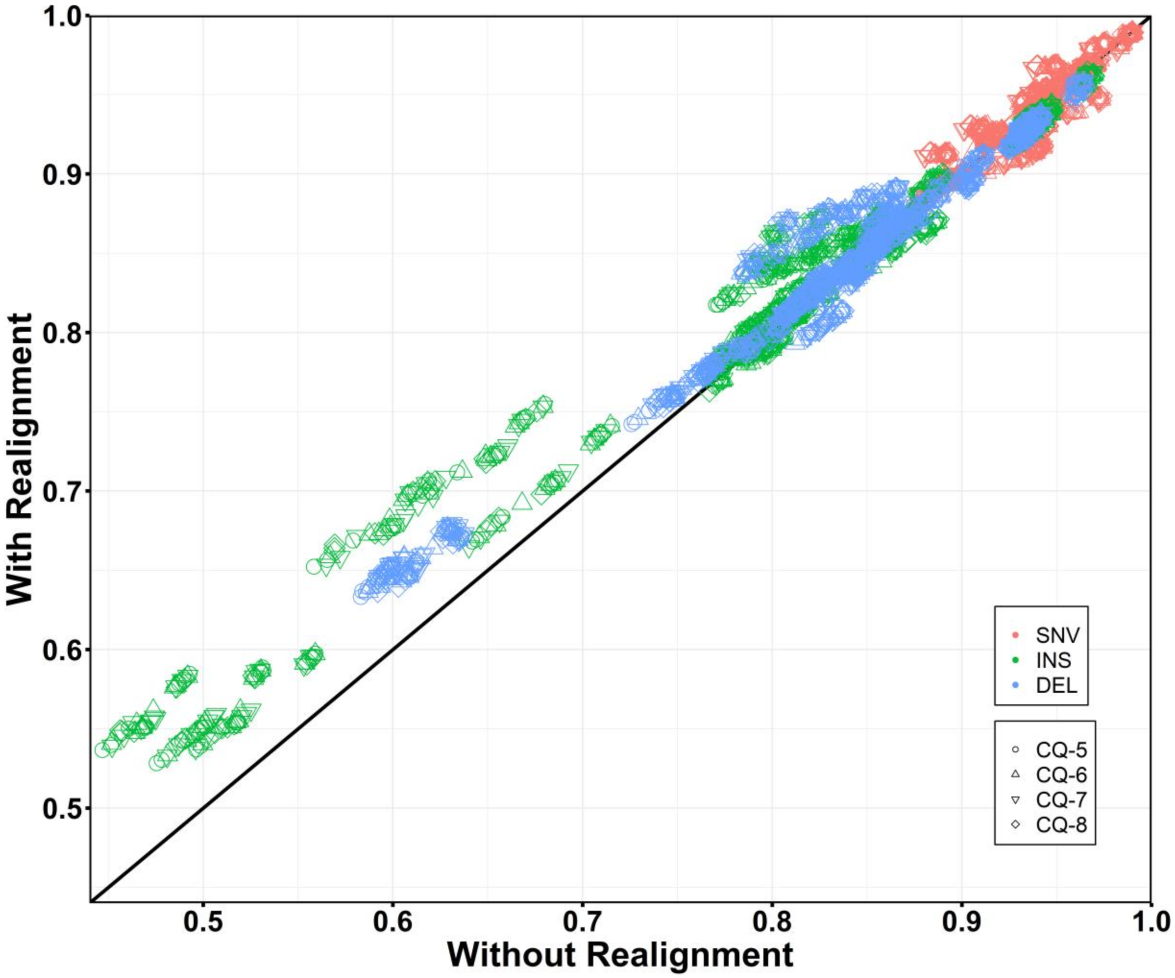

Fig. S36. Scatter plot for the impact of GATK realignment on aligner reproducibility of original CQ data without filtering by HRRs. The points represent aligner reproducibility for calling pipelines with GATK realignment and without GATK realignment for various sample sets (CQ-5 and CQ-6: Chinese quartet twin daughters; CQ-7: Chinese quartet father; CQ8: Chinese quartet mother). Three type of variants are colored in three different colors: red for SNV, green for Insertion and blue for Deletion. Different samples are presented in different shapes. The x-axis depicts aligner reproducibility without GATK realignment in variant calling, while the y-axis represents aligner reproducibility with GATK realignment.

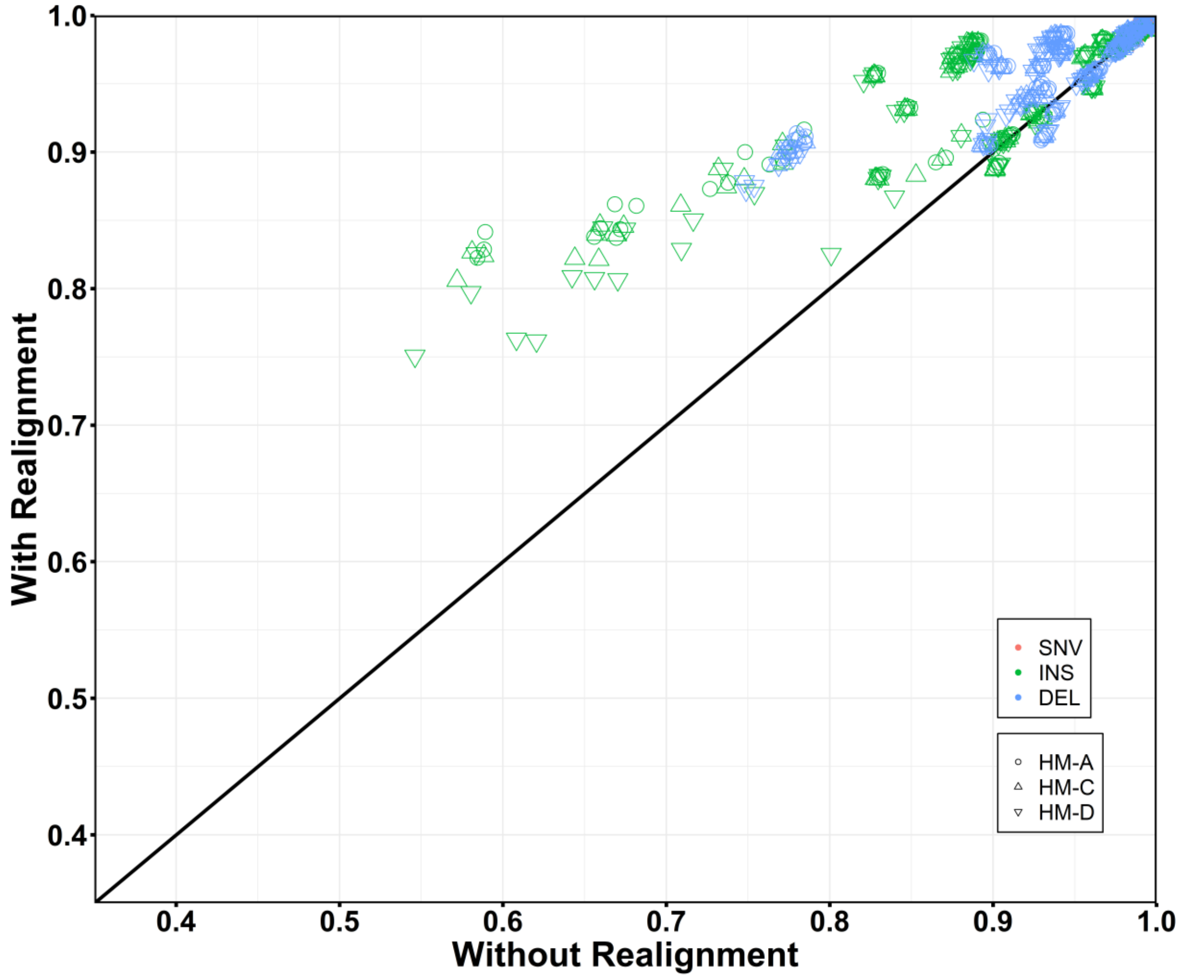

Fig. S37. Scatter plot for the impact of GATK realignment on aligner reproducibility of original HapMap data with filtering by HRRs. The points represent aligner reproducibility for calling pipelines with GATK realignment and without GATK realignment for various sample sets (HM-A: NA10835; HM-C: NA12248; HM-D: NA12249). Three type of variants are colored in three different colors: red for SNV, green for Insertion and blue for Deletion. Different samples are presented in different shapes. The x-axis depicts aligner reproducibility without GATK realignment in variant calling, while the y-axis represents aligner reproducibility with GATK realignment.

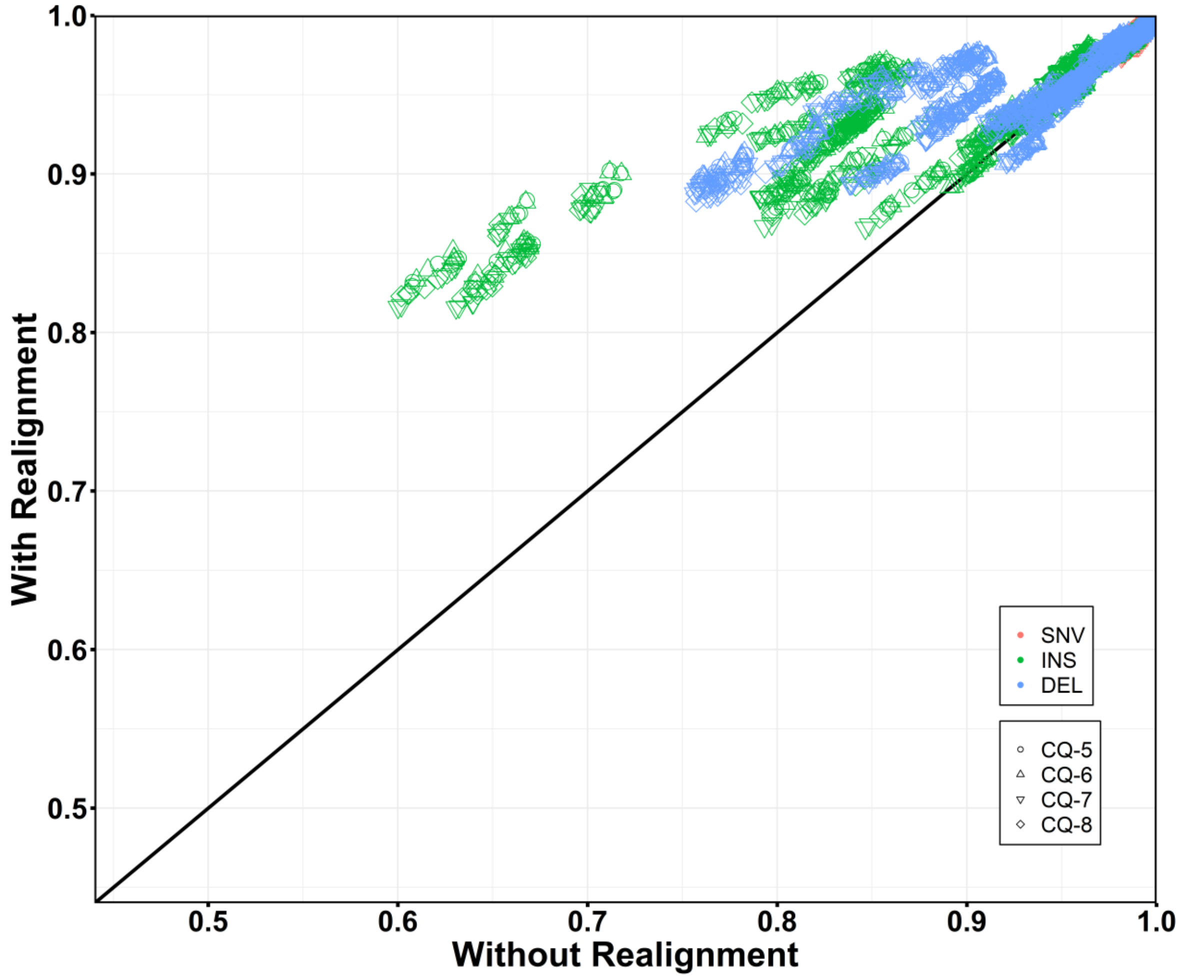

Fig. S38. Scatter plot for the impact of GATK realignment on aligner reproducibility of original CQ data with filtering by HRRs. The points represent aligner reproducibility for calling pipelines with GATK realignment and without GATK realignment for various sample sets (CQ-5 and CQ-6: Chinese quartet twin daughters; CQ-7: Chinese quartet father; CQ8: Chinese quartet mother). Three type of variants are colored in three different colors: red for SNV, green for Insertion and blue for Deletion. Different samples are presented in different shapes. The x-axis depicts aligner reproducibility without GATK realignment in variant calling, while the y-axis represents aligner reproducibility with GATK realignment.

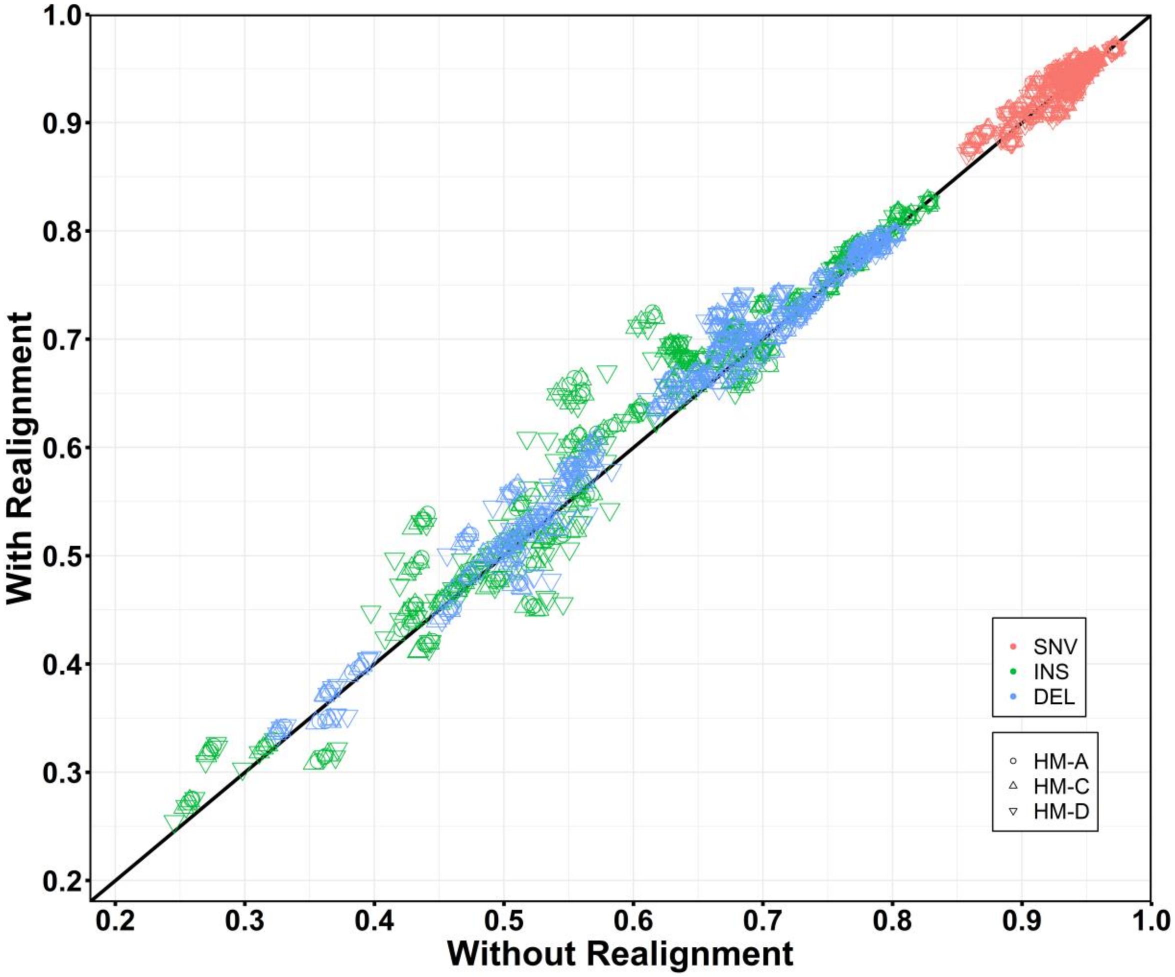

Fig. S39. Scatter plot for the impact of GATK realignment on caller reproducibility of original HapMap data without filtering by HRRs. The points represent caller reproducibility for calling pipelines with GATK realignment and without GATK realignment for various sample sets (HM-A: NA10835; HM-C: NA12248; HM-D: NA12249). Three type of variants are colored in three different colors: red for SNV, green for Insertion and blue for Deletion. Different samples are presented in different shapes. The x-axis depicts caller reproducibility without GATK realignment in variant calling, while the y-axis represents caller reproducibility with GATK realignment.

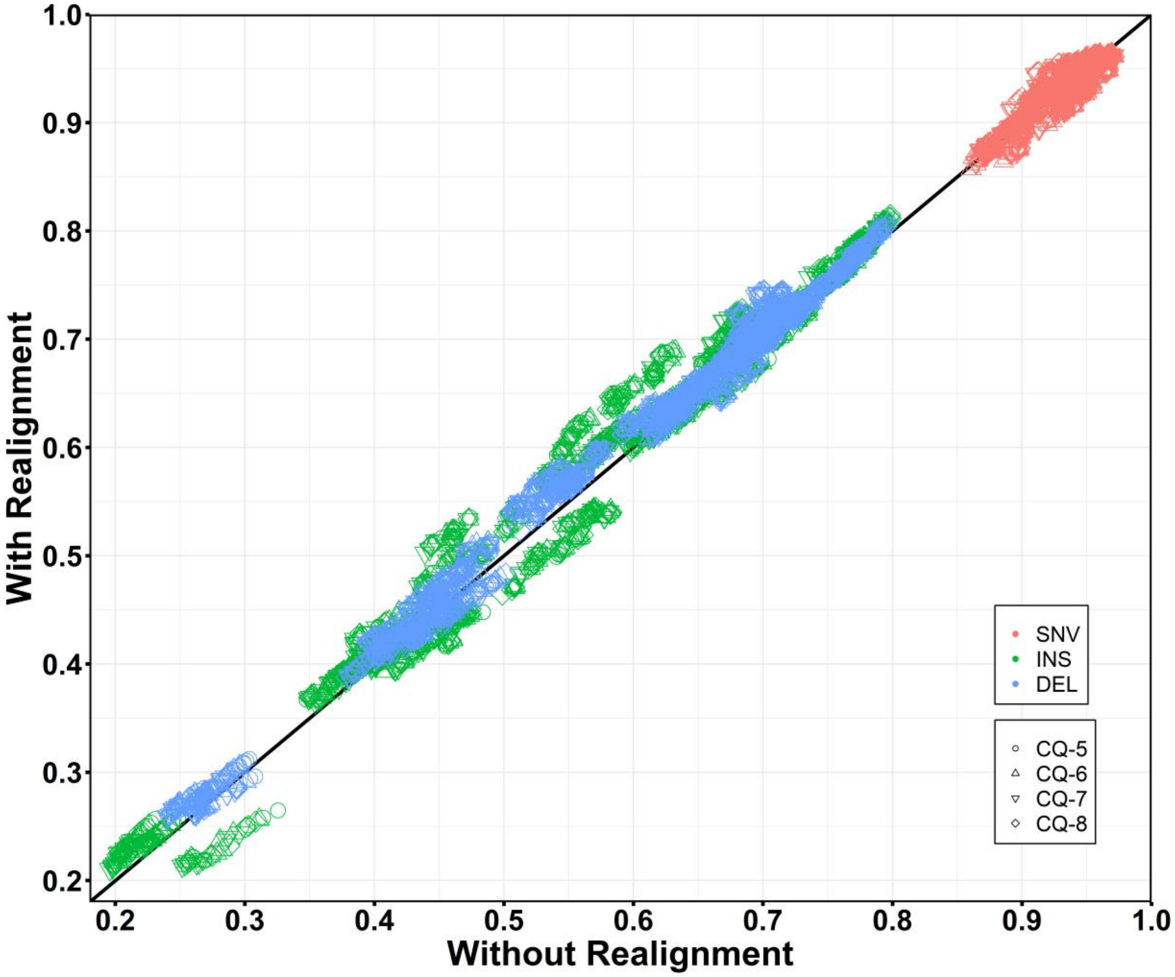

Fig. S40. Scatter plot for the impact of GATK realignment on caller reproducibility of original CQ data without filtering by HRRs. The points represent caller reproducibility for calling pipelines with GATK realignment and without GATK realignment for various sample sets (CQ-5 and CQ-6: Chinese quartet twin daughters; CQ-7: Chinese quartet father; CQ8: Chinese quartet mother). Three type of variants are colored in three different colors: red for SNV, green for Insertion and blue for Deletion. Different samples are presented in different shapes. The x-axis depicts caller reproducibility without GATK realignment in variant calling, while the y-axis represents caller reproducibility with GATK realignment.

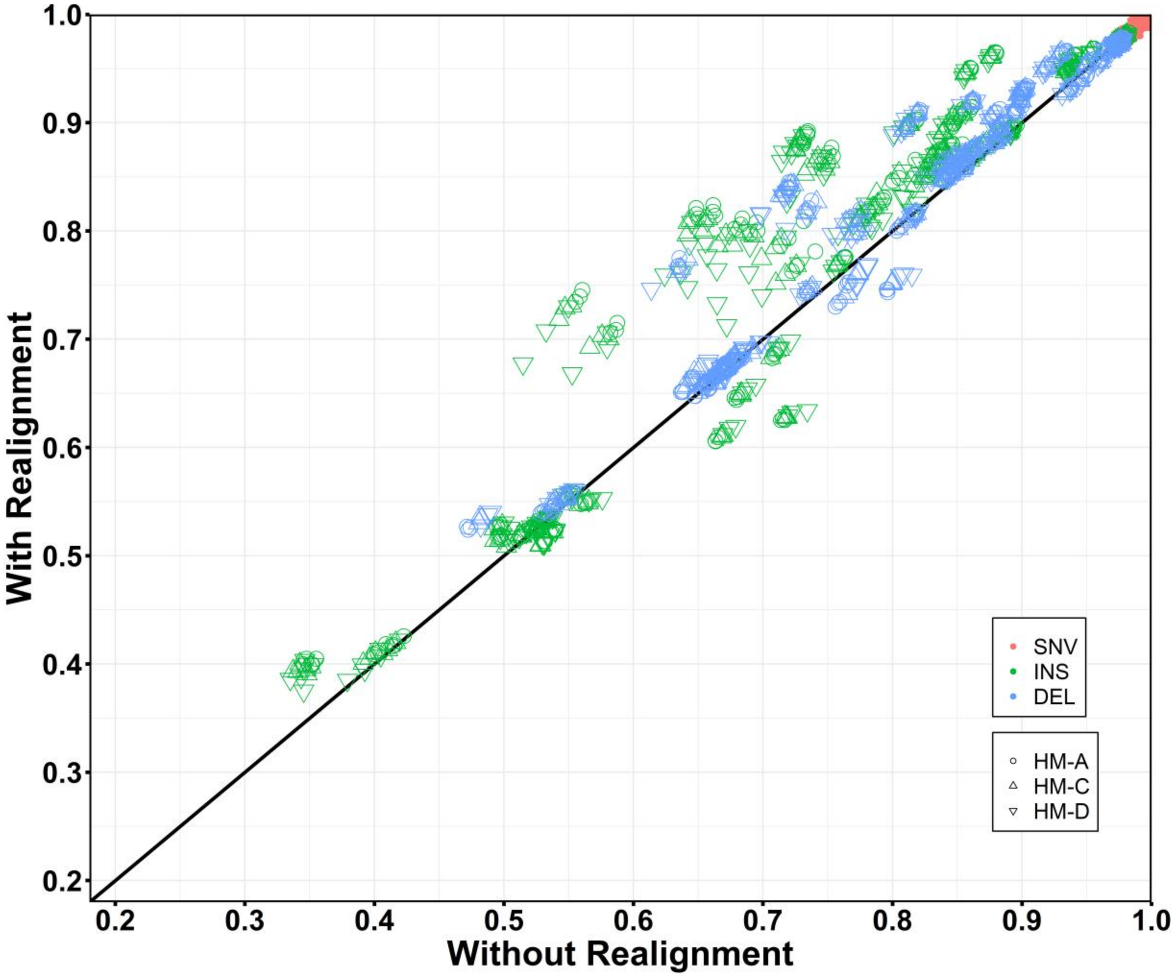

Fig. S41. Scatter plot for the impact of GATK realignment on caller reproducibility of original HapMap data with filtering by HRRs. The points represent caller reproducibility for calling pipelines with GATK realignment and without GATK realignment for various sample sets (HM-A: NA10835; HM-C: NA12248; HM-D: NA12249). Three type of variants are colored in three different colors: red for SNV, green for Insertion and blue for Deletion. Different samples are presented in different shapes. The x-axis depicts caller reproducibility without GATK realignment in variant calling, while the y-axis represents caller reproducibility with GATK realignment.

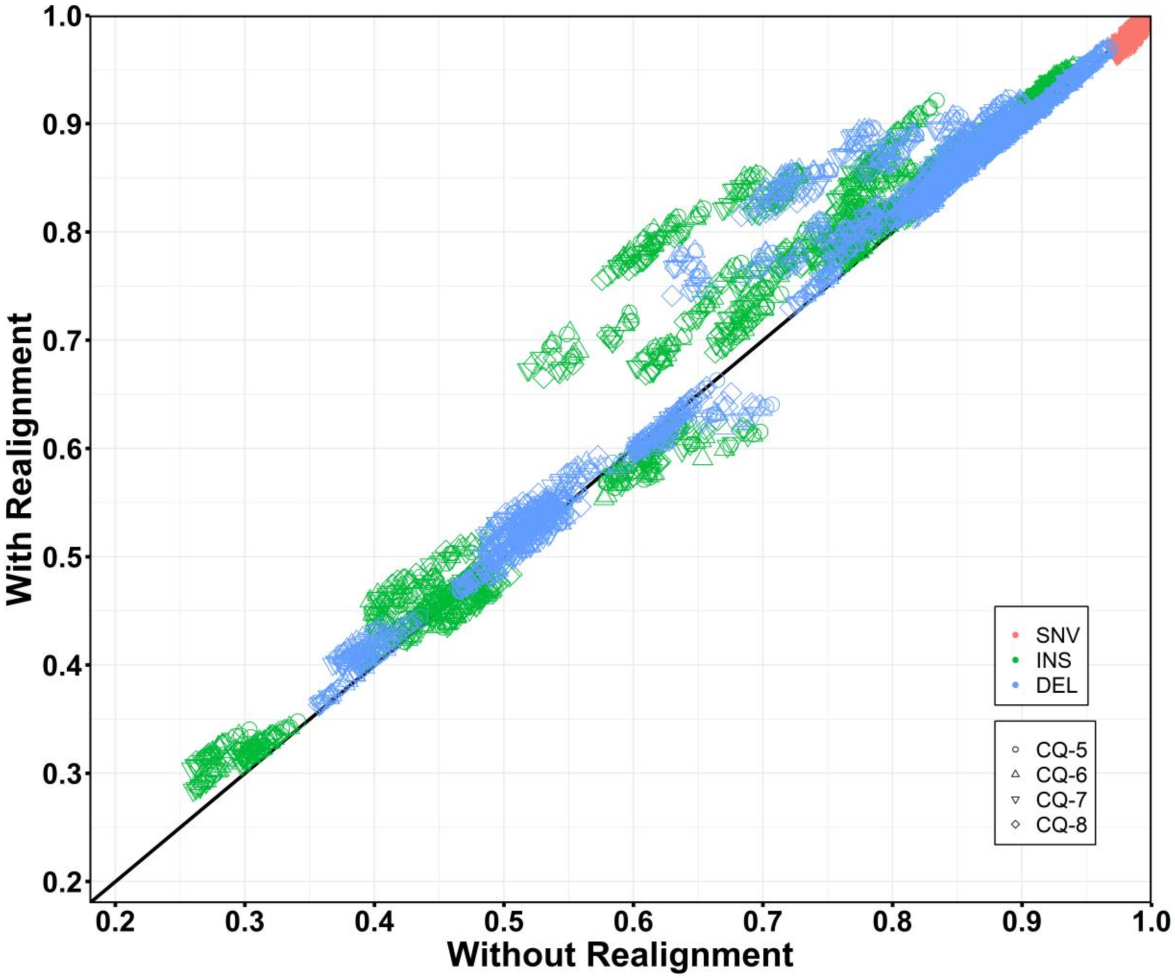

Fig. S42. Scatter plot for the impact of GATK realignment on caller reproducibility of original CQ data with filtering by HRRs. The points represent caller reproducibility for calling pipelines with GATK realignment and without GATK realignment for various sample sets (CQ-5 and CQ-6: Chinese quartet twin daughters; CQ-7: Chinese quartet father; CQ8: Chinese quartet mother). Three type of variants are colored in three different colors: red for SNV, green for Insertion and blue for Deletion. Different samples are presented in different shapes. The x-axis depicts caller reproducibility without GATK realignment in variant calling, while the y-axis represents caller reproducibility with GATK realignment.
